# Comparing Eight Parameter Estimation Methods for the Ratcliff Diffusion Model Using Free Software

**DOI:** 10.3389/fpsyg.2020.484737

**Published:** 2020-09-29

**Authors:** Rainer W. Alexandrowicz, Bartosz Gula

**Affiliations:** Institute for Psychology, Universitaet Klagenfurt, Klagenfurt, Austria

**Keywords:** diffusion model, parameter estimation, parameter recovery, method comparison, simulation study

## Abstract

The Ratcliff Diffusion Model has become an important and widely used tool for the evaluation of psychological experiments. Concurrently, numerous programs and routines have appeared to estimate the model's parameters. The present study aims at comparing some of the most widely used tools with special focus on freely available routines (i.e., open source). Our simulations show that (1) starting point and non-decision time were recovered better than drift rate, (2) the Bayesian approach outperformed all other approaches when the number of trials was low, (3) the Kolmogorov-Smirnov and χ^2^ approaches revealed more bias than Bayesian or Maximum Likelihood based routines, and (4) EZ produced substantially biased estimates of threshold separation, non-decision time and drift rate when starting point z ≠ *a*/2. We discuss the implications for the choice of parameter estimation approaches for real data and suggest that if biased starting point cannot be excluded, EZ will produce deviant estimates and should be used with great care.

## 1. Introduction

The Diffusion Model (DM; Ratcliff, 1978, 2013) allows for modelling both response time and accuracy of fast human decisions (Forstmann et al., 2016; Ratcliff et al., 2016). Typical applications are found in experimental psychology, especially in the context of the Two-Alternative-Forced-Choice (2AFC) paradigm (Laming, 1968; Arnold et al., 2015; Aschenbrenner et al., 2016; Dirk et al., 2017; Klauer et al., 2007; Mayerl et al., 2019; Mulder et al., 2010; Park and Starns, 2015; Schubert et al., 2019; Schuch, 2016; Voss et al., 2013; Yap et al., 2015). It assumes a decision process based on the accumulation of evidence triggered by a stimulus until one of two decision boundaries reflecting the two decision options is reached.

**Figure 1:**
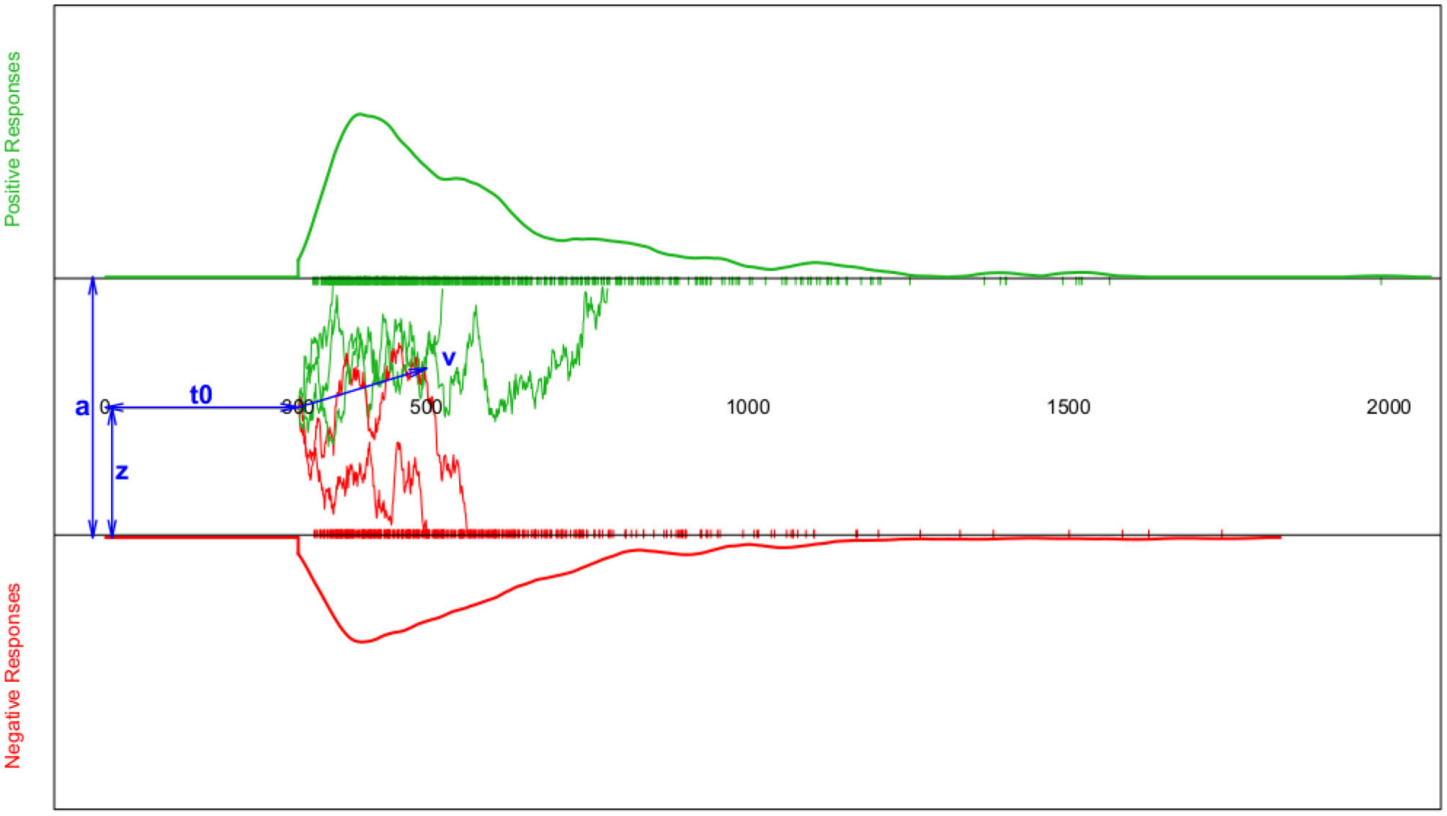
Schematic depiction of the diffusion model and the resulting response time (RT) distributions. The horizontal axis represents the response time. The top section (green curve) shows the RT density estimate for positive responses and the bottom section (red curve) the RT density estimate for negative responses (both accompanied by a rug-plot along the RT-axis); Note that for reaction times shorter than *t*_0_, the probability of a response is set to zero. The middle section sketches a few examples of the trajectories representing the information accumulation process assumed to follow a random walk. The blue double-headed arrows indicate the four main model parameters *a* (boundary separation), *z* (starting point), *t*_0_ (or *T*ER; encoding-and-reaction time; non-decision components), and ν (drift parameter of the random walk).

This process is modelled as a Wiener diffusion process, which can be described by four main parameters (cf. Figure 1): The boundary separation parameter *a* describes the distance between the two decision boundaries and thus reflects the subject's response caution: The larger *a*, the more evidence is required before choosing one of the two response alternatives. The drift parameter ν reflects the average accumulation rate per time unit, which is an indication of the speed of the decision process. The starting point 0 < *z* < *a* is the location relative to the two decision boundaries, at which the decision process starts. If the respondent expects a priori (e.g., by instruction) that the upper decision option is more likely, *z* will be shifted toward *a*. If no expectation exists, *z* = *a*/2. Finally, all time components not involved in decision making are summarized in the *T*ER (or *t*_0_; encoding and reaction time) parameter.

The model equation for the probability of a response at the lower boundary is

(1) $$\begin{array}{l} P(-|a,z,\nu )=\frac{{\text{e}}^{-(2\nu a/s^{2})}-{\text{ e}}^{-(2\nu z/s^{2})}}{{\text{e}}^{-(2\nu a/s^{2})}-1}, \end{array}$$

and the finishing time density for negative responses is modelled as

(2) $$\begin{array}{l} g_{-}\left(t,a,z,\nu \right)=\frac{\pi s^{2}}{a^{2}}{\text{e}}^{-\frac{z\nu }{s^{2}}}\overset{\infty }{\underset{k=1}{\sum }}k\sin\left(\frac{\pi zk}{a}\right)\times {\text{e}}^{-\frac{1}{2}\left(\frac{\nu^{2}}{s^{2}}+\frac{\pi^{2}k^{2}s^{2}}{a^{2}}\right)t} \end{array}$$

(cf. Ratcliff, 1978, p. 70, Equations (A8) and (A9), notation adapted). The probability of and density for positive responses is obtained analogously by applying ν_+_ = −ν and *z*_+_ = *a* − *z*. The term *s*^2^ denotes the variance of the Brownian motion within one trial (therefore termed intra-trial variability of the drift), which is not a model parameter, but rather a constant, which has to be set to an appropriate value prior to parameter estimation. The choice of *s* is not critical, as it concerns only the scale of the estimated parameters; two values are often observed, *s* = 1 and *s* = 0.1. A detailed derivation of the model equations can be found in Busemeyer and Diederich (2010).

Alongside to these four main parameters, three additional variability parameter were introduced to cover between-trials variability of the main parameters. These are *s*_ν_ (for varying drift), *s*_*z*_ (for varying starting points), and *s*_*t*_ (for varying encoding and reaction times). These three parameters are not part of the model Equations (1) and (2) but have to be obtained by numerical integration (cf. Ratcliff and Tuerlinckx, 2002).

See Alexandrowicz (2020) for details regarding the psychological interpretation of the model parameters and www.dmvis.at for the Diffusion Model Visualizer (DMV), an interactive tool visualizing the effect each model parameter (including the variability parameters) has on the resulting response probabilities and response time densities.

### 1.1. Parameter Estimation Methods

Various methods for estimating the parameters of the DM exist coevally following various different estimation principles. We introduce them below, as they are essential for the present study.

#### 1.1.1. A Closed Form Expression and Its Enhanced Version

A closed form solution (EZ) has been developed by Wagenmakers et al. (2007), which makes use of three descriptive statistics, viz. mean of correct responses (*MRT*), variance of correct responses (*VRT*), and proportion of correct responses (*P*_*c*_), allowing for an estimation of the boundary separation *a*, the drift parameter ν, and the non-decision time component *T*ER; no provisions are made to estimate the response bias *z* or any of the three variability parameters *s*_ν_, *s*_*T*ER_, and *s*_*z*_. Rather, the bias is fixed at *z* = *a*/2 and the variability parameters at zero.

In a critical assessment, Ratcliff (2008) showed that contaminant responses (i.e., response times distorting the true distribution, like lapses of attention, distraction, anticipation, or fast guesses) could have a large impact on the response time variance and thus lead to inaccurate parameter estimates. Moreover, he demonstrated that the EZ method delivers larger parameter estimates' variability than the χ^2^-Method (CS; see section 1.1.2) and that the two methods appear to be sensitive to different characteristics of the data. Also model misspecification regarding the parameters fixed in the EZ model may lead to deviant parameter estimates. In their rejoinder, Wagenmakers et al. (2008b) proposed a robust EZ method, applying mixture modelling to improve the handling of RT contaminants, on top of that they introduced an extended algorithm, EZ2, allowing for estimating the response bias *z* as well. This modified method, termed EZ2 (Grasman et al., 2009), allows for estimating the bias parameter *z* as well, but only in designs providing for more than one stimulus condition.

#### 1.1.2. Comparing Observed and Expected Frequencies Using the χ^2^ Statistic

Ratcliff and Tuerlinckx (2002) proposed an estimation method, in which they binned the reaction time distributions separately for both the correct and incorrect decisions using the 0.1, 0.3, 0.5, 0.7, and 0.9 quantiles of the respective distributions, thus yielding the observed frequencies of each bin. The expected frequencies of each bin are obtained from the density function according to Equation (2) (for both the correct and the incorrect responses) given a candidate set of model parameters. Observed and expected frequencies are compared with the χ^2^-statistic across all bins of both distributions, $CS=\underset{b}{\sum }{\left(\text{ob}{\text{s}}_{b}-\text{ex}{\text{p}}_{b}\right)}^{2}/\text{ex}{\text{p}}_{b}$, with *b* = 1…*B* denoting the bin index. Parameter estimates are updated in an iteration loop using the simplex downhill method of Nelder and Mead (1965) to minimize the value of the CS-statistic. The authors outline the method in Appendix B of their paper (pp. 479–481).

The advantage of this method is that the CS-statistic allows for an inferential assessment of model fit using the (1 − α/2)-quantile of a χ^2^-distribution with *df* = *C*(*B* − 1) − *P*, with *C* representing the number of experimental conditions, *B* the number of bins across both distributions, *P* the number of estimated parameters, and α denotes the risk of a type-I-error. However, from a theoretical point of view, the CS-statistic is only approximately χ^2^-distributed, because the bins are determined by the data (cf. Ratcliff and Childers, 2015, p. 252).

A disadvantage of this method could be the loss of information by aggregating data into bins. Note that the binning proposed by Ratcliff and Tuerlinckx (2002) remains—albeit reasonable—still arbitrary and a different choice of bins might result in different parameter estimates. Voss et al. (2004) pointed out that too many bins may introduce chance dependence due to small bin frequencies, whereas too few bins may cause loss of information (p. 1217).

#### 1.1.3. Comparing Observed and Expected Cumulative Distribution Functions of the Response Times Using the Kolmogorov-Smirnov Statistic

**Figure 2:**
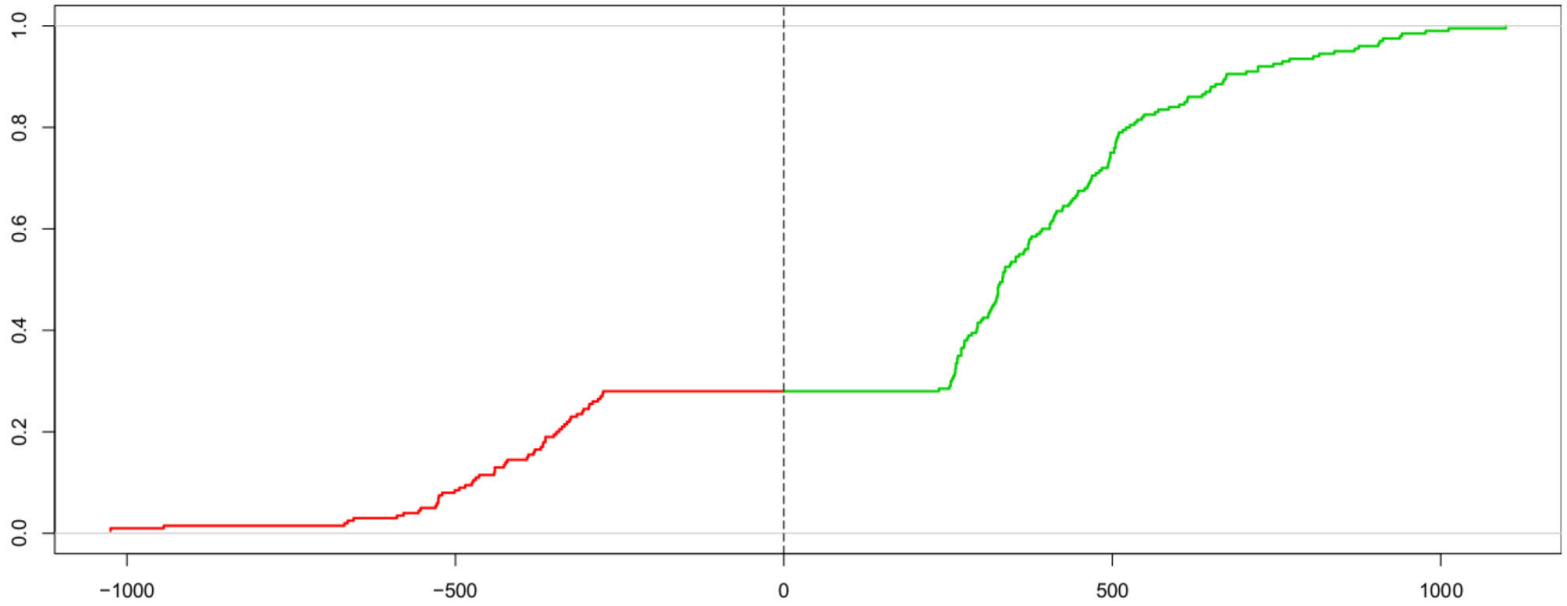
Super-curve of the combined cumulative distribution function of the data used in Figure 1. The horizontal axis depicts the reaction time (with response times of lower threshold passes mirrored at the origin). The vertical axis represents the probability *P*(*X* < *x*). The left (red) part of this curve represents the mirrored cumulative distribution function of the incorrect responses and the right (green) part the cdf of the correct ones. The vertical dashed line indicates the point, where the two separate curves have been joined. The combined curve intersects this line at height 1 − *P*_*c*_ (which, in this case, is 0.28, corresponding to 28 % incorrect responses).

The Kolmogorov-Smirnov statistic (KS; Kolmogorov, 1933, 1992; Smirnov, 1939; Stephens, 1992) allows for comparing two cumulative distribution functions using the maximum vertical distance between the two curves. Voss et al. (2004) proposed to use this measure for estimating the parameters of the DM by comparing the cumulative distribution of the observed response times and the one from the expected response times under the model given parameter estimate candidates. However, in order to determine the measure (described below) simultaneously for both response time distributions of the correct (passing the upper threshold) and the incorrect (passing the lower threshold) responses, a “trick” is applied: The two curves are put next to each other, with one mirrored (i.e., values multiplied by −1), thus forming one “super-curve” representing both cumulative distributions contiguously (the authors credit Heinrich von Weizsäcker for the hint to this trick). Figure 2 shows this super-curve for the data used in Figure 1.

The parameter estimates are obtained by iteratively fitting the parameter estimates so that the expected curve is closest to the observed one using the Nelder-Mead method. As an advantage, the K-S method also allows for an assessment of model fit by comparing the final value of the K-S-statistic *KS* = ∥*F*_obs_ − *F*_exp_∥ (*F*(·) denoting the cumulative distribution function) to the (1 − α)-quantile of the K-S distribution (which may be approximated for a given limit α by $Q_{\alpha }=\sqrt{\log\left(2/\alpha \right)/2}$).

#### 1.1.4. Maximum Likelihood Estimation

The Maximum Likelihood (ML) estimation method can be considered the most prominent principle in statistical modelling [from a so-called “frequentist” point of view; (cf. Berger and Sellke, 1987; Casella and Berger, 1987; Edwards, 1992; Royall, 2000), and section 1.1.5]. ML estimates dispose of advantageous features, most prominently consistency, efficiency, and normal distribution (e. g., Pawitan, 2001). We find a detailed account of the ML estimation for the DM in the Appendix of Ratcliff and Tuerlinckx (2002). One special issue seems worth reiterating: Because density estimates of the reaction time can only be obtained for values of *t* > *T*ER, an important regularity condition for ML estimation is violated. Therefore, the common method of finding the root(s) of the first partial derivatives of the model's likelihood function cannot be applied. Rather, general purpose numerical search algorithms are required, causing ML methods to become computationally expensive. Two prominent methods are the simplex downhill search algorithm of Nelder and Mead (1965) and the Broyden-Fletcher-Goldfarb-Shanno method (BFGS; named after the four researchers, who proposed the method independently from each other in 1970; Broyden, 1970; Fletcher, 1970; Goldfarb, 1970; Shanno, 1970).

#### 1.1.5. The (Hierarchical) Bayesian Approach

Lee et al. (2006) applied the Bayesian estimation framework to the DM, using a slightly modified model definition, in which the starting point is zero, the upper boundary is termed α, and the lower boundary β; hence, *a* = α + β and *z* = (β − α)/(α + β). They used non-informative priors, namely (with τ set to 10^−6^) *N*(0, τ) > 0 for both α and β, *N*(0, τ) for ν, and *U*(0, min(*t*)) for *T*ER. Using the program WinBUGS (see section 2.1.4) and running three chains of length 10^5^, they obtained fairly similar values for α and β, which conforms to a *z* of approximately *a*/2 in our notation. Except for a bimodality in the drift parameter posteriors, their results were comparable to a conventional analysis.

Rouder et al. (2003) applied a hierarchical Bayesian method to model response time densities using a three parameter Weibull distribution (cf. Evans et al., 2000, ch. 42.5), which accounts for the typical skewedness of such densities in a manner similar to the DM.

#### 1.1.6. Further Estimation Principles

Ratcliff and Tuerlinckx (2002) also proposed a Weighted Least Squares (WLS) method, minimizing the squared difference between observed and predicted frequencies of reaction time quantiles for both correct and incorrect decisions. The weights are obtained from the standard deviations within the bins arising from the quantile points. However, this approach has proven less than convincing, as the authors have shown in a simulation study that the parameter estimates exhibited bias and a larger standard error than the CS method. Therefore, they recommend the WLS method as an exploratory tool, especially when other methods (mainly referring to the CS method) do not provide satisfactory fit.

Besides the Pearson χ^2^ statistic as quoted in section 1.1.2, there is also the likelihood ratio statistic $G^{2}=\underset{b}{\sum }\text{ob}{\text{s}}_{b}\log\left(\text{ob}{\text{s}}_{b}/\text{ex}{\text{p}}_{b}\right)$ (e.g., Ratcliff, 2013, p. 39; notation adapted), which follows the same χ^2^ distribution under the null-hypothesis of model fit. Hence, it also delivers approximately the same results (cf. Hays, 1994, ch. 18, and Ratcliff and Childers, 2015, p. 244). Heathcote et al. (2002) proposed a quantile based ML estimation method, reporting to deliver results similar to those obtained with the *G*^2^ statistic.

### 1.2. Previous Studies Comparing Estimation Methods

While numerous studies apply the DM, only few compare the various estimation methods.

Ratcliff and Tuerlinckx (2002) used predefined parameter sets for their simulation reflecting a 2-AFC experiment using four levels of stimulus difficulty. Correspondingly, they varied the drift rate but fixed other parameters either to a single or two different values. They compared the ML, the CS, and a WLS method, finding (amongst other things) ML to outperform the other contestants, but failing in the presence of response time contaminants.

Van Ravanzwaaij and Oberauer (2009) simulated data for a 2-AFC experiment that manipulated stimulus-response compatibility (with a presumed effect on drift rate ν) and speed-accuracy settings (with a presumed effect on threshold *a*) while fixing *z* = *a*/2. They considered the EZ method, the K-S method as implemented in fast-dm, and the DMAT routine, which applies an algorithm comparable to the ML method. They found, in general, high correlations of the parameter estimates and the true parameters, somewhat decreasing when changing from 800 trials to random samples of 80. The EZ method showed some bias in their study and the K-S method of fast-dm showed even more bias for ν. Generally, they found bias to increase with the size of the respective parameters. However, they concluded that “EZ did consistently better than fast-dm and DMAT” (p. 471). Arnold et al. (2015) compared the same three routines in a parameter validation study using experimental recognition-memory data and found results to differ between the methods. However, their main focus was on parameter validity, i.e., how experimental conditions were adequately reflected in the parameter estimates. On that score, they stated “that the measures derived via parameter estimation—at least as estimated with the fast-dm method—are not entirely ‘process pure'” (p. 896). They further noted that “[a]ll manipulations [of experimental conditions] had some side effects on other parameters as well,” but that “in every case the effect sizes were considerably larger for the target parameters than for the side effects” (ibid.).

Wiecki et al. (2013) compared their HDDM program (in a hierarchical and a non-hierarchical variant) to the CS and the ML method, finding the Bayesian method superior. In this study, the CS method performed worst compared to the other ones, especially regarding the threshold separation *a* and the non-decision time *T*ER.

Ratcliff and Childers (2015) compared the ML and the CS method (using a “home-grown fitting program”; p. 245), and the DMAT routine, fast-dm with the K-S option, HDDM, and the EZ method. The specific simulated parameter sets were based on their estimates from studies of numerosity discrimination and lexical decision. They found high correlations of estimated with true parameter values (>0.9 for 1,000 trials, dropping considerably when disposing of 100 or 40 trials). Especially HDDM and DMAT suffered from reducing the number of trials. Generally, they found the ML, CS, and K-S “slightly superior to the EZ method” (p. 257).

Lerche et al. (2016) compared the three fast-dm methods (ML, K-S, and CS), the HDDM program, and the EZ method in a simulation scenario with crossed-out parameters. Generally, they found all methods but CS to recover the original parameters well, however limited for designs involving variability parameters. Moreover, the CS method also showed bias for designs with <200–500 trials. The ML and the HDDM (Bayes) method showed overestimation of *a* and underestimation of *T*ER in designs not involving the variability parameters. As regards estimation precision (evaluated with the squared difference of true value and parameter estimate) the HDDM performed best and CS worst. EZ performed well in recovering ν. The K-S and the EZ method proved most robust when outliers were present.

In a recent competition of Dutilh et al. (2018), expert teams were invited to model the condition differences in 14 two-condition data sets, each reflecting a random dot motion task. In particular, they were asked to provide a model that represents the condition differences in terms of processing ease (drift rate), response caution (boundary separation), a priori bias, and non-decision time, while being unaware of the true model and manipulations used to generate the data. Overall, 56 inferences could be made about the true underlying effects. Among the modelling approaches from 17 expert teams, EZ2 performed best predicting 84 % of the underlying effects correctly.

### 1.3. Research Questions

While for many statistical models one or a few parameter estimation methods are widely considered “canonical,” the overview has shown that a fairly broad variety of methods popularize the application of the DM in experimental psychology. However, currently available method comparison studies are remarkably discordant in their evaluations. Hence, it is still an open question, which routine provides the most convincing parameter estimates under which conditions. In the present simulation scenario, we therefore compare five principles used to determine DM model parameters, closed-form EZ, Bayesian approach, ML, CS, and Kolmogorov-Smirnov.

Lerche et al. (2016) argue that method comparisons across different software solutions could be restricted by software-specific issues, which applies in our case to the ML method, which is implemented in fast-dm and in R. Moreover, there are several ML optimization principles available for obtaining ML estimates (most importantly Nelder-Mead and BFGS), which will also be taken into consideration. Importantly, we only consider programs that are freely available including their source code.

Regarding the model parameters, we will examine the four main parameters *a*, *z*, *T*ER, and ν, but neither the three variability parameters nor response time contaminants (for a recent treatment of best approaches to obtain stable estimates of the variability parameters see Boehm et al., 2018).

## 2. Study Design and Methods

The present study is based on a simulation comparing selected algorithms (see Table 1) with respect to their parameter recovery in terms of bias and root mean squared error (RMSE) of the estimates as well as the run time of each method. The study design follows a grid search across selected true values of each of the four parameters (see Table 2). The rationale for this design is that we do not want to restrict our simulation to the results of studies, which focus on specific experimental conditions, but to cover a wider range of possible outcomes.

**Table 1:** Evaluation methods, abbreviations, and algorithms.

**No**.	**Routine**	**Abbrev**.	**Estimation method**
1	The EZ method implemented in R	R-EZ	Closed form
2	The Bayes method using the rjags/jwm routines	R-BY	Bayesian
3	The R optimize() function using the N-M algorithm	R-NM	Maximum likelihood
4	The R optimize() function using the BFGS algorithm	R-BF	Maximum likelihood
5	The R nlm() function	R-NL	Maximum likelihood
6	The fast-dm ML method	F-ML	Maximum likelihood
7	The fast-dm CS method	F-CS	Chi-square
8	The fast-dm KS method	F-KS	Kolmogorov-Smirnov

**Table 2:** Parameters used in the simulation routine.

**Par**.	**True values**	**Start. Val**.	**Prior Dist**.
*a*	0.5, 1.0, 1.5, 2.0	*U*(0.2, 0.8)	*U*(0.0001, 3)
*z*	0.2, 0.5, 0.8	*U*(0.2, 0.8)	*U*(0.002, 0.998)
*T*ER	0.1, 0.3, 0.5	0.001	*U*(0, 1)
ν	−1.0, −0.5, 0, 0.5, 1.0w	*U*(0.2, 0.8)	*U*(0, 1)
*n*	50, 100, 400		

### 2.1. Programs and Algorithms

The eight estimation methods were applied with four different programs: A direct implementation of the EZ method (see section 2.1.1), the fast-dm program (Voss and Voss, 2007), the RWiener package (Wabersich, 2013) using the R optimizers optimize() and nlm(), and the RJAGS package (Plummer, 2003) using the rwiener module (Wabersich and Vanderkerckhove, 2014). Table 1 gives an overview and introduces the abbreviations used throughout the text.

#### 2.1.1. The EZ Method

We used the R implementation of the algorithm as given in Wagenmakers et al. (2007), which employs a value of 0.1 for the standard deviation of the drift standard deviation *s*. Therefore, the obtained parameter estimates have to be multiplied by the factor 10 in order to become comparable to the estimates of the other routines. The extended version EZ2 (Grasman et al., 2009) has not been applied, because this algorithm requires at least two experimental conditions, a setting not considered in the present simulation study.

#### 2.1.2. The Fast-DM Program

We used the program fast-dm (Voss and Voss, 2007), a free software published under the GNU General Public Licence and written in C++. It is a stand-alone program for the MS Windows® operating system (fast-dm.exe) taking a data file (experiment.dat) containing the responses, the respective response times and (if necessary) the experimental condition. A control file (experiment.ctl) has to be provided containing relevant information on program execution (e.g., which parameters are to be estimated, which method is to be applied, what is the data format, or logging details). The output is written to a file (experiment.out). Further program details can be obtained from Voss et al. (2015).

fast-dm allows for estimating all seven model parameters. As we focus on the four main parameters in the present study, the three variability parameters were fixed at a value of zero.

The program supports the CS, the K-S, and the ML estimation methods. The CS-statistic is calculated as described in section 1.1.2, the K-S method uses the “super-curve” as described in section 1.1.3, and the ML method employs the Nelder-Mead Simplex search. The program provides an option allowing to control the precision of the parameter estimates defaulting to a value of 3. In the present study, a value of 5 has been chosen in order to minimize numerical inaccuracies. The fast-dm program uses *s* = 1 for the drift standard deviation.

The fast-DM algorithm has been implemented in the rtdists package (Singmann et al., 2018, listing Andreas and Jochen Voss, the authors of fast-DM, as contributors in the package documentation). We, therefore, did not include rtdists in this simulation, for it does not constitute an estimation method of its own.

#### 2.1.3. The RWiener Package

Turning to an R-based solution, we used the RWiener package (Wabersich and Vandekerckhove, 2014). Basically, this program provides a set of functions allowing for calculating the density function (2). The package contains 4 R-style distribution functions, dwiener(), pwiener(), qwiener(), and rwiener(), representing the density function *P*(*X* = *x*), the cumulative distribution function *P*(*X* ≤ *x*), the quantile function *F*^−1^(*X*), and the random draw function, each with respect to the response time distribution as denoted in Equation (2).

Moreover, the package comprises the likelihood functions wiener_likelihood() and wiener_deviance() (the latter calling the former and multiplying the result by −2). These two functions yield (minus two times) the log-likelihood of a data.frame (containing responses and response times), given the four main parameters of the model, *a*, *z*, *T*ER, and ν. Furthermore, the package also comprises two goodness-of-fit functions, wiener_aic() and wiener_bic(), and the wiener_plot() function for drawing the typical DM plot (cf. Figure 1).

The wiener_deviance() function is the key to parameter estimation. It is handed over to an optimizer, which seeks the parameters minimizing the deviance measure for a given data set (i.e., an observed or a simulated data.frame) given a set of starting values for the parameters. R provides for several such optimizers, each offering several options for fine-tuning the estimation process. We used the optim() (with both the Nelder-Mead and the BFGS-method) and the nlm() optimizer.

#### 2.1.4. The RJAGS Package and the Wiener Patch

The free software JAGS (Just Another Gibbs Sampler; Plummer, 2003) is a universal tool for performing Bayes analyses using Markov Chain Monte Carlo sampling (MCMC; see Gelman et al., 2014, ch. 11 or Kaplan, 2014, ch. 4 for a detailed introduction and further references). In short, MCMC allows for drawing samples from the joint posterior parameter distribution of a Bayes model. A key feature of MCMC is that the conditional distribution of a sample *X*^(*s*)^ depends only on the previous sample *X*^(*s*−1)^, but not on “older” samples, drawn before *s* − 1. The transition from sample *X*^(*s*)^ to sample *X*^(*s*+1)^ is described by a transition matrix. This fairly general principle requires specification for the respective problem at hand, which can be accomplished with the Gibbs sampler.

Basically, a Gibbs sampler draws from the conditional distribution of one variable (here: parameter posterior) given the others. This drawing is performed in turn for all parameters thus arriving at a new sample of the seeked joint distribution. The Gibbs principle can be written as a special case of the more general Metropolis-Hastings algorithm (Monahan, 2001).

One standard program to run a Bayesian analysis using the Gibbs sampler is BUGS (Bayesian inference Using Gibbs Sampling; Lunn et al., 2012, 2009), a command line tool. It has been developed further to WinBUGS, which provides a graphical user interface. BUGS is at the same time the name of the language used to define the model in a batch script. Alternatively, one may choose to use JAGS, which is written in C++ and runs in a console. It uses the BUGS language and it is intended to constitute a free alternative to the WinBUGS program (Plummer, 2003, p. 2). We used the rjags package (Plummer, 2019), which is a wrapper to the JAGS program, allowing to control a JAGS run from within the R environment.

One advantage of the rjags solution is that it supports the module rwiener (Wabersich and Vanderkerckhove, 2014, or JWM, JAGS Wiener Module). It provides definitions tailored to perform a Bayesian analysis for the DM by making the dwiener function of the RWiener package available to JAGS.

### 2.2. The Simulation Routine

The present simulation study has been realized with R (R Core Team, 2019). In a set of nested loops, all parameter combinations (see next Section) were used to generate random samples. Table 1 shows the routines used for evaluation.

Data sets were generated with the rwiener() function from the RWiener package, which uses the exact, rejection-based algorithm for data simulation (cf. Tuerlinckx et al., 2001). To avoid peculiarities caused by the data generation, we additionally checked the RT-distributions and accuracy rates with the R-package rtdists (Singmann et al., 2018), which covers the fast-dm algorithm. No divergences were found (we want to thank one anonymous reviewer for reminding us of such a check).

The fast-dm program was called from within R using the system() function generating the required control file (experiment.ctl) with the write() function and reading the fast-dm results with the read.table() function of R.

#### 2.2.1. Preparative Steps: Starting Values, Multiple Maxima, and Instable Estimates

In a set of pilot runs of the simulation study, we found an inacceptably high number of missing results when using the optimize-function of R. Both the Nelder-Mead and the BFGS methods failed to deliver a valid result in up to half of the estimation runs. Ancillary simulation runs revealed that failures occurred in those cases, in which the starting value of the *T*ER-parameter (*T*ER^(0)^) was larger than the true parameter, which determines the minimum response time realized. Because the response time density vanishes for *t* < *T*ER, such a case would not constitute a valid measurement. As a consequence, the starting value *T*ER^(0)^ was fixed to 0.001 for all designs. The starting values of the other three parameters did not show any systematic tendencies to prevent the algorithm from starting the iterations. The nlm-routine was not affected and yielded valid estimates independently of the starting values chosen.

Moreover, estimation iterations stopped with the BFGS-methods issuing an error encountering a “Non-finite finite-difference value”. This problem could be overcome by reducing the step-size from (default) 1e-3 to 1e-4. Finally, stops were encountered in cases, in which the starting value for *a* was chosen too small (below ~0.1, independently of the true value of *a*).

Another set of preparative simulations was undertaken to find out, whether multiple maxima existed. For this purpose, 100 data sets were generated randomly with true parameters randomly drawn: *a* ~ *U*(1, 3), *z* ~ *U*(0.2, 0.8), *t* ~ *U*(0.01, 3), and ν ~ *N*(0, 1). Each data set was evaluated 20 times along with a new set of starting values from the same distributions as the true parameters. Generally, all estimates from one data set yielded estimates equal up to the third decimal place. However, some data sets showed exceptionally high variability in their estimates. These could be identified as special cases, realizing only one kind of response (i.e., only “upper” or only “lower”) as a result of extreme parameter combinations, like high bias *z* along with large values of the diffusion rate ν. To avoid such peculiarities, the final simulation limited these two parameters to 0.2 ≤ *z* ≤ 0.8 and −1 ≤ ν ≤ + 1. With this choice, we wanted the starting point parameter *z* to cover almost the entire possible range—if it was outside the chosen interval, the decision would be virtually determined (i.e., P(+) would approach 0 or 1, especially in cases, in which ν points in the same direction, as can be easily verified with the DMV (Alexandrowicz, 2020). If one border is not crossed at all, the corresponding distance between starting point and boundary is not defined (cf. Lerche et al., 2016). Therefore, Lerche et al. excluded problematic data sets with fewer than 4 % of crossings at one of the boundaries. In other simulations, the problem was circumvented by using specific parameter sets and constraining other parameters but not drift rate (Ratcliff and Tuerlinckx, 2002; Ratcliff and Childers, 2015; van Ravanzwaaij and Oberauer, 2009). We decided to constrain drift rate, in order to contribute to the overall results on the accuracy of the estimation methods. Additional preliminary simulations revealed that for the parameter space in Table 2, on average only 9 % of data sets were problematic. By comparison, if we had included |*v*| = 2, more than a third of the data sets (37 %) and for |*v*| = 3 more than half of the data sets (51 %) would result in estimation problems for certain parameter constellations. We considered such a systematic loss substantial and consequential for the performance comparison of the eight methods as listed in Table 1. At the same time, the interval of the drift parameter ν covers values distinctly different from zero so that systematic effects are likely to be detected as well.

#### 2.2.2. Simulation Parameters and Analysis Settings

Column 2 of Table 2 shows the parameter values used in the simulation. We generated five data sets for each of the 4 × 3 × 3 × 5 × 3 = 540 combinations of true parameters. For each of these combinations, three parameter estimation runs were performed using different starting value vectors drawn from the distributions given in Table 2. This procedure resulted in a total of 8,100 estimation runs for each of the methods taken into consideration. Note that in Table 2 and thereafter, *n* will denote the number of trials.

The maximum number of iterations for the R optimizer was set to 5,000. For the Bayesian analysis, three chains of length 500, each, were sampled using the starting values *a* = 1, *T*ER^(0)^ = 0.001, ν = 0.5 for chain 1, *a* = 2, *T*ER^(0)^ = 0.001, ν = −0.5 for chain 2, and *a* = 0, *T*ER^(0)^ = 0.001, ν = 0 for chain 3. To obtain a point estimate for each parameter, the three chains were concatenated and the EAP estimator was applied. The number of samples chosen is lower than in practical applications of the Bayesian method, for three reasons: first, no contaminants or outliers are added, second, we estimate only four parameters, and third, we only consider one single “experimental condition.”

## 3. Results

The present simulation considered eight algorithms for 180 different parameter combinations, each applied to five samples of 50, 100, and 400 trials and replicating the parameter estimation with three randomly drawn starting value sets. The resulting output of 8,100 estimates × eight methods is therefore remarkably large. Hence, crucial elements are reported here, more details are provided in the Supplementary Material.

### 3.1. Sample Statistics

Table 1 in the Supplementary Material shows the proportions of upper and lower threshold passes, the average response times and the average standard deviations of the response times realized in the samples for each of the 180 designs.

### 3.2. Checking Bayes: Estimation Failures and Convergence

From the 8,100 runs using the Bayesian method, 162 (2 %) failed to deliver a result. No parameter combination failed entirely (i.e., at least one of the five different data sets did allow for an estimation). However, if a failure occurred for a data set, then none of the replications with newly drawn starting values succeeded. Hence, failures occurred due to features of the individual data sets, but they are not specific to certain parameter combinations. Table 2 in the Supplementary Material shows the true parameters and the descriptive statistics for those 54 data sets failing to deliver an estimate in all three starting value replications. Probably most striking is the fact that all omissions occurred in samples with *a* = 2. Moreover, omission occurred predominantly for samples of 400 trials (69 %).

Next, we turn to the convergence behavior of the MCM-chains. The sheer number would not allow for detailed reporting here (4*a* × 3*z* × 3*T*ER × 5ν × 3*n* × 5 datasets × 3 replications = 8,100 estimation runs), however, interested readers may obtain a 90 MB pdf (of size 5 m × 38 cm; don't print!) containing all plots for all parameters upon request. Both authors and one colleague (NV) have checked the plots not detecting any conspicuous features.

Moreover, the Potential Scale Reduction Factor (PTSR; Gelman and Rubin, 1992) allows for a descriptive assessment regarding the convergence of the three chains per parameter by comparing the variance within the chains with their between-variance in an ANOVA-like fashion. A value of 1 denotes optimal convergence and values substantially larger than 1 indicate that the chains may not be indistinguishable to a sufficient extent. The index was computed with the gelman.diag() function of the coda package (Plummer et al., 2006). Table 3 shows descriptive statistics of this measure for each parameter computed across all successful runs.

**Table 3:** Descriptive statistics of the Potential Scale Reduction Factor.

		**Min**	**Mean**	**Median**	**Max**	***SD***
*a*	est	0.998	1.014	1.009	1.260	0.016
*a*	uci	0.998	1.044	1.028	1.711	0.050
*z*	est	0.998	1.023	1.015	1.352	0.027
*z*	uci	0.998	1.071	1.047	1.934	0.082
*T*ER	est	0.998	1.024	1.016	1.256	0.026
*T*ER	uci	0.998	1.068	1.046	1.834	0.074
ν	est	0.998	1.012	1.009	1.097	0.012
ν	uci	0.998	1.041	1.028	1.304	0.041

*est, point estimate; uci, upper 95 % confidence interval limit*.

The largest value of all chains for all parameters was 1.9 for the upper 95 % confidence limit of *T*ER. The largest mean was 1.07 and no value exceeded 1.35. All upper confidence interval limits were below 2. Figure 1 in section 2 of the Supplementary Material shows the histograms of the PSRF statistic of the four parameters. Again, the distributions do not show outliers indicating convergence problems. We therefore conclude from both analyses that with the present settings, the Bayesian estimation method converged successfully in all instances.

### 3.3. Sample Statistics and Parameter Estimates

**Figure 3:**
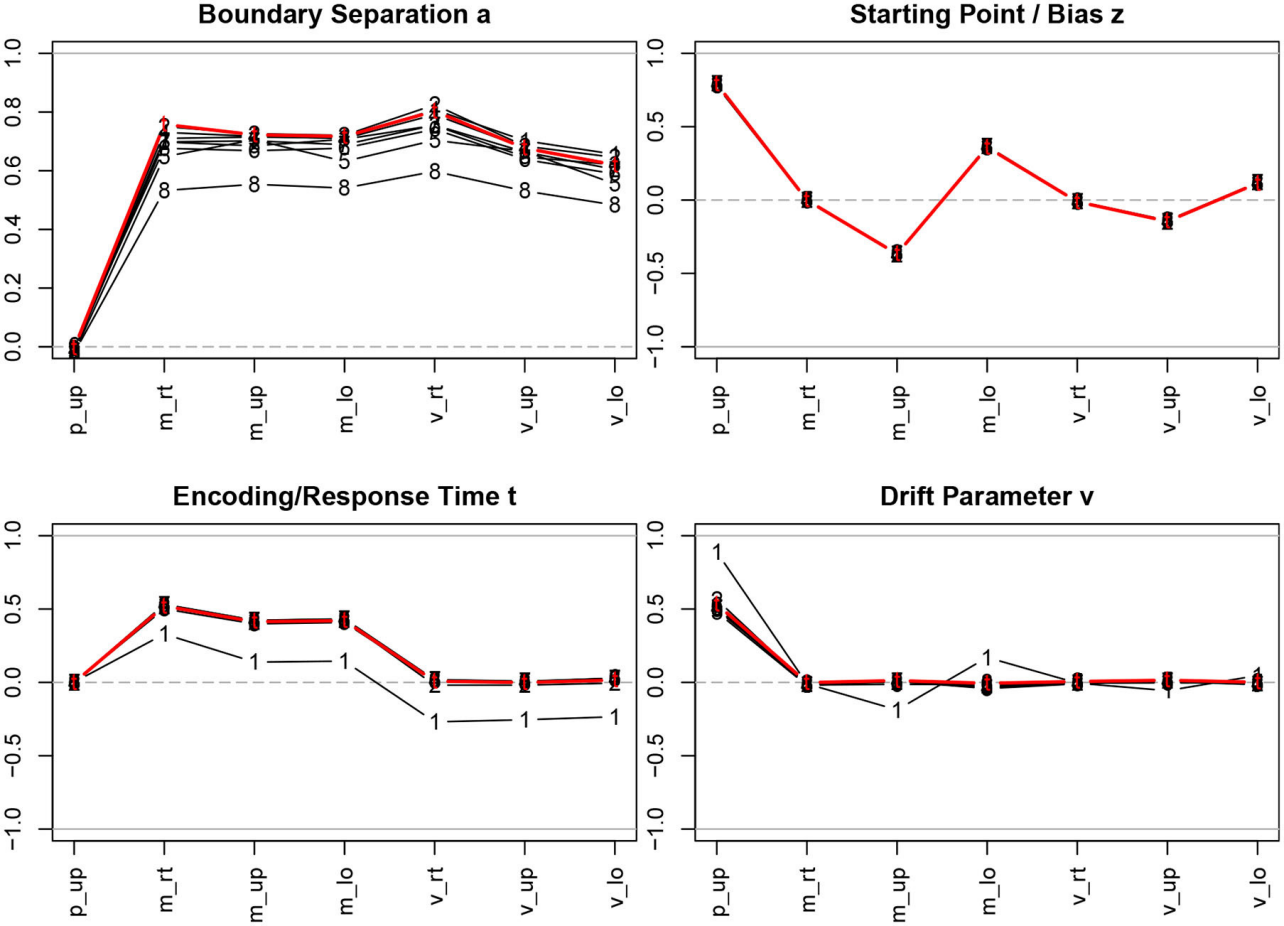
Correlation coefficients of descriptive statistics with the parameter estimates. The bold (red) lines indicate the correlations with the true parameter values; the estimation methods are indicated by numbers, 1 = R-EZ, 2 = R-BY, 3 = R-BF, 4 = R-NL, 5 = R-NM, 6 = F-ML, 7 = F-KS, 8 = F-CS; p_up, proportion of upper boundary crossings; m_rt, mean response time; m_up, mean response time for upper boundary crossings; m_lo, mean response time for lower boundary crossings; v_rt, variance of response time; v_up, variance of response time for upper boundary crossings; v_lo, variance of response time for lower boundary crossings.

Figure 3 shows the correlation coefficients of descriptive statistics of the samples with for the true and the estimated parameters.

Considering the true parameter values (bold red lines in Figure 3), we find the well-known relations, i.e., *a* is strongly related to the response time means and variances, *z* depends primarily on the proportion of upper boundary crossings and, to a fairly lesser extent, on the response time means, *T*ER depends on the minimum response time observed, resulting in a medium size correlation with the response time means, and, finally, ν is associated with the proportion of upper/lower boundary crossings.

The interesting information from Figure 3 is to which extent the correlation coefficients of the true parameters with the descriptive measures are recovered when we turn to the parameter estimation methods. Generally, we find exceptionally high agreement across methods, with a few exceptions: *(i)* The estimates of the boundary separation parameter *a* exhibit a slight disagreement, yet not to be considered substantial. Especially the CS method (code 8 in Figure 3; top left) yields somewhat lower correlations compared to the other methods. *(ii)* The the EZ estimates of *T*ER (code 1 in Figure 3; bottom left) yield lower correlations across all descriptive statistics. *(iii)* The EZ estimates of the drift parameter ν show higher correlations (in absolute value) with the proportion of upper boundary crossings and (to a much lesser extent) with the two mean response times.

Tables 3a–d in section 3 of the Supplementary Material report the values used for Figure 3 and the respective values after splitting by number of trials. However, no marked discrepancies to the over-all results are to be observed, except for the variations in the boundary separation *a*, which were more pronounced when only using the 50 trials data sets and becoming increasingly more homogeneous with growing number of trials.

### 3.4. Distributions of the Parameter Estimates

Figures 2a–d in section 4 of the Supplementary Material show the distributions of the estimates by method, trial number, and parameter. For the boundary separation parameter *a* (Supplementary Figure 2a) we find largely good parameter recovery, except, maybe, for the EZ, the K-S, and the CS method, especially, when only 50 trials are available. The starting point *z* (Supplementary Figure 2b) also recovers fairly well, again with slight deficiencies occurring with K-S and CS for small samples. The recovery of the encoding and response time *T*ER (Supplementary Figure 2c) is exceptionally good with all methods, and, interestingly, only shows a very small increase of variability for small samples. However, there is one irritating finding: Some of the *T*ER estimates obtained with EZ are negative, which is clearly a violation of the model assumptions. Finally, the recovery of the drift parameter ν (Supplementary Figure 2d) performs worst: All methods show a comparably large variability (especially when looking at the EZ method, top left plot). Interestingly, the Bayesian estimates (top right plot in Supplementary Figure 2d) also exhibit a slight bias, in that the extreme values (i.e., ν = ±1) seem to be slightly biased toward zero, a tendency that decreases with increasing sample size.

#### 3.4.1. Special Issue I: Invalid *T*ER Estimates

The EZ method yielded 1,002 invalid estimates of *T*ER < 0. To find out, whether these are related to other parameters, we counted their occurrences separately for the levels of *a*, *z*, *T*ER, and ν. Table 4 shows the resulting frequencies and percentages. First of all, and hardly surprising, most of the invalid estimates (79 %) occurred for small values of *T*ER and decreased as *T*ER increased. Moreover, the frequencies of invalid estimates also increased in cases, in which *z* departs from 0.5 (in both directions) and in which *a* increased. The drift parameter ν seems not to have a strong association, yet we find a peak around ν = 0 and decreasing frequencies with increasing distance from ν = 0.

**Table 4:** Frequencies and percentages of negative *T*ER estimates by levels of all parameters.

***T*ER**	**Freq**.	**Pct**.		**z**	**Freq**.	**Pct**.
0.1	792	79.0		0.2	507	50.6
0.3	198	19.8		0.5	27	2.7
0.5	12	1.2		0.8	468	46.7
∑	1002	100.0		∑	1002	100.0
**a**	**Freq**.	**Pct**.		**ν**	**Freq**.	**Pct**.
0.5	0	0.0		−1	144	14.4
1	63	6.3		−0.5	219	21.9
1.5	369	36.8		0	246	24.6
2	570	56.9		0.5	225	22.5
				1	168	16.8
∑	1002	100.0		∑	1002	100.0

### 3.5. Correlation Analysis

One way to approach parameter recovery is to look at the correlation coefficients of true and estimated parameters, which will be done in the following section. Thereafter, we also assess the estimates' correlation coefficients across the 8 estimation methods.

#### 3.5.1. True and Estimated Parameters

**Figure 4:**
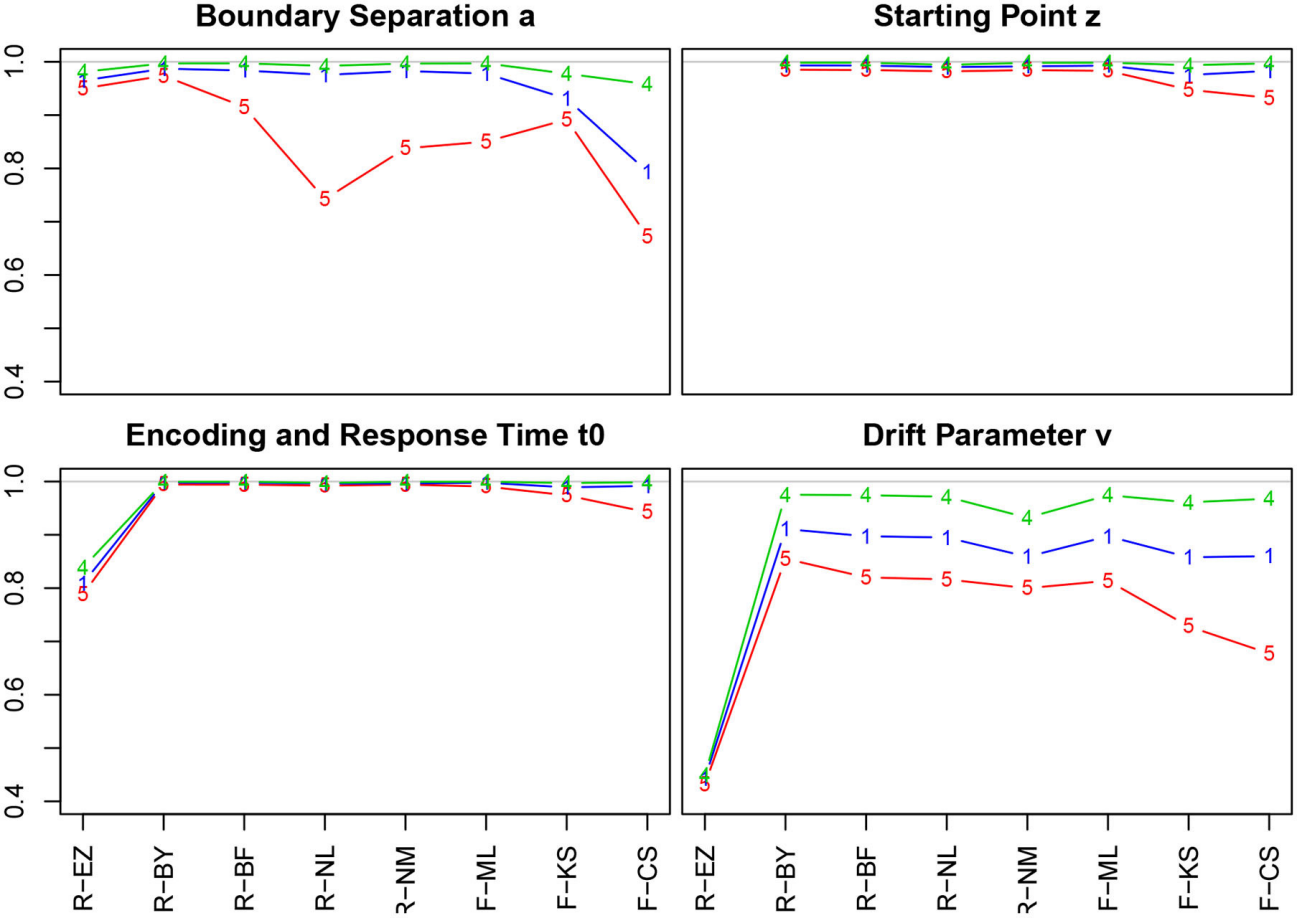
Correlation coefficients of true and estimated parameters by number of trials. red line/symbol “5”: 50 trials; blue line/symbol “1”: 100 trials; green line/symbol “4”: 400 trials; for abbreviations see Figure 3.

Figure 4 shows the correlation coefficients of true and estimated parameters for the 8 methods using the entire sample and split by the number of trials (Table 4 in section 5.1 of the Supplementary Material lists the values).

Generally, we find high correlation coefficients, with a few exceptions. There are four major tendencies: *(i)* Unsurprisingly, the coefficients increase with the number of trials across all methods and parameters—most prominently for the drift parameter ν. *(ii)* Notably, the *T*ER parameter has been recovered best, with almost all values above 0.99. Only the EZ method yielded slightly inferior coefficients. *(iii)* The CS method of fast-dm performed somewhat worse compared to the other iterative methods for all parameters, yet to a varying degree: With decreasing number of trials it decreased most for the boundary separation *a* and drift rate ν, and least for starting point *z* and encoding and response time *T*ER. Also, the KS method performed somewhat poorly for *z*, *T*ER, and ν when 50 trials were available. *(iv)* When disposing of 400 trials, all methods (but EZ) showed correlation coefficients close to 1. *(v)* The Bayes estimates performed exceptionally well for all parameters independent of the number of trials.

The EZ method performed considerably poor in recovering the drift parameter ν (*r* ~ 0.44). This fact is discussed further in section 3.6.5. A slight incursion (*r* = 0.74) appeared for the boundary separation *a* with 50 trials when using the nlm() function of R. However, the value is still satisfying, hence we consider this a spurious result (the more, as in a replication of the simulations, this incursion did not reappear).

#### 3.5.2. Correlations Among Parameter Estimates

Tables 5a–d in section 5.2 of the Supplementary Material show the correlation coefficients across parameters and methods. The large table is split into four sections, each holding the coefficients of one parameter for all methods (5a: *a* vs. the rest; 5b: *z* vs. the rest; 5c: *T*ER vs. the rest; td: ν vs. the rest). The table represents the full matrix, i.e., values are mirrored along the main diagonal. The gray sections indicate correlation coefficients of estimates of the same parameters across methods. Note that no correlation of the EZ estimates of *z* can be obtained, because the EZ routine used here fixes this parameter at a value of 0.5. An asterisk indicates the respective entries in the table. Figures 3a–d in section 5.6 of the Supplementary Material show the respective scatter plots with colors indicating the number of trials.

First of all, we find predominantly high coefficients (0.9 and above, many close to 1) in the diagonal blocks (i.e., *a* with *a*, etc.; grayed cells) and close to zero coefficients in the off-diagonal blocks (i.e., *a* with *z*, etc.; non-grayed cells). However, there are some exceptions to this pattern, all associated with the EZ method: *(i)* The EZ estimates of *T*ER show a slight negative correlation to the *a* estimates of all other methods (all in the vicinity of 0.3). *(ii)* The EZ estimates of ν correlate highly with the *z* estimates of all other methods (all in the vicinity of 0.76). *(iii)* The EZ estimates of ν correlate only moderately with the ν estimates of the other methods (all about 0.49).

Splitting the data according to the number of trials (see Supplementary Material, sections 5.3–5.5, Tables 6a–d for 50 trials, 7a–d for 100 trials, and 8a–d for 400 trials), we find another peculiarity: For the 50 trial data sets, the ν estimates of the K-S and the CS method as applied in the fast-dm program correlate slightly with the *z* estimates of the other methods (except, of course, EZ, which does not allow for calculating these coefficients).

In all cases, we observe that the tendencies (high correlations within the parameter blocks, almost zero correlations for the off-diagonal blocks) become more pronounced with increasing number of trials. Apart from that, we find all anomalies reported before, also (slightly) increasing in size with increasing number of trials.

### 3.6. Parameter Recovery Performance Measures

The bias and the root mean squared error (RMSE) are proper means to summarize parameter recovery, hence we will focus on these two measures in this section. Section 6 in the Supplementary Material provides a detailed breakdown of measures, which we will refer to where appropriate.

#### 3.6.1. The Boundary Separation *a*

**Figure 5:**
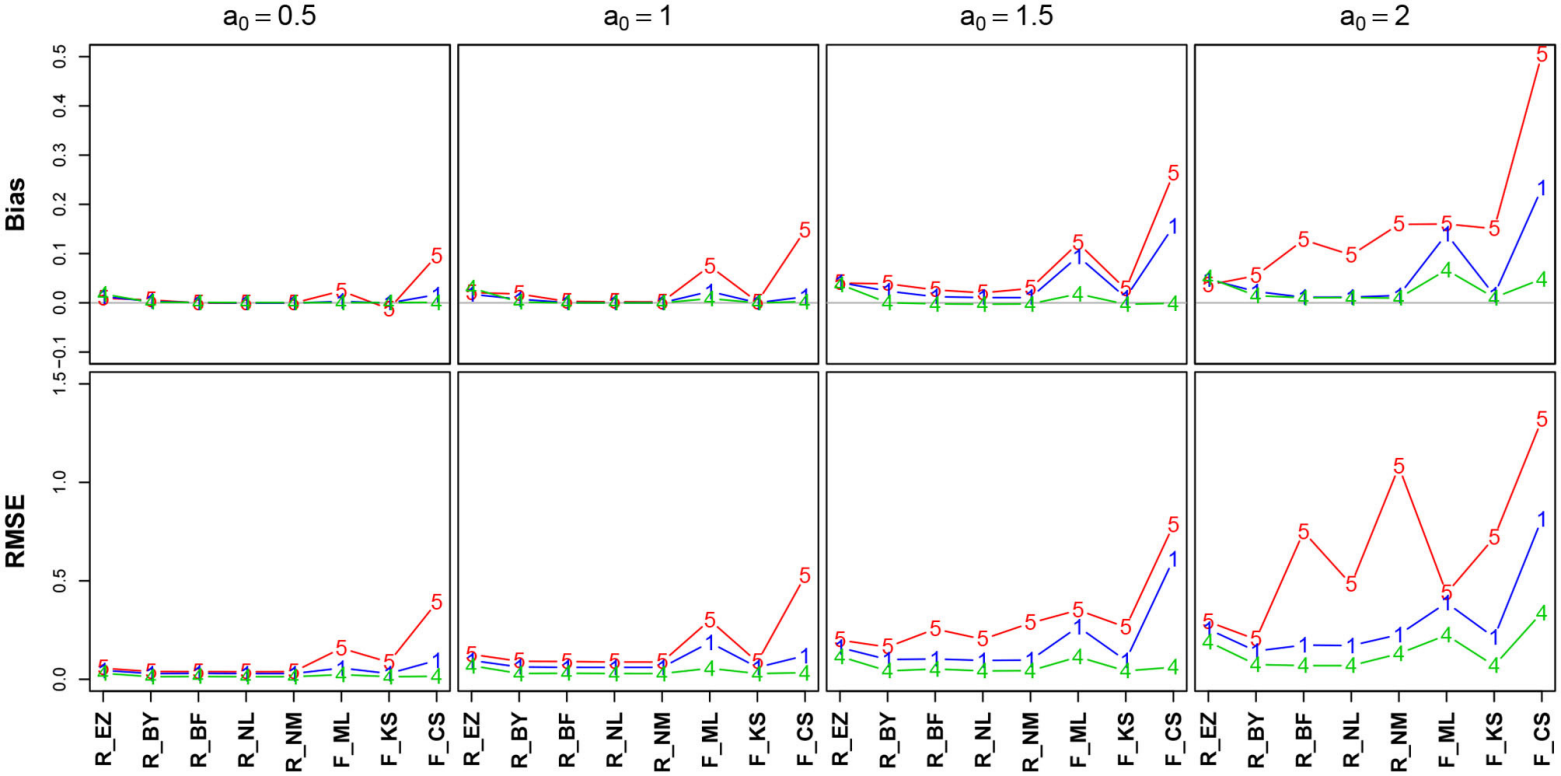
Bias (upper row) and RMSE (lower row) of the boundary separation parameter *a*. red line/symbol “5”: 50 trials; blue line/symbol “1”: 100 trials; green line/symbol “4”: 400 trials; for abbreviations see Figure 3.

Figure 5 shows the bias (upper row) and the RMSE (lower row) of the estimated *a* (Table 9 in the Supplementary Material details all parameter combinations).

Generally, bias and RMSE are comparably small for almost all methods. Only the bias of the CS estimates as provided by fast-dm is somewhat increased compared to the others. For increasing *a*, both bias and RMSE increase, however, even the worst cases seem acceptable with one exception: The CS method delivers estimates, which are considerably biased upwards (up to almost half a unit for *a* = 2 when *n* = 50); this could be considered questionable. In all cases, bias becomes smaller with increasing number of trials. If bias is observable, it is positive, i.e., the boundary separation parameter estimates are larger than the true values (indicated by *a*_0_ in Figure 5). Regarding RMSE, we find increasing values as *a* increases and *n* decreases.

#### 3.6.2. The Starting Point (Bias) *z*

**Figure 6:**
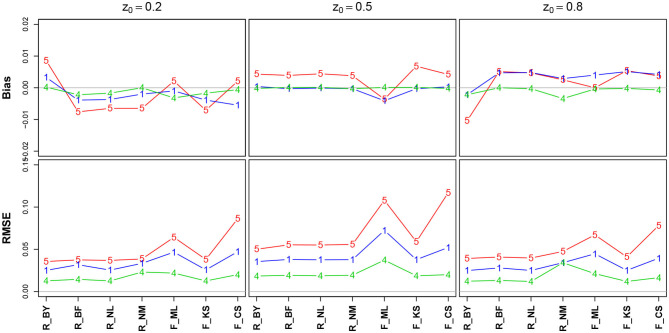
Bias (upper row) and RMSE (lower row) of the starting point parameter *z*. red line/symbol “5”: 50 trials; blue line/symbol “1”: 100 trials; green line/symbol “4”: 400 trials; for abbreviations see Figure 3.

Figure 6 shows the bias and the RMSE for the starting point parameter *z*. Note that the values for the EZ method have been omitted, as it fixes *z* = 0.5 (Table 10 in the Supplementary Material details the values).

There is no substantial bias or increased RMSE observable. The number of trials has hardly an influence, and if so, it appears as expected, i.e., both measures decrease with increasing *n*. Interestingly, the RMSE is (relatively) largest for *z* = 0.5.

#### 3.6.3. The Encoding & Response/Non-decision Time *T*ER

**Figure 7:**
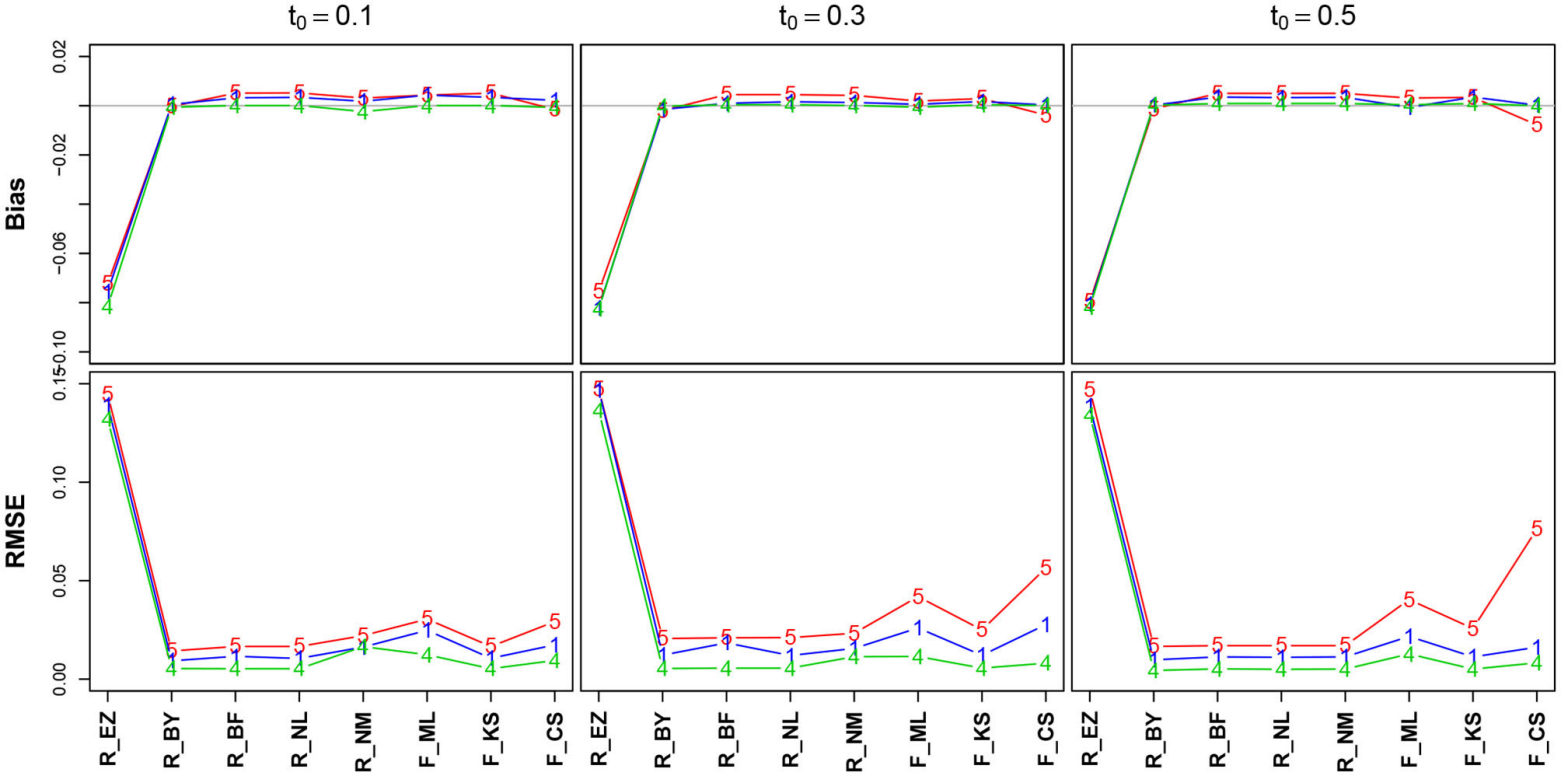
Bias (upper row) and RMSE (lower row) of the encoding and response time *T*ER. red line/symbol “5”: 50 trials; blue line/symbol “1”: 100 trials; green line/symbol “4”: 400 trials; for abbreviations see Figure 3.

Figure 7 shows the bias and the RMSE for *T*ER (Table 11 in the Supplementary Material details the values). Again, we find excellent values regarding bias and RMSE of *T*ER, except for the EZ method, which deviates to a comparatively large extent. In section 3.6.5, we will take a closer look at this result. No substantial effect of the number of trials is visible (but nevertheless, in the lower row the lines pile as expected, yet with hardly a displacement). The estimates obtained by the CS method have minimally increased RMSE for *T*ER = 0.3 and *T*ER = 0.5.

#### 3.6.4. The Drift Parameter ν

**Figure 8:**
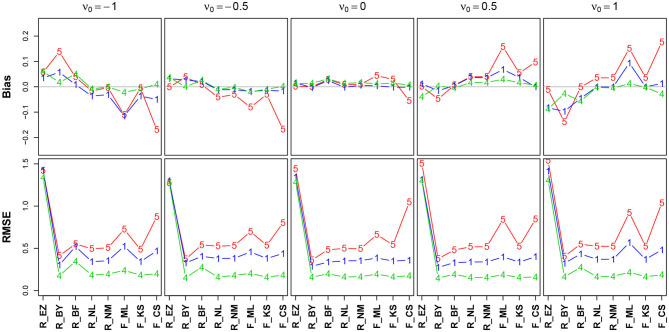
Bias (upper row) and RMSE (lower row) of the drift parameter ν. red line/symbol “5”: 50 trials; blue line/symbol “1”: 100 trials; green line/symbol “4”: 400 trials; for abbreviations see Figure 3.

Figure 8 shows the bias and the RMSE for ν (Table 12 in the Supplementary Material details the values).

The bias (top row) shows an interesting pattern of symmetry: The neutral drift, ν_0_ = 0 (upper row, plot in the middle), is estimated virtually free of bias by all methods (except, maybe, the CS method at *n* = 50). In contrast, the two extreme positions (ν_0_ = −1/ + 1) show a characteristic pattern for *n* = 50 (red lines), yet mirrored: ν_0_ = −1 is underestimated by K-S and CS and (slightly) overestimated by Bayes. Analogously, ν_0_ = + 1 is overestimated by CS and underestimated by Bayes. Interestingly, the ML estimates of fast-dm (which applies the Nelder-Mead search method, see section 2.1.2) have larger bias and RMSE than the respective method applied by the R optimize() function.

The RMSE shows a clear deficiency of the EZ method compared to the other ones (however, see section 3.6.5). It further reveals a clear effect of the number of trials across all levels of ν_0_.

#### 3.6.5. Special Issue II: EZ and the Constant *z*

**Figure 9:**
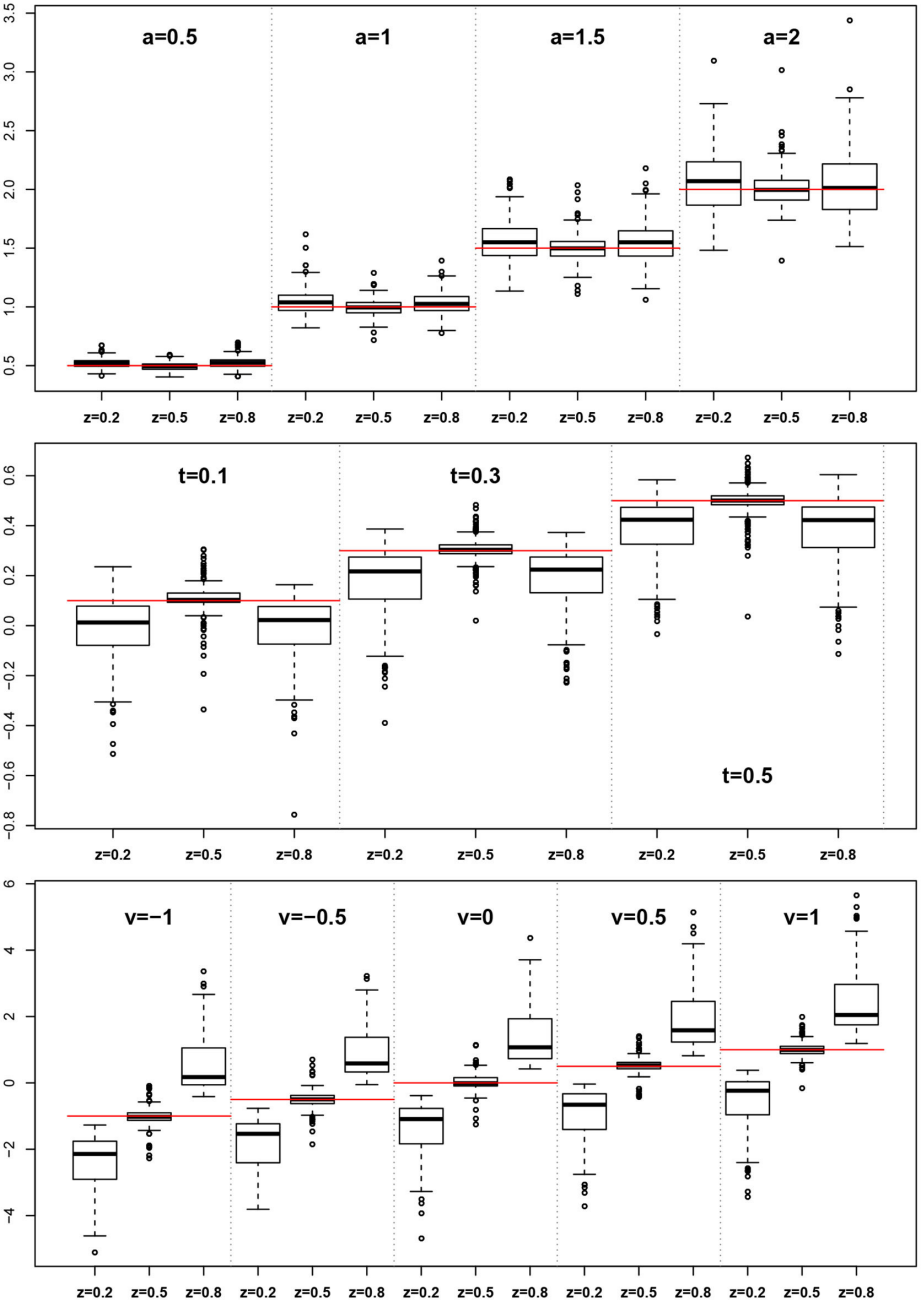
EZ-estimates of *a*, *T*ER, and ν split by levels of *z*. The red horizontal lines indicate the respective true parameter values.

The EZ method as implemented here does not allow for estimating the *z* parameter, rather it assumes *z* = 0.5. This conforms to its true value in one third of the designs, but is wrong in all other cases. At this point, we want to evaluate, whether the simulated violations of the EZ-assumption *z* = 0.5 causes bias in the other estimated parameters. Figure 9 shows boxplots of the distributions of *a*, *T*ER, and ν side for the three levels of *z* (i.e., 0.2, 0.5, and 0.8).

For the boundary separation *a* we find a small to moderate bias upwards. In contrast, *T*ER shows a marked downward bias in all cases, in which *z* ≠ 0.5. Finally, the drift rate ν exhibits a severe bias following *z*, i.e., it is severely underestimated for *z* < 0.5 and severely overestimated for *z* > 0.5.

### 3.7. Interaction Effects

Each of the analyses presented so far considered one factor at a time. As we dispose of a complete crossing of all factors (parameter levels, estimation method, and number of trials), interaction effects can be determined as well. However, this cannot be achieved by inspecting diagrams of all factor levels combinations. Therefore, we used Analysis of Variance (ANOVA) to systematically direct us to possibly interesting interactions due to merely technical reasons. We chose the RMSE as dependent variable, constituting a suitable measure of performance. The critical limit for the type-I-error risk was chosen at 0.05. Significant intercepts will not be considered for they do not contribute to a substantive interpretation.

#### 3.7.1. The Boundary Separation

**Figure 10:**
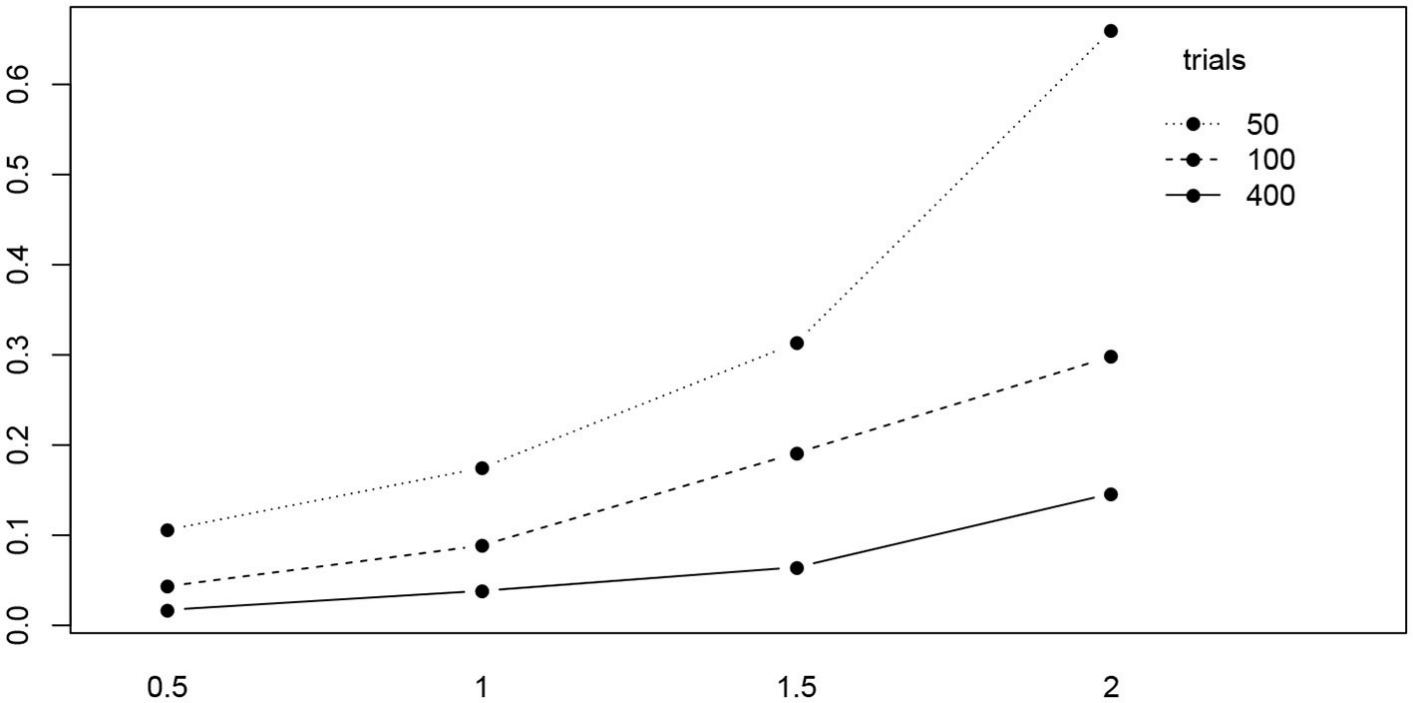
The interaction plot of the RMSE of *a* by true value (horizontal axis) and number of trials (separate lines).

Table 5 shows the ANOVA table of the RMSE by true value, method, and the number of trials. We find a significant main effect of the true values and two significant interaction effects, trials by method and trials by true value. Figure 10 shows the interaction plot of true value by number of trials. We find that for smaller number of trials, the RMSE grows disproportionally, the larger *a*.

**Table 5:** ANOVA of the RMSE of the boundary separation *a* by true value, estimation method, and number of trials.

	**Sum Sq**	**Df**	***F* value**	**Pr (>F)**
(Intercept)	0.09	1	5.63	0.0207
True	1.27	1	77.64	0.0000
Method	0.09	7	0.75	0.6316
Trials	0.02	1	1.08	0.3029
True:method	0.37	7	3.22	0.0055
True:trials	0.28	1	16.93	0.0001
Method:trials	0.05	7	0.41	0.8922
True:method:trials	0.12	7	1.00	0.4371
Residuals	1.05	64		

**Figure 11:**
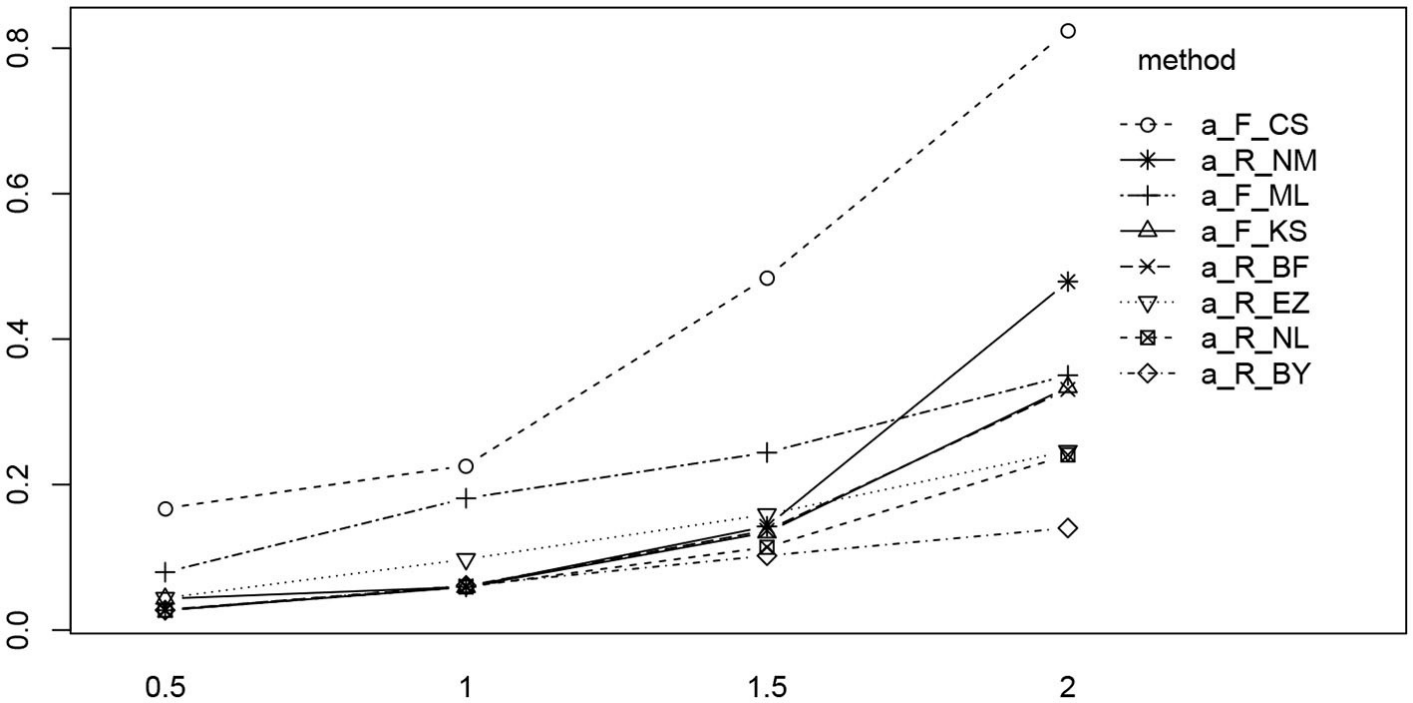
The interaction plot of the RMSE of *a* by true value (horizontal axis) and methods (separate lines). The eight methods were reordered in the legend according to the right-most position in the diagram.

Figure 11 shows the interaction plot of true value by estimation method. Clearly, the RMSE of the CS method grows disproportionally with *a*, and we observe a similar yet weaker effect of the K-S (fast-dm) and the Nelder-Mead method of R. The remaining methods exhibit a fairly linear ascent across the levels of *a*.

#### 3.7.2. The Starting Value/Bias

The ANOVA of the starting value *z* (see Table 6) shows no significant result (apart from the intercept, not posing a substantial interpretation in our design).

**Table 6:** ANOVA of the RMSE of the starting value/bias *z* by true value, estimation method, and number of trials.

	**Sum Sq**	**Df**	***F* value**	**Pr (>F)**
(Intercept)	0.03	1	6.41	0.0154
True	0.00	1	0.00	0.9913
Method	0.02	7	0.47	0.8488
Trials	0.00	1	0.45	0.5042
True:method	0.00	7	0.00	1.0000
True:trials	0.00	1	0.00	0.9976
Method:trials	0.00	7	0.03	1.0000
True:method:trials	0.00	7	0.00	1.0000
Residuals	0.19	40		

#### 3.7.3. The Encoding and Response Time

Table 7 shows the ANOVA table of the RMSE of the non-decision time *T*ER by true value, method, and number of trials.

**Table 7:** ANOVA of the RMSE of the encoding and reaction time *T*ER by true value, estimation method, and number of trials.

	**Sum Sq**	**Df**	***F* value**	**Pr (>F)**
(Intercept)	0.01	1	140.08	0.0000
True	0.00	1	2.27	0.1394
Method	0.01	7	27.13	0.0000
Trials	0.00	1	5.28	0.0269
True:method	0.00	7	1.18	0.3347
True:trials	0.00	1	1.58	0.2158
Method:trials	0.00	7	0.11	0.9971
True:method:trials	0.00	7	0.51	0.8244
Residuals	0.00	40		

**Figure 12:**
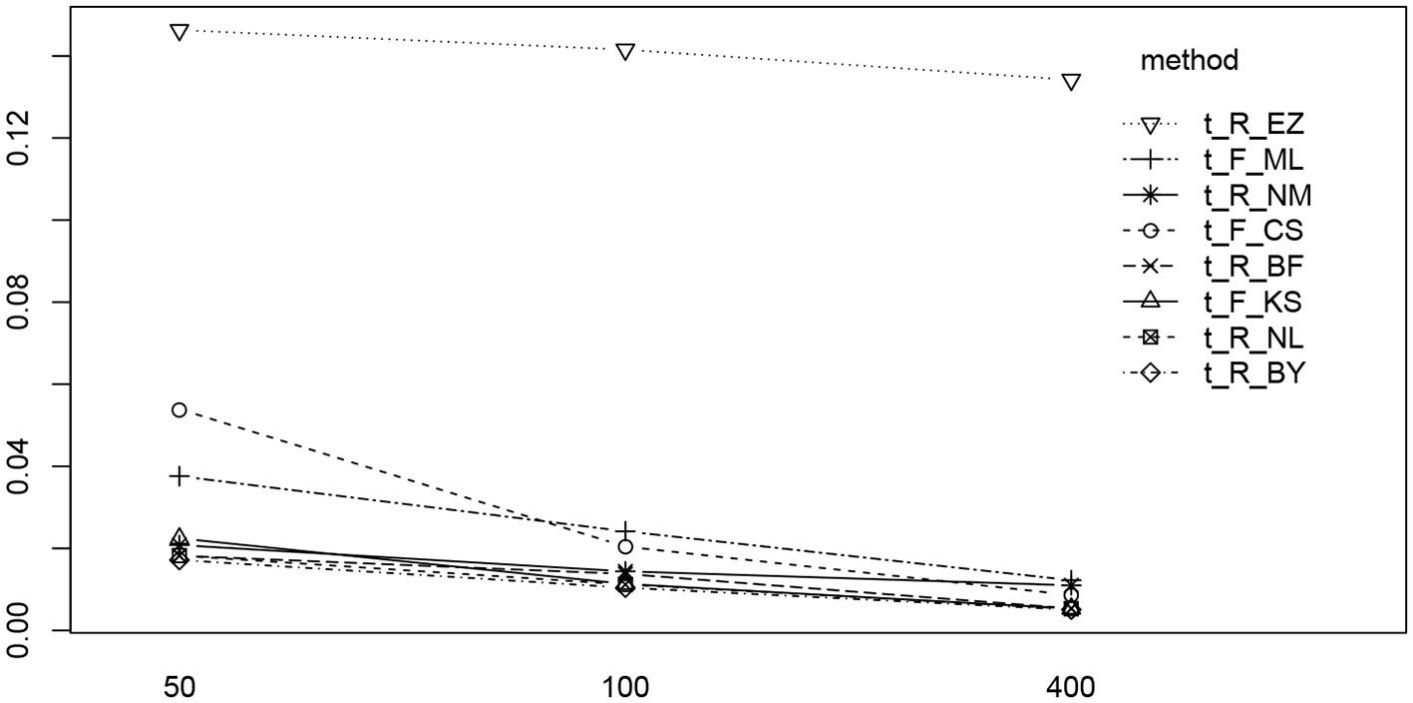
The interaction plot of the RMSE of *T*ER by number of trials (horizontal axis) and method (separate lines).

We find two significant main effects for the estimation method and the number of trials. In Figure 12 we find the reason for the strong method effect, which is obviously due to the high RMSE of the EZ method in contrast to all other estimation methods. The trials effect is comparably small and appears mostly due to the CS and the ML methods of fast-dm. These show a slightly increased RMSE for the 50 trial designs.

#### 3.7.4. The Drift Parameter

The ANOVA of ν (Table 8) reveals three significant effects (plus intercept, again irrelevant), which are the estimation method, the number of trials and the interaction of these two.

**Table 8:** ANOVA of the RMSE of the drift parameter ν by true value, estimation method, and number of trials.

	**Sum Sq**	**Df**	***F* value**	**Pr (>F)**
(Intercept)	22.65	1	2221.17	0.0000
True	0.01	1	1.44	0.2340
Method	4.81	7	67.38	0.0000
Trials	2.31	1	226.51	0.0000
True:method	0.03	7	0.40	0.9002
True:trials	0.02	1	1.50	0.2241
Method:trials	0.46	7	6.44	0.0000
True:method:trials	0.01	7	0.13	0.9957
Residuals	0.90	88		

**Figure 13:**
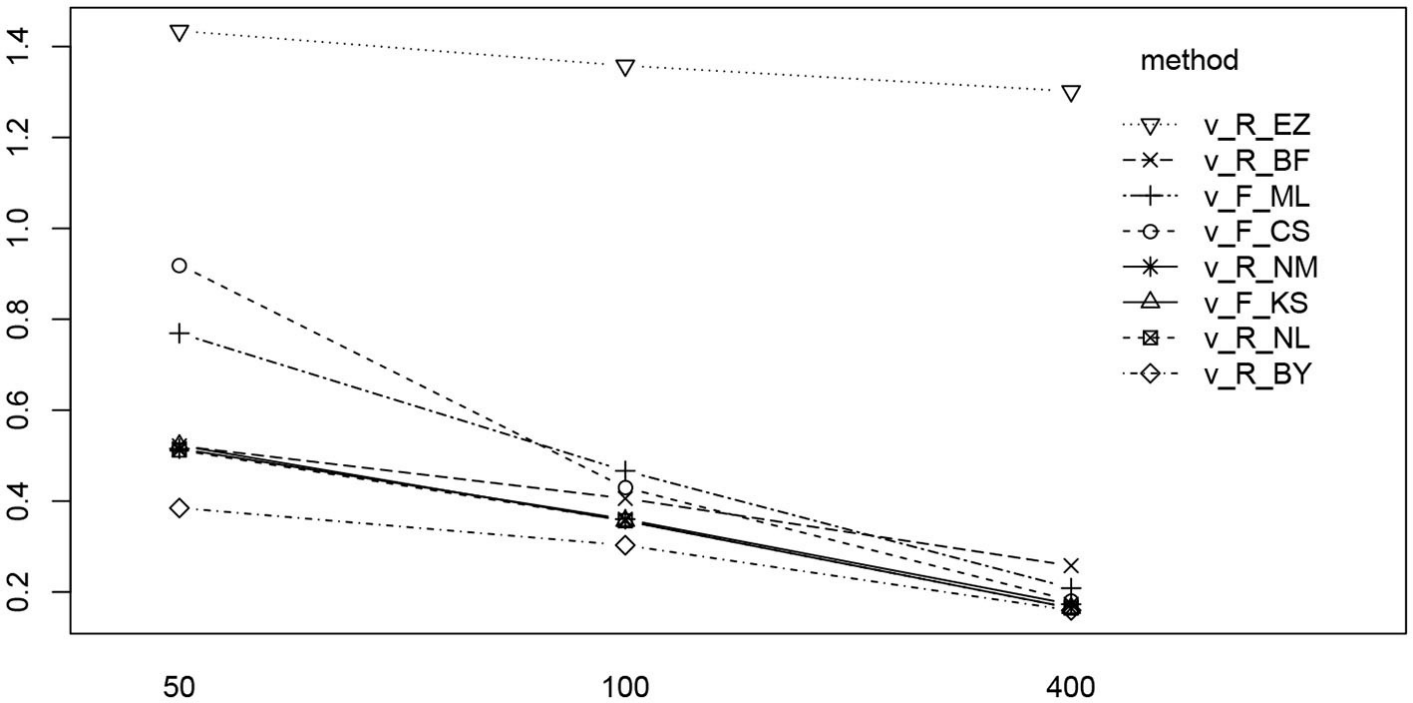
The interaction plot of the RMSE of ν by number of trials (horizontal axis) and method (separate lines).

In Figure 13 we find a similar situation as has been observed for the *T*ER parameter, yet with a more pronounced effect of small number of trials (hence yielding a significant interaction effect as well). Again, the EZ method has a disproportionately high RMSE compared to the other methods and all methods worsen the fewer trials we dispose of. This worsening is most pronounced for the CS and the ML method of fast-dm.

#### 3.7.5. Special Issue III: A ν vs. *a* Interaction

**Figure 14:**
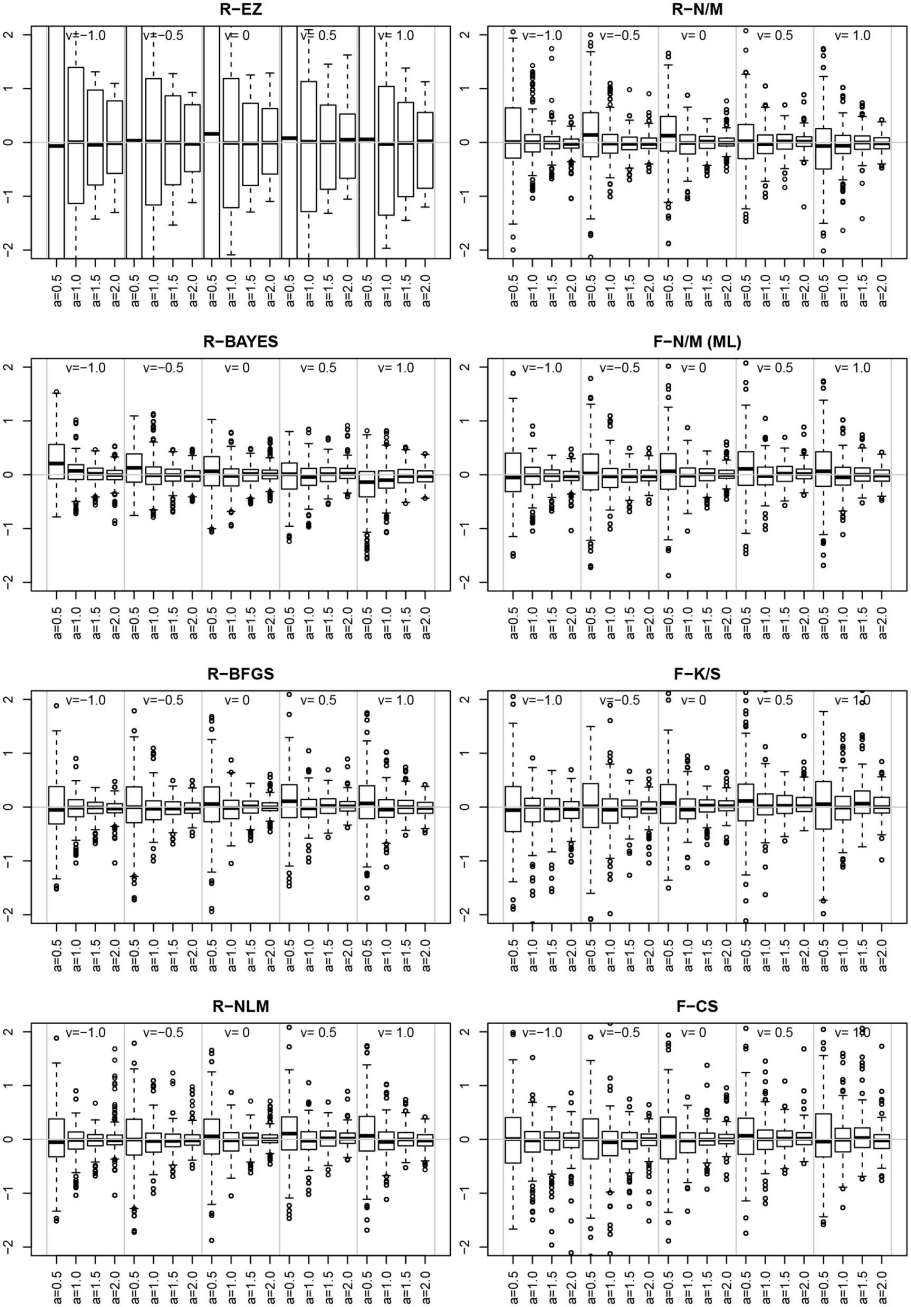
The deviation $\hat{\nu }-\nu$ for the levels of ν (sections separated by vertical gray lines) and the levels of *a* (separate boxplots). To make the eight methods comparable, the vertical axes were scaled uniformly to a range of −2 to 2. As a consequence, the EZ-boxplots outrange the plot limits.

Inspired by the effects reported in section 3.6.5, we further assessed estimates and their deviations from the true value across levels of other parameters, finding the deviation of ν to vary across the levels of *a*. Figure 14 shows the deviation $\hat{\nu }-\nu$ for the chosen levels of ν split by the levels of *a*.

First of all, comparing the 8 methods, the EZ estimates of ν show a strikingly larger variability than the others. Aside of this observation (which has been already reported in section 3.4), we find a clear interaction effect of the variability of the ν estimates across the levels of *a* for all methods: the smaller *a* the larger the variability of ν. Especially if *a* = 0.5 the variability is markedly increased compared to the other levels of *a*.

### 3.8. Run-Time

Table 9 shows the means and standard deviations of the various algorithms' run-times (in seconds) split by number of trials.

**Table 9:** Descriptive statistics of the run-time (in seconds) by algorithm and number of trials.

**Program**	**Alg**	**Trials**	***n***	**Mean**	***SD***
R	EZ	50	2700	0.00	0.00
		100	2700	0.00	0.00
		400	2700	0.00	0.00
R	Bayes	50	2685	6.37	0.29
		100	2664	12.61	0.58
		400	2589	49.71	1.79
R	BFGS	50	2689	0.89	0.27
		100	2686	1.75	0.37
		400	2687	6.94	1.49
R	NLM	50	2700	0.77	0.23
		100	2700	1.51	0.33
		400	2696	5.91	1.33
R	N-M	50	2700	1.32	0.46
		100	2699	2.60	0.88
		400	2698	10.17	3.50
fast-dm	ML	50	2700	0.30	0.50
		100	2700	0.44	0.62
		400	2700	0.69	0.74
fast-dm	K-S	50	2700	6.05	4.11
		100	2700	6.69	4.42
		400	2700	8.00	5.09
fast-dm	CS	50	2700	3.29	2.86
		100	2700	3.50	2.60
		400	2700	3.62	2.46

*Entries 0.00 refer to values (remarkably) smaller than 0.01*.

Clearly, the EZ method is fastest, because it just calculates the estimates in closed form rather than approaching them iteratively. It is also not very surprising that the Bayes method takes considerably longer, owed to its resampling technique. Note that the present parametrization provided for 500 samples per chain, a value that should be chosen larger in applications, given that real data usually will contain contaminants/outliers and involve several experimental conditions and more parameters. The average time consumption was slightly <1 min for a run, a frequently chosen value of 5,000 samples would therefore take 8–10 min per run, which is still acceptable for applications. If one chose to evaluate more than three chains, the run-time would increase accordingly.

The four ML methods were comparably fast, independently of their implementation (fast-dm and R). The K-S and the CS method of fast-dm took somewhat more time for a run, with the latter showing fairly equal durations, independently of the number of trials. This observation can be easily explained by the fact that the CS method performs binning, so that the absolute frequencies per bin increase with the number of trials, but not the number of bins itself.

## 4. Discussion

The present study compared eight open source based estimation methods for the four main parameters of the Ratcliff Diffusion Model. Using a total of 8,100 estimation runs for various parameter level combinations, we found altogether satisfying parameter recovery, with a few exceptions.

### 4.1. Parameter Recovery Across Methods and Implementations

Generally, parameter recovery has turned out well for almost all methods and programs. Some deficiencies occurred, especially for the EZ method. However, this algorithm is not considered an equivalent alternative to the other methods considered here. Rather, it is preferred for quickly obtaining suitable starting values for another method (especially the maximum likelihood based methods). One issue must be pointed out critically: EZ achieved negative estimates for *T*ER in some instances, which is clearly a model violation. These negative estimates could be traced back to small true *T*ER, large *a*, *z* differing considerably from 0.5, and ν close to zero. Such values may be considered extreme, hence the problem seems less important from a practical point of view.

Moreover, we learn from Figure 9 that parameter estimates become severely biased if one of the parameters involved is fixed to an invalid value. This result is in line with Voss et al. (2010), who also found omitted parameters to result in biased estimates of the used parameters. This should raise our concerns insofar, as fixing parameters is frequently applied to foster certain model variants. For example, Arnold et al. (2015) recommend fixing parameters (however, across conditions), if justifiable from a substantive point of view, presuming that invalid restrictions will show up in model fit measures (as was the case in their experiments). Hence, our findings are not in line with van Ravanzwaaij and Oberauer (2009).

The EZ method may be more appropriate in settings, in which the DM's boundaries represent correct and incorrect responses and respondents are unaware of which key is correct and which not and therefore cannot be a priori biased. This might be the case, e.g., in DM applications to the Implicit Association Test as in Klauer et al. (2007). If, in contrast, responses reflect substantive categories, such as “word” and “non-word” in a lexical decision task (Wagenmakers et al., 2008a), or “shoot” and “not shoot” in a first person shooter task (Correll et al., 2015), the presence of a priori bias cannot be excluded. In such cases the EZ method will likely produce highly misleading estimates for the other DM parameters, which might become disadvantageous when using the EZ method as a means to determine suitable starting values. In contrast, in designs, in which the true *z* was indeed 0.5, EZ performed reasonably well.

Aside of EZ, the K-S and the CS methods also showed slight deficiencies in terms of bias and RMSE compared to the other methods. But these were within an acceptable range, so that no reservations against these algorithms seem indicated. As regards run-time, CS even proved superior by not requiring more time for more trials (as did all other routines), because although the bin frequencies increase, the number of bins does not.

While most of the parameters showed sufficient recovery, we found increased variability for the estimates of ν across all methods, especially when only few trials were available (cf. Figure 8, lower row). This finding is important for applications, when the drift is a focus of the analysis: If one uses ν as an indicator of an individual's information processing speed of perception (in the sense of Voss et al., 2004), a sufficient number of trials should be provided for, otherwise conclusions could be imprecise. From the present results we conclude that at least 100 trials might be a good choice, if justifiable.

Generally, the four ML estimators (two Nelder-Mead implementations, the BFGS, and the nlm() routine) perform altogether well and achieve fairly similar results with comparable performance. Hence, the argument of Lerche et al. (2016)—only methods of one program should be compared—seems to apply only to a certain extent. Rather, we found differences between algorithms to be more pronounced than between implementations. This is good news for practitioners, as they need not bother with which software to use, but only have to select the estimation method, which is most appropriate for their problem. Taking further into account that the algorithms did not differ fundamentally either, the selection can be guided by individual preferences (e.g., whether the whole project is evaluated with R, or not).

The slightly increased bias and RMSE of *a* when estimated with the CS method is likely due to the fact that this method uses binning, thus causing a loss of information. Especially for experiments with fewer trials (50) this effect is visible. Therefore, it seems advisable to use a sufficient number of trials when intending to use the CS method for parameter estimation.

As explained in section 2.2.1, we had to limit ν to the interval ±1 for technical reasons. This could be regarded a limitation of our study, as larger values are observed in real-data applications. However, important tendencies were found: Figure 8 (upper row) reveals that the bias of ν shows a distinct pattern across the eight estimation methods (especially, if the number of trials is low)—it seems likely that this pattern becomes even more pronounced for larger (absolute) values of ν.

### 4.2. Parameter Correlations and Interactions

The analysis of the correlations of the parameter estimates with the true values and among each other yielded mainly results as expected: Correlations with the true values were predominantly close to 1, except for the CS and, in one case, for the K-S method. However, these two did not fall below 0.6. Estimates of parameters of the same kind were also high, again indicating the good recovery properties of the estimators used in this study. But, over and above that, correlations of different parameter estimates were also observed. These were *T*ER of EZ with all *a* estimates and the ν of EZ with the *z* estimates of the other routines. In contrast, the ν estimates of EZ correlated only moderately with the other estimates of ν. Again, we attribute this observation to the bias of EZ invoked by fixing *z* at 0.5 although response bias (i.e., *z* ≠ 0.5) is present in the data.

Using ANOVA for analysing the RMSE, we found some interesting interaction effects: The RMSE of the boundary separation *a* increases considerably the larger *a* and the fewer trials are available. Moreover, the CS method showed the largest RMSE for large *a*. Hence, experiments aiming at large values of *a*, e.g., by involving a speed-accuracy trade-off, should provide for a sufficient number of trials, especially in the accuracy condition. Recently, Stafford et al. (2020) presented a detailed power simulation – using the EZ routine – for detecting true effects of the drift-parameter in the context of a speed-accuracy trade-off design.

Furthermore, we found an interesting interaction of the boundary separation *a* and the drift parameter ν: The smaller *a* the more variability was observed for the estimates of ν. From this finding we have to conclude that focussing on ν (e.g., by using it for assessing an individual's information processing speed) is difficult to combine with a speed condition (thus lacking accuracy), for estimates may be imprecise.

### 4.3. Run-Time

The fast-dm implementation of the various algorithms clearly outperforms the R routines in terms of run-time. This is likely due to two factors: *(i)* the program is written in the programming language C, which delivers an optimized binary (the exe-file, in contrast to the interpreter principle of R and the computationally demanding resampling of the MCMC used in the Bayesian context), and *(ii)* it employs the PDE method, which allows for efficient calculation of the expected response time distributions (which is required at each iteration loop). Thus, if a researcher analyses a large data set, possibly involving many experimental conditions, fast-dm may prove advantageous. Moreover, one can easily switch between the K-S, the CS, and a maximum likelihood method, thus comparing the resulting estimates with respect to the respective (dis-)advantages of either algorithm (e.g., the sensitivity of the ML method for response time outliers).

### 4.4. Conclusion and Outlook

Summarizing, we may state that regarding parameter recovery, all methods performed comparably well except for the EZ method, which showed some deficiencies. It may be applied to generate starting values for an iterative method, but care has to be taken if used for proper parameter estimation. The present study has focussed exclusively on the recovery of the four main parameters *a*, *z*, *T*ER, and ν, following a recommendation of Lerche and Voss (2016).

The following steps would be interesting to take next: (a) considering the variability parameters as well, (b) response time outliers and contaminants, (c) statistical properties of the estimators, or (d) accuracy of the standard errors. Moreover, an important branch of research would be the sensitivity of the methods considered here to misspecified models.

A distinguishing feature of the present study compared to similar ones is the restriction to open source software, thus fostering reproducible research (Fomel and Claerbout, 2009; The Yale Law School Round Table on Data and Core Sharing, 2010).

## Data Availability Statement

The datasets generated for this study are available on request to the corresponding author.

## Author Contributions

RWA developed the concept, performed the simulations, and prepared the manuscript. BG developed the concept and prepared the manuscript. All authors contributed to the article and approved the submitted version.

## Conflict of Interest

The authors declare that the research was conducted in the absence of any commercial or financial relationships that could be construed as a potential conflict of interest.
