# Supplementary material for: Comparing Eight Parameter Estimation Methods for the Ratcliff Diffusion Model Using Free Software
Source: Front Psychol. 2020 Sep 29;11:484737. doi: 10.3389/fpsyg.2020.484737 (PMC7553076; doi:10.3389/fpsyg.2020.484737)
Supplement: Supplementary file 1 [file Data_Sheet_1.pdf]

Online supplement to:

Comparing Eight Parameter Estimation Methods for the Ratcliff Diffusion Model Using Free Software

Rainer W. Alexandrowicz & Bartosz Gula,
Universität Klagenfurt, Austria

List of Tables

1 Descriptive statistics of responses and response times. Notes: Design: Unique parameter combination; a: true boundary separation; z: true starting point; T_{ER}: true encoding and response time; v: true drift parameter; p_{up}: proportion upper boundary crossings; p_{low}: proportion lower boundary crossings; $\bar{x}_{\mathrm{tot}}$: average response time; $\hat{\sigma}_{\mathrm{tot}}$: average standard deviation of response times; $\bar{x}_{\mathrm{up}}$: average response time of upper boundary crossings; $\hat{\sigma}_{\mathrm{up}}$: average standard deviation of upper boundary crossings; $\bar{x}_{\mathrm{low}}$: average response time of lower boundary crossings; $\hat{\sigma}_{\mathrm{low}}$: average standard deviation of lower boundary crossings; . . . 3

2 True parameters and descriptive statistics of data sets failing to deliver a result with the Bayesian estimation method. Notes: a = true boundary separation; z = true starting point; T_{ER} = true encoding and reaction time; ν = true drift parameter; n = number of trials; p_{up} = proportion of upper boundary crossings; p_{low} = proportion of lower boundary crossings; $\bar{x}_{tot}$ = grand mean of response times; $\bar{x}_{up}$ = mean of upper boundary response times; $\bar{x}_{low}$ = mean of lower boundary response times; $\hat{\sigma}^2_{tot}$ = variance of response times; $\hat{\sigma}^2_{up}$ = variance of upper boundary crossing response times; $\hat{\sigma}^2_{low}$ = variance of lower boundary crossing response times; . . . 5

3a Correlation coefficients of descriptive statistics of the sample with the true parameters and the parameter estimates (entire sample). Notes: prop_up = proportion of upper boundary hits; mean_rt = mean response time (all trials); mean_up = mean response time (upper boundary crossings); mean_lo = mean response time (lower boundary crossings); mean_rt = mean response time (all trials); mean_up = mean response time (upper boundary crossings); mean_lo = mean response time (lower boundary crossings); . . . 6

3b Correlation coefficients of descriptive statistics of the sample with the true parameters and the parameter estimates (entire sample). For details see caption of Table 3a. . . . 7

3c Correlation coefficients of descriptive statistics of the sample with the true parameters and the parameter estimates (entire sample). For details see caption of Table 3a. . . . 8

3d Correlation coefficients of descriptive statistics of the sample with the true parameters and the parameter estimates (entire sample). For details see caption of Table 3a. . . . 9

4 Correlation coefficients of true and estimated parameters by number of trials. Notes: Par. = parameter; tot = total sample, 50/100/400 = number of trials; (*) No correlation can be obtained for the z parameter with EZ . . . 15

5a Correlation coefficients of estimated parameters across programs: The Boundary Separation a. . . 16

5b Correlation coefficients of estimated parameters across programs: The Starting Point / Bias z. Note: Asterisks (*) indicate cases, in which no correlation can be obtained because the EZ method sets z to a constant value of 0.5. . . . 17

5c Correlation coefficients of estimated parameters across programs: The encoding and reaction time T_{ER}. . . . 18

5d Correlation coefficients of estimated parameters across programs: The drift parameter ν. . . . 19

6a Correlation coefficients of estimated parameters across programs: The Boundary Separation a. . . 20

6b Correlation coefficients of estimated parameters across programs: The Starting Point / Bias z . . 21

6c Correlation coefficients of estimated parameters across programs: The encoding and reaction time T_{ER}. . . . 22

6d Correlation coefficients of estimated parameters across programs: The drift parameter ν. . . . 23

7a Correlation coefficients of estimated parameters across programs: The Boundary Separation a. . 24
7b Correlation coefficients of estimated parameters across programs: The Starting Point / Bias z . . 25
7c Correlation coefficients of estimated parameters across programs: The encoding and reaction time T_{ER}. 26
7d Correlation coefficients of estimated parameters across programs: The drift parameter ν. 27
8a Correlation coefficients of estimated parameters across programs: The Boundary Separation a. . 28
8b Correlation coefficients of estimated parameters across programs: The Starting Point / Bias z . . 29
8c Correlation coefficients of estimated parameters across programs: The encoding and reaction time T_{ER}. 30
8d Correlation coefficients of estimated parameters across programs: The drift parameter ν. 31
9 Descriptive statistics and parameter recovery performance measures for the boundary separation parameter a. Notes: method = estimation method; trials = number of trials; n = effective number of available values (only here); mean = mean of parameter estimates; sd = standard deviation of parameter estimates; bias = mean parameter minus true parameter; relbias = bias divided by true parameter; mse = mean squared error; rmse = root mean squared error. 36
10 Descriptive statistics and parameter recovery performance measures for the starting point z. Notes as in Table 9. 38
11 Descriptive statistics and parameter recovery performance measures for the encoding and reaction time T_{ER}. Notes as in Table 9. 40
12 Descriptive statistics and parameter recovery performance measures for the drift parameter ν. Notes as in Table 9 and: the relative bias cannot be computed for $\nu = 0$, the respective entries are indicated by (+/−) Inf (for infinity would result after division by zero). 42

List of Figures

1 Histograms of the Potential Scale Reduction Factors for the boundary separation a, the starting point z, the encoding and reaction time T_{ER}, and the drift parameter ν. 5
2a Box plots of the estimates of boundary separation a. 11
2b Box plots of the estimates of the bias parameter z. Note: EZ cannot estimate z, but rather sets the value to 0.5. 12
2c Box plots of the estimates of the encoding and response time parameter T_{ER}. Note: The bold (red) line in the upper left diagram indicates the zero-line. Estimates below this line represent model violations. 13
2d Box plots of the estimates of the drift parameter ν. 14
3a Scatter plots of the estimates of boundary separation a. 32
3b Scatter plots of the estimates of the bias parameter z. 33
3c Scatter plots of the estimates of the encoding and response time parameter T_{ER}. Note: The degenerate structure in the first row and the first column is a result of the EZ method not estimating z, but rather setting it to 0.5. 34
3d Scatter plots of the estimates of the drift parameter ν. 35

1 Descriptive Statistics of Responses and Response Times

Table 1: Descriptive statistics of responses and response times. Notes: Design: Unique parameter combination; a: true boundary separation; z: true starting point; T_{ER}: true encoding and response time; v: true drift parameter; p_{up}: proportion upper boundary crossings; p_{low}: proportion lower boundary crossings; $\bar{x}_{\mathrm{tot}}$: average response time; $\hat{\sigma}_{\mathrm{tot}}$: average standard deviation of response times; $\bar{x}_{\mathrm{up}}$: average response time of upper boundary crossings; $\hat{\sigma}_{\mathrm{up}}$: average standard deviation of upper boundary crossings; $\bar{x}_{\mathrm{low}}$: average response time of lower boundary crossings; $\hat{\sigma}_{\mathrm{low}}$: average standard deviation of lower boundary crossings;

Design	a	z	T_{ER}	v	p_{up}	p_{low}	$\bar{x}_{\mathrm{tot}}$	$\hat{\sigma}_{\mathrm{tot}}$	$\bar{x}_{\mathrm{up}}$	$\hat{\sigma}_{\mathrm{up}}$	$\bar{x}_{\mathrm{low}}$	$\hat{\sigma}_{\mathrm{low}}$
1	0.50	0.20	0.10	-1.00	0.13	0.87	0.14	0.04	0.18	0.05	0.13	0.04
2	1.00	0.20	0.10	-1.00	0.08	0.92	0.23	0.16	0.37	0.15	0.21	0.15
3	1.50	0.20	0.10	-1.00	0.04	0.96	0.32	0.27	0.70	0.33	0.31	0.26
4	2.00	0.20	0.10	-1.00	0.02	0.98	0.44	0.43	1.15	0.49	0.43	0.41
5	0.50	0.50	0.10	-1.00	0.39	0.61	0.16	0.05	0.16	0.05	0.16	0.05
6	1.00	0.50	0.10	-1.00	0.26	0.74	0.34	0.19	0.33	0.17	0.34	0.19
7	1.50	0.50	0.10	-1.00	0.16	0.84	0.58	0.37	0.58	0.35	0.58	0.37
8	2.00	0.50	0.10	-1.00	0.12	0.88	0.84	0.58	0.80	0.49	0.84	0.58
9	0.50	0.80	0.10	-1.00	0.71	0.29	0.14	0.05	0.13	0.04	0.18	0.05
10	1.00	0.80	0.10	-1.00	0.61	0.39	0.28	0.19	0.21	0.14	0.39	0.19
11	1.50	0.80	0.10	-1.00	0.54	0.46	0.51	0.40	0.32	0.27	0.74	0.41
12	2.00	0.80	0.10	-1.00	0.42	0.58	0.84	0.63	0.46	0.42	1.13	0.61
13	0.50	0.20	0.30	-1.00	0.13	0.87	0.33	0.04	0.38	0.05	0.33	0.04
14	1.00	0.20	0.30	-1.00	0.07	0.93	0.42	0.15	0.63	0.18	0.41	0.14
15	1.50	0.20	0.30	-1.00	0.04	0.96	0.54	0.31	0.94	0.32	0.52	0.29
16	2.00	0.20	0.30	-1.00	0.02	0.98	0.66	0.45	1.25	0.48	0.65	0.44
17	0.50	0.50	0.30	-1.00	0.38	0.62	0.36	0.05	0.36	0.04	0.36	0.05
18	1.00	0.50	0.30	-1.00	0.28	0.72	0.53	0.19	0.53	0.17	0.54	0.19
19	1.50	0.50	0.30	-1.00	0.17	0.83	0.76	0.38	0.77	0.39	0.76	0.37
20	2.00	0.50	0.30	-1.00	0.12	0.88	1.07	0.59	1.03	0.58	1.08	0.58
21	0.50	0.80	0.30	-1.00	0.72	0.28	0.34	0.05	0.33	0.04	0.38	0.05
22	1.00	0.80	0.30	-1.00	0.60	0.40	0.49	0.19	0.41	0.15	0.61	0.19
23	1.50	0.80	0.30	-1.00	0.52	0.48	0.71	0.38	0.51	0.27	0.93	0.36
24	2.00	0.80	0.30	-1.00	0.43	0.57	1.02	0.62	0.64	0.41	1.30	0.58
25	0.50	0.20	0.50	-1.00	0.13	0.87	0.54	0.04	0.58	0.05	0.53	0.04
26	1.00	0.20	0.50	-1.00	0.07	0.93	0.62	0.14	0.77	0.15	0.60	0.13
27	1.50	0.20	0.50	-1.00	0.04	0.96	0.73	0.30	1.05	0.29	0.72	0.29
28	2.00	0.20	0.50	-1.00	0.02	0.98	0.85	0.44	1.42	0.55	0.84	0.43
29	0.50	0.50	0.50	-1.00	0.36	0.64	0.56	0.05	0.56	0.05	0.57	0.05
30	1.00	0.50	0.50	-1.00	0.26	0.74	0.73	0.18	0.73	0.18	0.73	0.18
31	1.50	0.50	0.50	-1.00	0.19	0.81	0.98	0.39	1.01	0.39	0.98	0.39
32	2.00	0.50	0.50	-1.00	0.12	0.88	1.27	0.58	1.22	0.52	1.27	0.59
33	0.50	0.80	0.50	-1.00	0.72	0.28	0.54	0.05	0.53	0.04	0.58	0.05
34	1.00	0.80	0.50	-1.00	0.63	0.37	0.68	0.18	0.60	0.13	0.80	0.19
35	1.50	0.80	0.50	-1.00	0.51	0.49	0.93	0.40	0.73	0.28	1.14	0.41
36	2.00	0.80	0.50	-1.00	0.42	0.58	1.25	0.62	0.84	0.37	1.54	0.59
37	0.50	0.20	0.10	-0.50	0.18	0.82	0.14	0.05	0.18	0.05	0.13	0.04
38	1.00	0.20	0.10	-0.50	0.13	0.87	0.25	0.18	0.43	0.18	0.22	0.16
39	1.50	0.20	0.10	-0.50	0.11	0.89	0.41	0.39	0.84	0.47	0.35	0.34
40	2.00	0.20	0.10	-0.50	0.08	0.92	0.57	0.61	1.29	0.69	0.51	0.54
41	0.50	0.50	0.10	-0.50	0.44	0.56	0.16	0.05	0.16	0.05	0.16	0.05
42	1.00	0.50	0.10	-0.50	0.39	0.61	0.34	0.19	0.34	0.18	0.34	0.19
43	1.50	0.50	0.10	-0.50	0.34	0.66	0.64	0.42	0.61	0.38	0.65	0.43
44	2.00	0.50	0.10	-0.50	0.26	0.74	1.03	0.70	1.01	0.68	1.04	0.71
45	0.50	0.80	0.10	-0.50	0.74	0.26	0.14	0.05	0.13	0.04	0.18	0.05
46	1.00	0.80	0.10	-0.50	0.69	0.31	0.27	0.19	0.21	0.15	0.40	0.19
47	1.50	0.80	0.10	-0.50	0.67	0.33	0.50	0.44	0.37	0.34	0.78	0.46
48	2.00	0.80	0.10	-0.50	0.61	0.39	0.84	0.75	0.53	0.55	1.31	0.76
49	0.50	0.20	0.30	-0.50	0.18	0.82	0.34	0.05	0.38	0.05	0.33	0.04
50	1.00	0.20	0.30	-0.50	0.12	0.88	0.44	0.17	0.61	0.18	0.42	0.15
51	1.50	0.20	0.30	-0.50	0.08	0.92	0.59	0.37	1.03	0.46	0.55	0.33
52	2.00	0.20	0.30	-0.50	0.08	0.92	0.82	0.66	1.57	0.77	0.75	0.61
53	0.50	0.50	0.30	-0.50	0.44	0.56	0.36	0.05	0.36	0.05	0.36	0.05
54	1.00	0.50	0.30	-0.50	0.38	0.62	0.54	0.20	0.54	0.20	0.54	0.19
55	1.50	0.50	0.30	-0.50	0.30	0.70	0.83	0.41	0.84	0.39	0.83	0.41
56	2.00	0.50	0.30	-0.50	0.27	0.73	1.23	0.73	1.24	0.75	1.22	0.72
57	0.50	0.80	0.30	-0.50	0.74	0.26	0.34	0.05	0.33	0.04	0.38	0.05
58	1.00	0.80	0.30	-0.50	0.71	0.29	0.48	0.20	0.41	0.14	0.64	0.22
59	1.50	0.80	0.30	-0.50	0.64	0.36	0.72	0.46	0.58	0.40	0.98	0.42
60	2.00	0.80	0.30	-0.50	0.62	0.38	1.02	0.71	0.74	0.56	1.47	0.69
61	0.50	0.20	0.50	-0.50	0.16	0.84	0.54	0.05	0.58	0.05	0.53	0.04
62	1.00	0.20	0.50	-0.50	0.12	0.88	0.64	0.18	0.80	0.17	0.62	0.16
63	1.50	0.20	0.50	-0.50	0.10	0.90	0.79	0.35	1.23	0.38	0.75	0.31
64	2.00	0.20	0.50	-0.50	0.08	0.92	0.99	0.60	1.67	0.68	0.93	0.56
65	0.50	0.50	0.50	-0.50	0.44	0.56	0.56	0.05	0.56	0.05	0.56	0.05
66	1.00	0.50	0.50	-0.50	0.39	0.61	0.74	0.20	0.74	0.19	0.74	0.20
67	1.50	0.50	0.50	-0.50	0.32	0.68	1.03	0.42	1.02	0.41	1.03	0.42
68	2.00	0.50	0.50	-0.50	0.26	0.74	1.43	0.77	1.46	0.76	1.41	0.77
69	0.50	0.80	0.50	-0.50	0.74	0.26	0.54	0.05	0.53	0.04	0.58	0.05
70	1.00	0.80	0.50	-0.50	0.71	0.29	0.67	0.20	0.61	0.16	0.80	0.20
71	1.50	0.80	0.50	-0.50	0.65	0.35	0.91	0.45	0.76	0.35	1.19	0.47
72	2.00	0.80	0.50	-0.50	0.61	0.39	1.20	0.71	0.92	0.55	1.64	0.69
73	0.50	0.20	0.10	0.00	0.21	0.79	0.14	0.05	0.18	0.05	0.13	0.04
74	1.00	0.20	0.10	0.00	0.21	0.79	0.26	0.18	0.42	0.20	0.22	0.14
75	1.50	0.20	0.10	0.00	0.21	0.79	0.48	0.43	0.82	0.42	0.39	0.38
76	2.00	0.20	0.10	0.00	0.20	0.80	0.74	0.78	1.33	0.84	0.59	0.66
77	0.50	0.50	0.10	0.00	0.51	0.49	0.16	0.05	0.16	0.05	0.16	0.05
78	1.00	0.50	0.10	0.00	0.50	0.50	0.35	0.20	0.36	0.21	0.34	0.18
79	1.50	0.50	0.10	0.00	0.49	0.51	0.66	0.46	0.68	0.47	0.65	0.44
80	2.00	0.50	0.10	0.00	0.50	0.50	1.08	0.79	1.12	0.84	1.05	0.73
81	0.50	0.80	0.10	0.00	0.81	0.19	0.14	0.05	0.13	0.04	0.18	0.05
82	1.00	0.80	0.10	0.00	0.78	0.22	0.26	0.18	0.21	0.15	0.42	0.19
83	1.50	0.80	0.10	0.00	0.80	0.20	0.45	0.41	0.36	0.33	0.80	0.44
84	2.00	0.80	0.10	0.00	0.81	0.19	0.72	0.71	0.57	0.61	1.34	0.78
85	0.50	0.20	0.30	0.00	0.20	0.80	0.34	0.05	0.38	0.06	0.33	0.04
86	1.00	0.20	0.30	0.00	0.19	0.81	0.46	0.18	0.61	0.18	0.42	0.15
87	1.50	0.20	0.30	0.00	0.20	0.80	0.66	0.43	1.06	0.47	0.56	0.34

88	2.00	0.20	0.30	0.00	0.18	0.82	0.95	0.78	1.64	0.91	0.79	0.64
89	0.50	0.50	0.30	0.00	0.51	0.49	0.36	0.05	0.36	0.05	0.36	0.05
90	1.00	0.50	0.30	0.00	0.52	0.48	0.54	0.19	0.54	0.19	0.55	0.20
91	1.50	0.50	0.30	0.00	0.51	0.49	0.89	0.47	0.90	0.45	0.89	0.49
92	2.00	0.50	0.30	0.00	0.49	0.51	1.37	0.87	1.37	0.84	1.38	0.89
93	0.50	0.80	0.30	0.00	0.80	0.20	0.34	0.05	0.33	0.04	0.38	0.05
94	1.00	0.80	0.30	0.00	0.79	0.21	0.45	0.18	0.42	0.15	0.58	0.18
95	1.50	0.80	0.30	0.00	0.78	0.22	0.68	0.46	0.58	0.39	1.03	0.47
96	2.00	0.80	0.30	0.00	0.81	0.19	0.92	0.73	0.77	0.60	1.58	0.82
97	0.50	0.20	0.50	0.00	0.20	0.80	0.54	0.04	0.58	0.05	0.53	0.04
98	1.00	0.20	0.50	0.00	0.19	0.81	0.66	0.18	0.81	0.18	0.63	0.16
99	1.50	0.20	0.50	0.00	0.19	0.81	0.86	0.42	1.18	0.44	0.78	0.37
100	2.00	0.20	0.50	0.00	0.21	0.79	1.17	0.79	1.70	0.75	1.03	0.74
101	0.50	0.50	0.50	0.00	0.51	0.49	0.56	0.05	0.56	0.05	0.56	0.05
102	1.00	0.50	0.50	0.00	0.48	0.52	0.75	0.21	0.74	0.20	0.75	0.21
103	1.50	0.50	0.50	0.00	0.50	0.50	1.08	0.47	1.10	0.49	1.06	0.44
104	2.00	0.50	0.50	0.00	0.53	0.47	1.46	0.80	1.47	0.79	1.45	0.79
105	0.50	0.80	0.50	0.00	0.81	0.19	0.54	0.04	0.53	0.04	0.58	0.05
106	1.00	0.80	0.50	0.00	0.80	0.20	0.65	0.18	0.61	0.15	0.81	0.21
107	1.50	0.80	0.50	0.00	0.77	0.23	0.87	0.44	0.76	0.36	1.24	0.49
108	2.00	0.80	0.50	0.00	0.81	0.19	1.12	0.75	0.97	0.63	1.72	0.84
109	0.50	0.20	0.10	0.50	0.25	0.75	0.14	0.05	0.18	0.05	0.13	0.04
110	1.00	0.20	0.10	0.50	0.27	0.73	0.27	0.19	0.43	0.20	0.21	0.15
111	1.50	0.20	0.10	0.50	0.33	0.67	0.50	0.44	0.81	0.45	0.35	0.34
112	2.00	0.20	0.10	0.50	0.40	0.60	0.84	0.73	1.35	0.73	0.50	0.48
113	0.50	0.50	0.10	0.50	0.57	0.43	0.16	0.05	0.17	0.05	0.16	0.04
114	1.00	0.50	0.10	0.50	0.60	0.40	0.35	0.19	0.35	0.20	0.35	0.18
115	1.50	0.50	0.10	0.50	0.69	0.31	0.66	0.46	0.67	0.46	0.66	0.42
116	2.00	0.50	0.10	0.50	0.72	0.28	1.02	0.72	1.02	0.71	1.02	0.72
117	0.50	0.80	0.10	0.50	0.85	0.15	0.13	0.04	0.13	0.03	0.18	0.05
118	1.00	0.80	0.10	0.50	0.87	0.13	0.24	0.17	0.22	0.16	0.42	0.17
119	1.50	0.80	0.10	0.50	0.89	0.11	0.41	0.39	0.36	0.35	0.79	0.44
120	2.00	0.80	0.10	0.50	0.93	0.07	0.57	0.59	0.51	0.55	1.27	0.70
121	0.50	0.20	0.30	0.50	0.24	0.76	0.34	0.05	0.38	0.04	0.33	0.04
122	1.00	0.20	0.30	0.50	0.30	0.70	0.47	0.19	0.62	0.20	0.41	0.14
123	1.50	0.20	0.30	0.50	0.33	0.67	0.69	0.42	0.98	0.41	0.56	0.34
124	2.00	0.20	0.30	0.50	0.39	0.61	1.06	0.82	1.53	0.84	0.75	0.58
125	0.50	0.50	0.30	0.50	0.57	0.43	0.36	0.05	0.36	0.05	0.36	0.05
126	1.00	0.50	0.30	0.50	0.65	0.35	0.54	0.19	0.54	0.19	0.54	0.19
127	1.50	0.50	0.30	0.50	0.66	0.34	0.85	0.45	0.87	0.45	0.82	0.43
128	2.00	0.50	0.30	0.50	0.76	0.24	1.21	0.71	1.23	0.73	1.17	0.63
129	0.50	0.80	0.30	0.50	0.85	0.15	0.34	0.05	0.33	0.04	0.38	0.05
130	1.00	0.80	0.30	0.50	0.87	0.13	0.43	0.17	0.41	0.14	0.61	0.20
131	1.50	0.80	0.30	0.50	0.90	0.10	0.59	0.34	0.56	0.32	0.89	0.34
132	2.00	0.80	0.30	0.50	0.93	0.07	0.81	0.63	0.74	0.57	1.58	0.72
133	0.50	0.20	0.50	0.50	0.25	0.75	0.54	0.05	0.58	0.05	0.53	0.04
134	1.00	0.20	0.50	0.50	0.28	0.72	0.67	0.18	0.80	0.20	0.61	0.14
135	1.50	0.20	0.50	0.50	0.37	0.63	0.91	0.44	1.18	0.44	0.75	0.34
136	2.00	0.20	0.50	0.50	0.39	0.61	1.23	0.72	1.68	0.73	0.94	0.54
137	0.50	0.50	0.50	0.50	0.56	0.44	0.56	0.05	0.56	0.05	0.56	0.05
138	1.00	0.50	0.50	0.50	0.62	0.38	0.74	0.20	0.75	0.20	0.74	0.20
139	1.50	0.50	0.50	0.50	0.68	0.32	1.04	0.44	1.06	0.44	1.02	0.42
140	2.00	0.50	0.50	0.50	0.75	0.25	1.41	0.73	1.40	0.74	1.43	0.69
141	0.50	0.80	0.50	0.50	0.85	0.15	0.54	0.05	0.53	0.04	0.58	0.05
142	1.00	0.80	0.50	0.50	0.86	0.14	0.65	0.18	0.63	0.17	0.83	0.18
143	1.50	0.80	0.50	0.50	0.90	0.10	0.80	0.37	0.76	0.35	1.16	0.38
144	2.00	0.80	0.50	0.50	0.93	0.07	1.00	0.64	0.94	0.57	1.78	0.75
145	0.50	0.20	0.10	1.00	0.30	0.70	0.14	0.05	0.18	0.05	0.13	0.04
146	1.00	0.20	0.10	1.00	0.40	0.60	0.29	0.20	0.40	0.19	0.22	0.16
147	1.50	0.20	0.10	1.00	0.47	0.53	0.50	0.39	0.72	0.38	0.30	0.27
148	2.00	0.20	0.10	1.00	0.54	0.46	0.83	0.64	1.14	0.61	0.47	0.45
149	0.50	0.50	0.10	1.00	0.64	0.36	0.16	0.05	0.16	0.05	0.16	0.05
150	1.00	0.50	0.10	1.00	0.72	0.28	0.33	0.18	0.33	0.18	0.34	0.19
151	1.50	0.50	0.10	1.00	0.81	0.19	0.57	0.38	0.58	0.39	0.55	0.35
152	2.00	0.50	0.10	1.00	0.88	0.12	0.86	0.58	0.86	0.58	0.87	0.58
153	0.50	0.80	0.10	1.00	0.88	0.12	0.13	0.04	0.13	0.03	0.18	0.05
154	1.00	0.80	0.10	1.00	0.92	0.08	0.23	0.17	0.21	0.16	0.39	0.18
155	1.50	0.80	0.10	1.00	0.96	0.04	0.32	0.27	0.30	0.25	0.66	0.29
156	2.00	0.80	0.10	1.00	0.97	0.03	0.45	0.42	0.43	0.40	1.15	0.51
157	0.50	0.20	0.30	1.00	0.28	0.72	0.34	0.05	0.38	0.05	0.33	0.04
158	1.00	0.20	0.30	1.00	0.37	0.63	0.47	0.17	0.58	0.17	0.41	0.14
159	1.50	0.20	0.30	1.00	0.48	0.52	0.71	0.40	0.92	0.38	0.52	0.29
160	2.00	0.20	0.30	1.00	0.53	0.47	1.02	0.64	1.34	0.60	0.65	0.44
161	0.50	0.50	0.30	1.00	0.63	0.37	0.36	0.05	0.36	0.05	0.36	0.05
162	1.00	0.50	0.30	1.00	0.74	0.26	0.54	0.19	0.53	0.19	0.54	0.19
163	1.50	0.50	0.30	1.00	0.81	0.19	0.76	0.35	0.76	0.35	0.80	0.35
164	2.00	0.50	0.30	1.00	0.88	0.12	1.09	0.63	1.08	0.60	1.21	0.72
165	0.50	0.80	0.30	1.00	0.87	0.13	0.34	0.04	0.33	0.04	0.38	0.05
166	1.00	0.80	0.30	1.00	0.91	0.09	0.42	0.15	0.40	0.13	0.59	0.18
167	1.50	0.80	0.30	1.00	0.96	0.04	0.53	0.29	0.51	0.27	0.90	0.39
168	2.00	0.80	0.30	1.00	0.98	0.02	0.67	0.47	0.66	0.46	1.19	0.43
169	0.50	0.20	0.50	1.00	0.27	0.73	0.54	0.05	0.58	0.05	0.53	0.04
170	1.00	0.20	0.50	1.00	0.40	0.60	0.68	0.19	0.80	0.19	0.61	0.14
171	1.50	0.20	0.50	1.00	0.47	0.53	0.90	0.38	1.13	0.37	0.70	0.26
172	2.00	0.20	0.50	1.00	0.55	0.45	1.20	0.61	1.51	0.58	0.83	0.38
173	0.50	0.50	0.50	1.00	0.61	0.39	0.56	0.05	0.56	0.05	0.56	0.05
174	1.00	0.50	0.50	1.00	0.73	0.27	0.73	0.18	0.73	0.17	0.72	0.18
175	1.50	0.50	0.50	1.00	0.81	0.19	1.00	0.40	0.99	0.39	1.01	0.39
176	2.00	0.50	0.50	1.00	0.89	0.11	1.25	0.58	1.25	0.59	1.20	0.46
177	0.50	0.80	0.50	1.00	0.87	0.13	0.53	0.04	0.53	0.04	0.58	0.04
178	1.00	0.80	0.50	1.00	0.91	0.09	0.62	0.15	0.60	0.13	0.81	0.18
179	1.50	0.80	0.50	1.00	0.96	0.04	0.74	0.31	0.72	0.29	1.14	0.31
180	2.00	0.80	0.50	1.00	0.98	0.03	0.86	0.45	0.85	0.44	1.51	0.51

2 Bayesian Failures

Table 2: True parameters and descriptive statistics of data sets failing to deliver a result with the Bayesian estimation method. Notes: a = true boundary separation; z = true starting point; T_{ER} = true encoding and reaction time; ν = true drift parameter; n = number of trials; p_{up} = proportion of upper boundary crossings; p_{low} = proportion of lower boundary crossings; $\bar{x}_{tot}$ = grand mean of response times; $\bar{x}_{up}$ = mean of upper boundary response times; $\bar{x}_{low}$ = mean of lower boundary response times; $\hat{\sigma}^2_{tot}$ = variance of response times; $\hat{\sigma}^2_{up}$ = variance of upper boundary crossing response times; $\hat{\sigma}^2_{low}$ = variance of lower boundary crossing response times;

a	z	T_{ER}	ν	n	p_{up}	p_{low}	$\bar{x}_{tot}$	$\bar{x}_{up}$	$\bar{x}_{low}$	$\hat{\sigma}^2_{tot}$	$\hat{\sigma}^2_{up}$	$\hat{\sigma}^2_{low}$
2	0.2	0.1	0	400	0.23	0.77	0.74	1.33	0.56	0.58	1	0,33
2	0.2	0.1	0.5	400	0.39	0.61	0.85	1.39	0.51	0.65	0.83	0,24
2	0.2	0.3	-0.5	400	0.08	0.92	0.8	1.47	0.75	0.51	0.58	0,47
2	0.2	0.3	-0.5	400	0.09	0.91	0.82	1.46	0.75	0.46	0.62	0,4
2	0.2	0.3	0	100	0.23	0.77	1.04	1.79	0.81	0.84	1.57	0,42
2	0.2	0.3	0	400	0.2	0.8	0.95	1.57	0.79	0.63	1.07	0,4
2	0.2	0.3	0.5	100	0.37	0.63	1	1.58	0.66	0.7	1.11	0,15
2	0.2	0.3	0.5	400	0.38	0.62	1.02	1.54	0.71	0.61	0.75	0,27
2	0.2	0.3	0.5	50	0.4	0.6	1.08	1.17	1.02	1.42	0.22	2,25
2	0.2	0.3	1	400	0.55	0.45	1.04	1.34	0.68	0.46	0.4	0,29
2	0.2	0.5	-0.5	400	0.08	0.92	1.03	1.82	0.97	0.51	0.74	0,44
2	0.2	0.5	0	100	0.2	0.8	1.22	1.65	1.12	0.82	0.38	0,88
2	0.2	0.5	0	400	0.19	0.81	1.16	1.77	1.02	0.65	0.83	0,5
2	0.2	0.5	0	400	0.19	0.81	1.16	1.81	1.01	0.68	0.8	0,54
2	0.2	0.5	0.5	400	0.4	0.6	1.24	1.75	0.9	0.69	0.89	0,26
2	0.2	0.5	0.5	400	0.42	0.58	1.27	1.77	0.91	0.61	0.55	0,35
2	0.5	0.1	-0.5	400	0.26	0.74	1.09	1.04	1.1	0.6	0.52	0,63
2	0.5	0.1	0	400	0.5	0.5	1.13	1.09	1.16	0.69	0.62	0,75
2	0.5	0.1	0	50	0.4	0.6	1.1	1.31	0.96	0.93	1.8	0,34
2	0.5	0.1	0.5	400	0.68	0.32	1.02	1.05	0.98	0.58	0.64	0,45
2	0.5	0.3	-0.5	400	0.24	0.76	1.2	1.19	1.2	0.59	0.8	0,53
2	0.5	0.3	-0.5	400	0.26	0.74	1.25	1.2	1.27	0.66	0.56	0,69
2	0.5	0.3	-0.5	400	0.28	0.72	1.26	1.23	1.27	0.64	0.67	0,63
2	0.5	0.3	0	100	0.58	0.42	1.55	1.49	1.64	1.28	0.72	2,07
2	0.5	0.3	0	400	0.49	0.51	1.3	1.35	1.25	0.69	0.71	0,67
2	0.5	0.3	0.5	400	0.74	0.26	1.22	1.22	1.21	0.59	0.59	0,59
2	0.5	0.5	-0.5	100	0.22	0.78	1.6	1.63	1.59	1	0.68	1,11
2	0.5	0.5	-0.5	400	0.28	0.72	1.43	1.39	1.45	0.61	0.82	0,53
2	0.5	0.5	-0.5	50	0.3	0.7	1.47	1.53	1.44	1.33	0.59	1,67
2	0.5	0.5	0	100	0.47	0.53	1.38	1.4	1.37	0.75	0.61	0,89
2	0.5	0.5	0	100	0.52	0.48	1.48	1.54	1.41	0.88	0.72	1,08
2	0.5	0.5	0	400	0.49	0.51	1.51	1.52	1.51	0.76	0.84	0,7
2	0.5	0.5	0	400	0.55	0.46	1.5	1.56	1.44	0.79	0.98	0,56
2	0.5	0.5	0.5	100	0.76	0.24	1.46	1.47	1.42	0.75	0.86	0,43
2	0.5	0.5	0.5	400	0.72	0.28	1.48	1.43	1.61	0.6	0.55	0,71
2	0.5	0.5	0.5	400	0.75	0.25	1.45	1.45	1.46	0.58	0.56	0,63
2	0.5	0.5	1	400	0.88	0.12	1.3	1.29	1.38	0.46	0.49	0,29
2	0.8	0.1	-0.5	400	0.58	0.42	0.84	0.53	1.27	0.59	0.42	0,52
2	0.8	0.1	0	400	0.8	0.2	0.74	0.58	1.4	0.64	0.47	0,77
2	0.8	0.1	0.5	400	0.91	0.09	0.6	0.53	1.28	0.44	0.34	0,97
2	0.8	0.1	0.5	50	0.98	0.02	0.71	0.68	2.11	0.96	0.94	NA
2	0.8	0.3	-0.5	400	0.64	0.36	1.08	0.78	1.62	0.7	0.33	0,91
2	0.8	0.3	-0.5	400	0.67	0.33	0.96	0.68	1.52	0.49	0.2	0,6
2	0.8	0.3	0	400	0.8	0.2	1	0.83	1.68	0.7	0.5	0,98
2	0.8	0.3	0.5	100	0.96	0.04	0.82	0.8	1.33	0.48	0.48	0,2
2	0.8	0.3	0.5	400	0.92	0.08	0.81	0.75	1.46	0.4	0.32	0,94
2	0.8	0.3	0.5	400	0.93	0.07	0.82	0.75	1.81	0.6	0.5	0,93
2	0.8	0.5	-0.5	400	0.61	0.39	1.22	0.95	1.63	0.65	0.52	0,57
2	0.8	0.5	-0.5	400	0.69	0.31	1.22	0.95	1.83	0.68	0.39	0,8
2	0.8	0.5	0	100	0.8	0.2	1.27	1.11	1.91	1.04	0.99	0,79
2	0.8	0.5	0	100	0.87	0.13	1.04	0.96	1.59	0.64	0.63	0,4
2	0.8	0.5	0	400	0.8	0.2	1.08	0.94	1.64	0.53	0.44	0,53
2	0.8	0.5	0.5	100	0.93	0.07	0.98	0.93	1.62	0.6	0.6	0,14
2	0.8	0.5	0.5	50	0.9	0.1	1.2	0.99	3.08	0.99	0.35	3,36

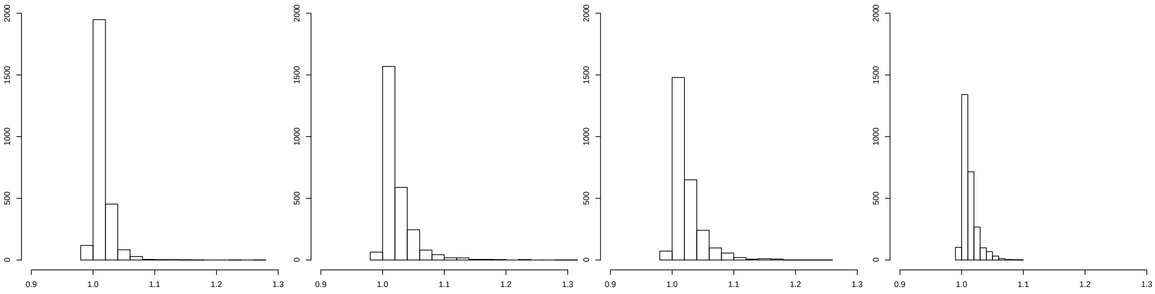

Figure 1: Histograms of the Potential Scale Reduction Factors for the boundary separation a, the starting point z, the encoding and reaction time T_{ER}, and the drift parameter ν.

3 Descriptive Statistics of the Samples and Parameter Estimates

Table 3a: Correlation coefficients of descriptive statistics of the sample with the true parameters and the parameter estimates (entire sample). Notes: prop_up = proportion of upper boundary hits; mean_rt = mean response time (all trials); mean_up = mean response time (upper boundary crossings); mean_lo = mean response time (lower boundary crossings); mean_rt = mean response time (all trials); mean_up = mean response time (upper boundary crossings); mean_lo = mean response time (lower boundary crossings);

all	prop_up	mean_rt	mean_up	mean_lo	var_rt	var_up	var_lo
a_true	−0.005	0.758	0.722	0.717	0.803	0.677	0.620
a_R_EZ	−0.010	0.710	0.715	0.712	0.808	0.702	0.656
a_R_BY	−0.014	0.750	0.726	0.721	0.825	0.684	0.645
a_R_BF	−0.013	0.731	0.717	0.710	0.791	0.678	0.622
a_R_NL	−0.021	0.648	0.706	0.632	0.704	0.666	0.555
a_R_NM	−0.005	0.698	0.685	0.707	0.755	0.647	0.620
a_F_ML	−0.013	0.698	0.702	0.689	0.756	0.664	0.604
a_F_KS	0.004	0.677	0.668	0.677	0.741	0.638	0.589
a_F_CS	−0.021	0.533	0.554	0.541	0.598	0.531	0.483
z_true	0.793	−0.001	−0.367	0.360	−0.012	−0.145	0.115
z_R_EZ	(*)	(*)	(*)	(*)	(*)	(*)	(*)
z_R_BY	0.795	0.001	−0.372	0.366	−0.012	−0.148	0.121
z_R_BF	0.792	0.000	−0.371	0.366	−0.009	−0.144	0.119
z_R_NL	0.793	−0.001	−0.367	0.362	−0.013	−0.144	0.114
z_R_NM	0.793	0.001	−0.369	0.366	−0.009	−0.144	0.119
z_F_ML	0.792	−0.001	−0.371	0.366	−0.010	−0.145	0.118
z_F_KS	0.779	−0.005	−0.369	0.357	−0.014	−0.145	0.116
z_F_CS	0.779	−0.000	−0.362	0.356	−0.008	−0.138	0.117
t_true	−0.002	0.520	0.414	0.421	0.008	−0.000	0.015
t_R_EZ	0.002	0.330	0.138	0.145	−0.269	−0.255	−0.234
t_R_BY	−0.002	0.518	0.409	0.423	−0.018	−0.019	−0.004
t_R_BF	−0.002	0.530	0.422	0.431	0.015	0.005	0.025
t_R_NL	−0.000	0.525	0.417	0.426	0.010	0.002	0.021
t_R_NM	−0.001	0.530	0.422	0.431	0.016	0.006	0.026
t_F_ML	−0.001	0.532	0.424	0.433	0.019	0.008	0.028
t_F_KS	−0.006	0.522	0.415	0.420	0.013	0.002	0.030
t_F_CS	−0.000	0.501	0.399	0.408	0.008	−0.003	0.025
v_true	0.533	−0.001	0.012	−0.006	0.006	0.015	0.001
v_R_EZ	0.889	−0.009	−0.187	0.170	−0.005	−0.056	0.047
v_R_BY	0.564	−0.013	0.004	−0.024	−0.003	0.010	−0.007
v_R_BF	0.507	−0.016	0.011	−0.035	−0.003	0.012	−0.010
v_R_NL	0.506	−0.013	0.009	−0.029	0.004	0.015	−0.003
v_R_NM	0.480	−0.021	0.009	−0.042	−0.010	0.006	−0.015
v_F_ML	0.505	−0.019	0.007	−0.037	−0.006	0.009	−0.012
v_F_KS	0.524	−0.017	−0.013	−0.013	−0.003	−0.003	−0.001
v_F_CS	0.495	−0.003	−0.000	−0.002	0.008	0.008	0.006

Table 3b: Correlation coefficients of descriptive statistics of the sample with the true parameters and the parameter estimates (entire sample). For details see caption of Table 3a.

$n = 50$	prop_up	mean_rt	mean_up	mean_lo	var_rt	var_up	var_lo
a_true	−0.001	0.751	0.714	0.696	0.766	0.644	0.512
a_R_EZ	−0.003	0.713	0.702	0.702	0.806	0.682	0.575
a_R_BY	−0.008	0.754	0.724	0.717	0.802	0.661	0.545
a_R_BF	−0.008	0.691	0.690	0.677	0.727	0.631	0.501
a_R_NL	−0.025	0.536	0.663	0.523	0.573	0.608	0.394
a_R_NM	0.012	0.621	0.613	0.672	0.651	0.561	0.497
a_F_ML	−0.009	0.626	0.650	0.632	0.659	0.597	0.469
a_F_KS	0.024	0.651	0.629	0.648	0.700	0.595	0.472
a_F_CS	0.013	0.452	0.469	0.474	0.530	0.473	0.364
z_true	0.786	−0.004	−0.347	0.337	−0.020	−0.112	0.073
z_R_EZ	(*)	(*)	(*)	(*)	(*)	(*)	(*)
z_R_BY	0.790	−0.001	−0.353	0.349	−0.020	−0.121	0.081
z_R_BF	0.785	0.002	−0.348	0.350	−0.011	−0.108	0.082
z_R_NL	0.789	−0.001	−0.347	0.346	−0.019	−0.111	0.076
z_R_NM	0.787	0.002	−0.347	0.352	−0.011	−0.108	0.082
z_F_ML	0.786	−0.001	−0.350	0.349	−0.015	−0.111	0.079
z_F_KS	0.752	−0.017	−0.350	0.327	−0.027	−0.119	0.075
z_F_CS	0.741	−0.002	−0.328	0.325	−0.012	−0.098	0.079
t_true	−0.004	0.518	0.415	0.411	0.017	0.011	0.023
t_R_EZ	−0.007	0.320	0.159	0.139	−0.294	−0.225	−0.236
t_R_BY	−0.006	0.510	0.406	0.419	0.002	0.008	0.007
t_R_BF	−0.006	0.535	0.428	0.434	0.029	0.020	0.042
t_R_NL	−0.005	0.528	0.424	0.424	0.022	0.017	0.037
t_R_NM	−0.006	0.535	0.427	0.432	0.029	0.020	0.042
t_F_ML	−0.006	0.539	0.431	0.435	0.034	0.025	0.045
t_F_KS	−0.017	0.519	0.414	0.411	0.026	0.009	0.056
t_F_CS	−0.002	0.467	0.372	0.378	0.018	0.004	0.048
v_true	0.527	0.003	0.031	−0.010	0.021	0.028	0.010
v_R_EZ	0.883	−0.019	−0.172	0.145	−0.005	−0.040	0.031
v_R_BY	0.597	−0.018	0.019	−0.039	0.008	0.029	0.001
v_R_BF	0.504	−0.028	0.020	−0.057	0.002	0.020	−0.003
v_R_NL	0.500	−0.027	0.018	−0.051	0.010	0.022	0.002
v_R_NM	0.482	−0.037	0.016	−0.066	−0.007	0.014	−0.010
v_F_ML	0.498	−0.038	0.011	−0.065	−0.007	0.012	−0.010
v_F_KS	0.532	−0.029	−0.014	−0.028	−0.001	−0.006	0.004
v_F_CS	0.538	0.002	−0.001	0.015	0.020	0.014	0.019

Table 3c: Correlation coefficients of descriptive statistics of the sample with the true parameters and the parameter estimates (entire sample). For details see caption of Table 3a.

$n = 100$	prop_up	mean_rt	mean_up	mean_lo	var_rt	var_up	var_lo
a_true	−0.011	0.757	0.717	0.716	0.800	0.650	0.659
a_R_EZ	−0.019	0.705	0.717	0.708	0.800	0.682	0.692
a_R_BY	−0.027	0.746	0.724	0.716	0.825	0.661	0.692
a_R_BF	−0.024	0.747	0.728	0.718	0.810	0.663	0.680
a_R_NL	−0.028	0.736	0.726	0.707	0.795	0.663	0.659
a_R_NM	−0.026	0.746	0.728	0.716	0.808	0.663	0.678
a_F_ML	−0.026	0.740	0.728	0.711	0.801	0.664	0.670
a_F_KS	0.005	0.664	0.660	0.682	0.729	0.598	0.638
a_F_CS	−0.073	0.544	0.589	0.549	0.586	0.511	0.520
z_true	0.791	0.001	−0.376	0.368	−0.014	−0.158	0.125
z_R_EZ	(*)	(*)	(*)	(*)	(*)	(*)	(*)
z_R_BY	0.794	−0.002	−0.389	0.372	−0.018	−0.167	0.139
z_R_BF	0.792	−0.003	−0.386	0.371	−0.018	−0.161	0.127
z_R_NL	0.791	−0.002	−0.381	0.368	−0.016	−0.157	0.126
z_R_NM	0.792	−0.003	−0.384	0.369	−0.017	−0.161	0.126
z_F_ML	0.792	−0.002	−0.384	0.371	−0.017	−0.161	0.125
z_F_KS	0.785	0.003	−0.375	0.373	−0.011	−0.152	0.128
z_F_CS	0.799	0.001	−0.380	0.368	−0.015	−0.157	0.124
t_true	−0.001	0.520	0.396	0.425	0.008	−0.028	0.033
t_R_EZ	0.009	0.328	0.101	0.144	−0.269	−0.303	−0.218
t_R_BY	−0.003	0.514	0.389	0.415	−0.031	−0.055	0.006
t_R_BF	−0.001	0.534	0.406	0.435	0.019	−0.019	0.041
t_R_NL	0.001	0.529	0.402	0.431	0.014	−0.022	0.037
t_R_NM	0.001	0.532	0.405	0.433	0.018	−0.019	0.041
t_F_ML	0.001	0.535	0.407	0.436	0.022	−0.017	0.045
t_F_KS	−0.004	0.524	0.401	0.423	0.012	−0.019	0.036
t_F_CS	−0.001	0.520	0.397	0.424	0.009	−0.027	0.033
v_true	0.535	−0.004	0.002	−0.003	−0.003	0.014	−0.011
v_R_EZ	0.890	−0.007	−0.196	0.177	−0.009	−0.061	0.044
v_R_BY	0.556	−0.013	−0.006	−0.023	−0.012	0.006	−0.024
v_R_BF	0.502	−0.013	0.004	−0.029	−0.006	0.014	−0.025
v_R_NL	0.501	−0.009	0.007	−0.026	−0.000	0.018	−0.018
v_R_NM	0.471	−0.013	0.007	−0.033	−0.010	0.011	−0.027
v_F_ML	0.502	−0.011	0.005	−0.028	−0.005	0.014	−0.023
v_F_KS	0.529	−0.013	−0.021	−0.004	−0.006	0.003	−0.013
v_F_CS	0.468	−0.013	−0.009	−0.017	−0.000	0.010	−0.011

Table 3d: Correlation coefficients of descriptive statistics of the sample with the true parameters and the parameter estimates (entire sample). For details see caption of Table 3a.

$n = 400$	prop_up	mean_rt	mean_up	mean_lo	var_rt	var_up	var_lo
a_true	−0.002	0.766	0.737	0.739	0.850	0.750	0.774
a_R_EZ	−0.007	0.713	0.726	0.726	0.820	0.753	0.775
a_R_BY	−0.008	0.750	0.731	0.730	0.856	0.752	0.778
a_R_BF	−0.007	0.764	0.738	0.739	0.855	0.755	0.781
a_R_NL	−0.009	0.760	0.737	0.734	0.848	0.750	0.773
a_R_NM	−0.006	0.763	0.738	0.740	0.853	0.754	0.781
a_F_ML	−0.006	0.764	0.738	0.740	0.854	0.755	0.781
a_F_KS	−0.020	0.727	0.727	0.710	0.812	0.746	0.748
a_F_CS	−0.015	0.727	0.718	0.708	0.805	0.719	0.735
z_true	0.801	0.001	−0.379	0.377	−0.000	−0.168	0.172
z_R_EZ	(*)	(*)	(*)	(*)	(*)	(*)	(*)
z_R_BY	0.802	0.006	−0.375	0.377	0.005	−0.161	0.168
z_R_BF	0.800	0.002	−0.380	0.379	0.002	−0.166	0.174
z_R_NL	0.800	0.002	−0.374	0.372	−0.003	−0.166	0.166
z_R_NM	0.801	0.003	−0.378	0.378	0.003	−0.164	0.174
z_F_ML	0.800	0.002	−0.379	0.379	0.002	−0.165	0.175
z_F_KS	0.801	−0.000	−0.382	0.374	−0.001	−0.169	0.172
z_F_CS	0.799	0.000	−0.380	0.378	0.002	−0.163	0.175
t_true	0.001	0.522	0.433	0.427	−0.002	0.019	−0.016
t_R_EZ	0.002	0.342	0.156	0.153	−0.241	−0.233	−0.262
t_R_BY	0.003	0.531	0.436	0.436	−0.030	−0.007	−0.034
t_R_BF	0.001	0.522	0.432	0.427	−0.004	0.017	−0.017
t_R_NL	0.002	0.517	0.427	0.424	−0.007	0.014	−0.020
t_R_NM	0.002	0.523	0.434	0.429	−0.001	0.019	−0.014
t_F_ML	0.001	0.523	0.434	0.428	−0.002	0.019	−0.016
t_F_KS	0.005	0.522	0.431	0.428	−0.001	0.016	−0.014
t_F_CS	0.003	0.519	0.430	0.426	−0.005	0.014	−0.018
v_true	0.538	−0.002	0.003	−0.004	−0.002	0.002	0.003
v_R_EZ	0.895	−0.001	−0.195	0.192	0.000	−0.071	0.078
v_R_BY	0.538	−0.007	−0.003	−0.008	−0.007	−0.006	0.002
v_R_BF	0.521	−0.004	0.006	−0.013	−0.006	−0.002	−0.003
v_R_NL	0.521	−0.001	0.003	−0.006	0.002	0.001	0.006
v_R_NM	0.490	−0.011	0.003	−0.022	−0.014	−0.009	−0.010
v_F_ML	0.520	−0.004	0.006	−0.012	−0.006	−0.002	−0.002
v_F_KS	0.530	−0.006	−0.004	−0.004	−0.004	−0.006	0.003
v_F_CS	0.502	−0.000	0.012	−0.016	−0.005	−0.006	−0.004

4 Box Plots of Parameter Estimates

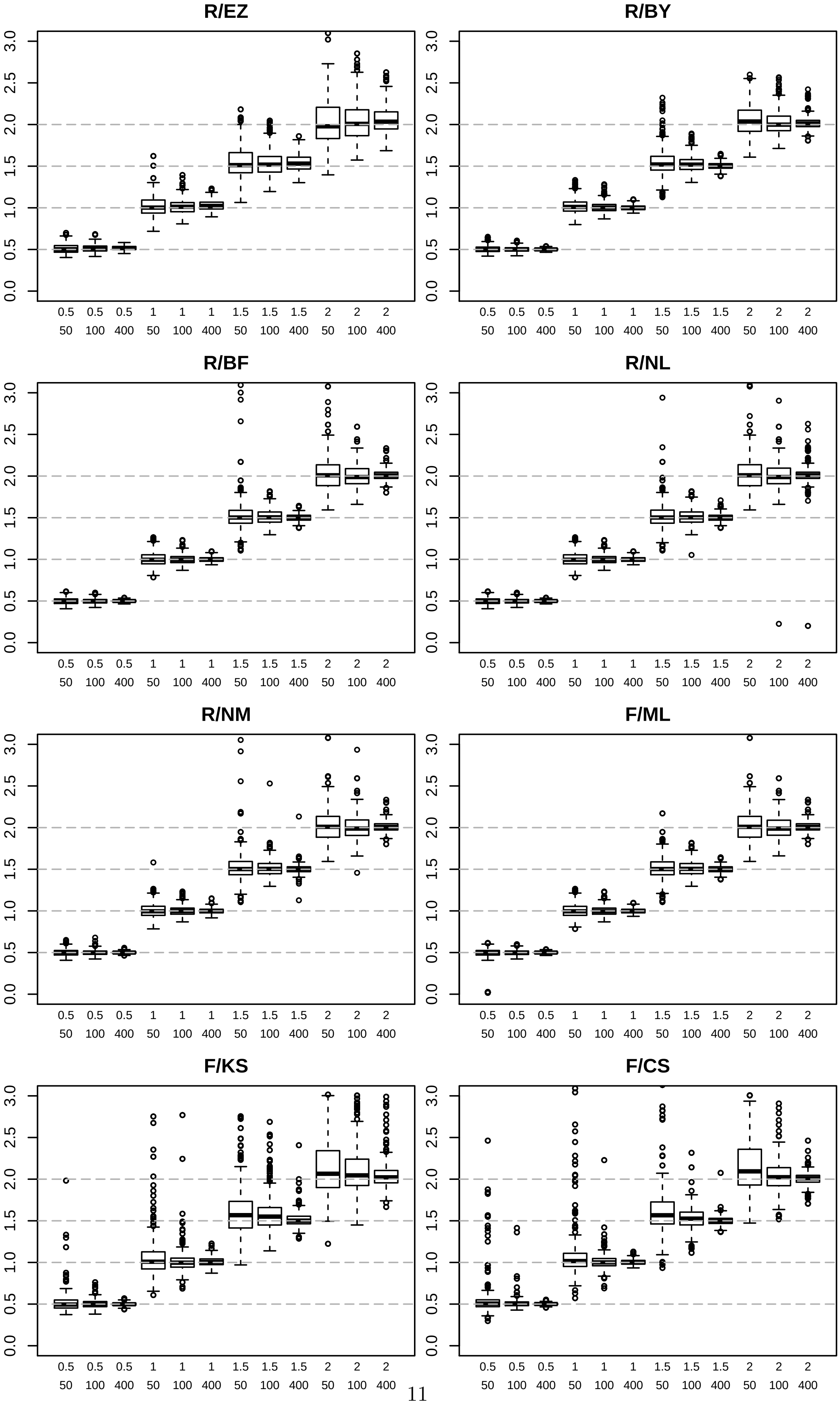

Figure 2a: Box plots of the estimates of boundary separation a.

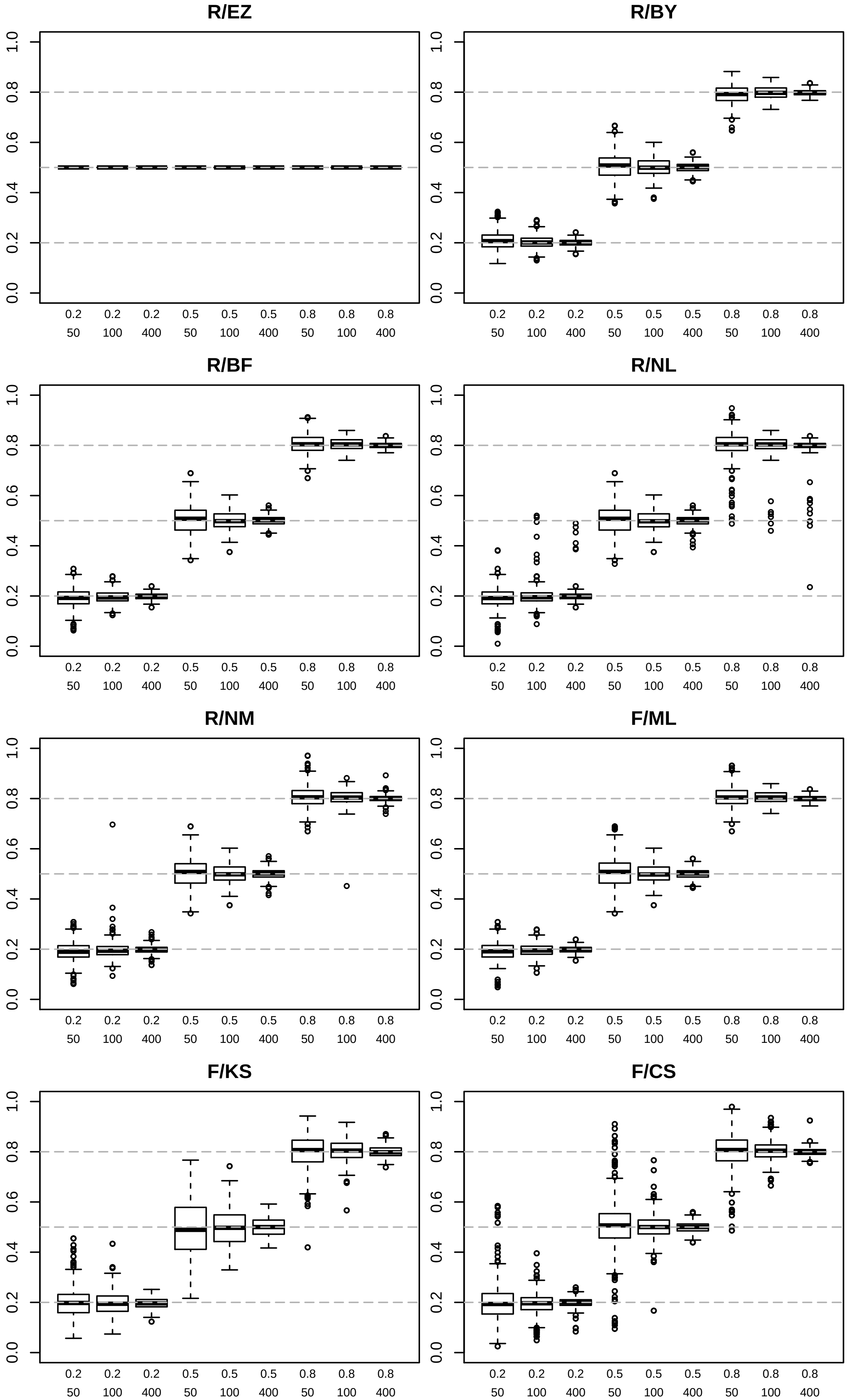

Figure 2b: Box plots of the estimates of the bias parameter z. Note: EZ cannot estimate z, but rather sets the value to 0.5.

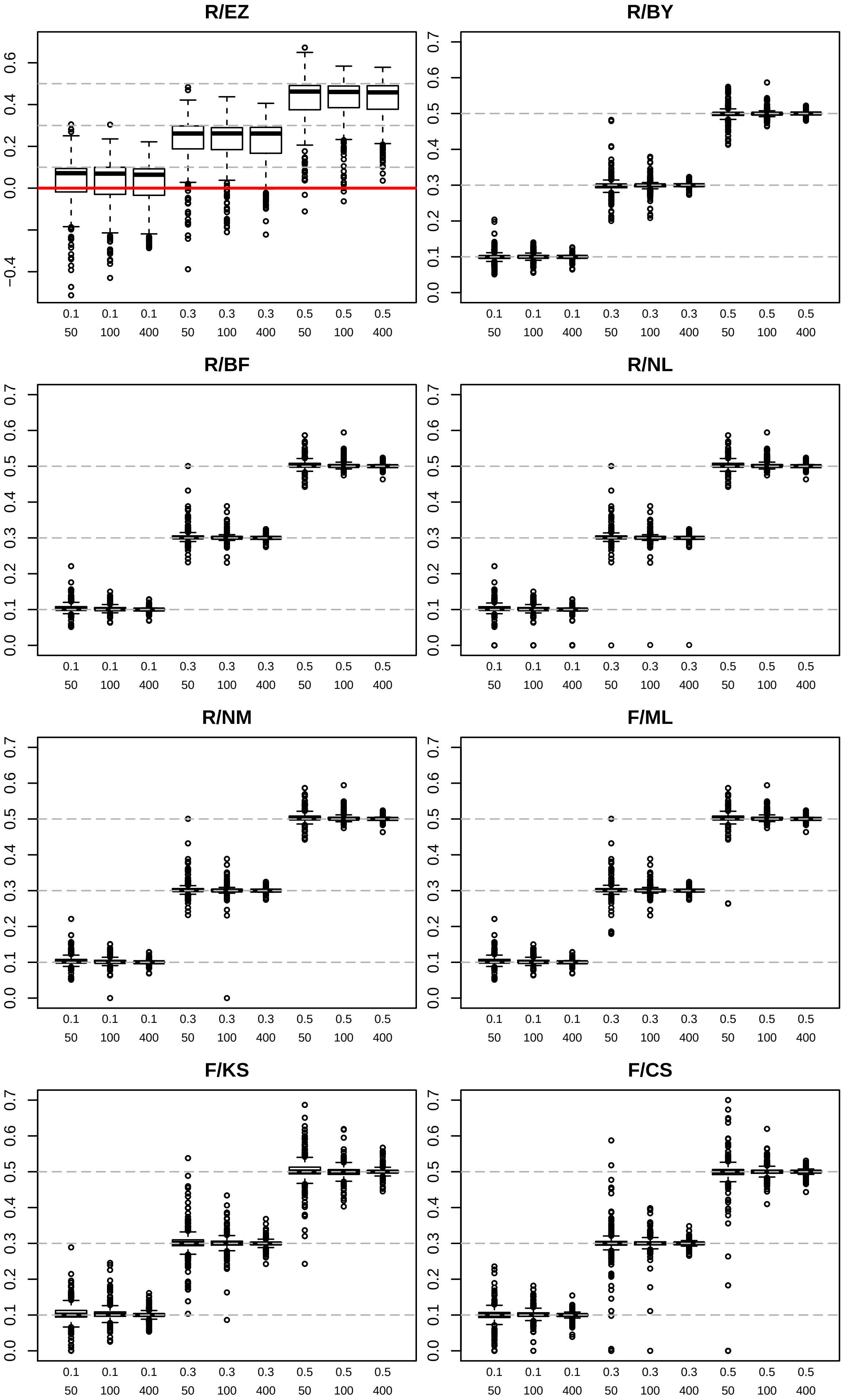

Figure 2c: Box plots of the estimates of the encoding and response time parameter T_{ER}. Note: The bold (red) line in the upper left diagram indicates the zero-line. Estimates below this line represent model violations.

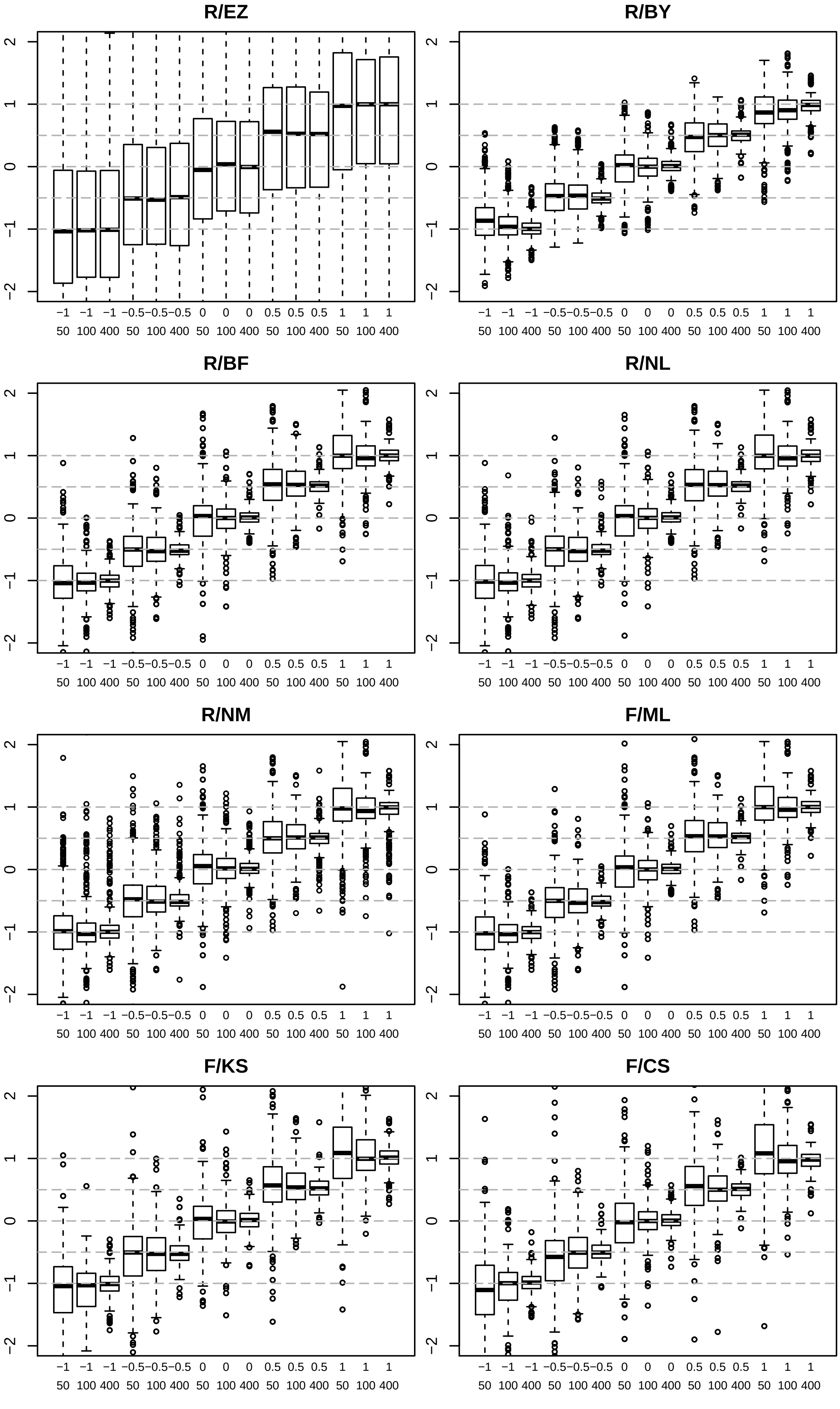

Figure 2d: Box plots of the estimates of the drift parameter ν.

5 Correlation Coefficients and Scatter Plots of Parameter Estimates

5.1 Correlation Coefficients of True and Estimated Parameters

Table 4: Correlation coefficients of true and estimated parameters by number of trials. Notes: Par. = parameter; tot = total sample, 50/100/400 = number of trials; (*) No correlation can be obtained for the z parameter with EZ

	R-EZ	R-BY	R-BF	R-NL	R-NM	F-ML	F-KS	F-CS
a tot	0.965	0.985	0.961	0.867	0.924	0.927	0.929	0.758
a 50	0.950	0.974	0.915	0.742	0.838	0.850	0.892	0.673
a 100	0.965	0.987	0.984	0.975	0.983	0.978	0.931	0.794
a 400	0.981	0.997	0.997	0.992	0.997	0.997	0.978	0.958
z tot	(*)	0.992	0.992	0.989	0.991	0.991	0.972	0.970
z 50	(*)	0.985	0.985	0.982	0.984	0.983	0.948	0.932
z 100	(*)	0.993	0.993	0.990	0.991	0.993	0.975	0.983
z 400	(*)	0.998	0.998	0.994	0.998	0.998	0.994	0.997
t tot	0.811	0.997	0.997	0.995	0.997	0.996	0.987	0.977
t 50	0.788	0.994	0.994	0.992	0.994	0.991	0.974	0.942
t 100	0.809	0.998	0.998	0.996	0.996	0.998	0.989	0.992
t 400	0.838	1.000	0.999	0.997	0.999	0.999	0.997	0.999
v tot	0.441	0.913	0.889	0.886	0.857	0.886	0.823	0.785
v 50	0.432	0.855	0.820	0.816	0.800	0.814	0.728	0.677
v 100	0.443	0.911	0.897	0.895	0.859	0.897	0.858	0.860
v 400	0.450	0.975	0.974	0.971	0.932	0.974	0.961	0.968

5.2 Correlation Coefficients of Estimates—Entire Sample

Table 5a: Correlation coefficients of estimated parameters across programs: The Boundary Separation *a*.

	a_R_EZ	a_R_BY	a_R_BF	a_R_NL	a_R_NM	a_F_ML	a_F_KS	a_F_CS
a_R_EZ	1.000	0.979	0.962	0.872	0.926	0.933	0.953	0.794
a_R_BY	0.979	1.000	0.983	0.892	0.948	0.955	0.949	0.784
a_R_BF	0.962	0.983	1.000	0.928	0.973	0.992	0.934	0.787
a_R_NL	0.872	0.892	0.928	1.000	0.909	0.935	0.852	0.722
a_R_NM	0.926	0.948	0.973	0.909	1.000	0.972	0.900	0.767
a_F_ML	0.933	0.955	0.992	0.935	0.972	1.000	0.908	0.776
a_F_KS	0.953	0.949	0.934	0.852	0.900	0.908	1.000	0.799
a_F_CS	0.794	0.784	0.787	0.722	0.767	0.776	0.799	1.000
z_R_EZ	(*)	(*)	(*)	(*)	(*)	(*)	(*)	(*)
z_R_BY	-0.010	-0.006	-0.008	-0.015	-0.002	-0.010	0.001	-0.022
z_R_BF	-0.006	-0.006	-0.006	-0.014	0.001	-0.007	0.005	-0.018
z_R_NL	-0.009	-0.009	-0.009	-0.017	-0.002	-0.011	0.002	-0.019
z_R_NM	-0.005	-0.005	-0.004	-0.013	0.003	-0.006	0.006	-0.016
z_F_ML	-0.007	-0.007	-0.007	-0.016	0.000	-0.010	0.004	-0.018
z_F_KS	-0.007	-0.007	-0.006	-0.013	0.000	-0.007	0.006	-0.022
z_F_CS	-0.007	-0.005	-0.003	-0.009	0.005	-0.003	0.003	-0.023
t_R_EZ	-0.359	-0.283	-0.297	-0.277	-0.286	-0.291	-0.317	-0.285
t_R_BY	-0.010	-0.013	-0.008	-0.012	-0.006	-0.006	-0.008	-0.005
t_R_BF	0.013	0.003	0.014	0.008	0.015	0.015	0.012	0.009
t_R_NL	0.009	-0.002	0.010	0.004	0.011	0.011	0.007	0.005
t_R_NM	0.013	0.002	0.013	0.008	0.015	0.015	0.011	0.009
t_F_ML	0.017	0.006	0.017	0.011	0.018	0.020	0.015	0.012
t_F_KS	0.004	-0.005	0.007	0.002	0.008	0.009	-0.007	0.004
t_F_CS	-0.001	-0.017	-0.004	-0.008	-0.002	-0.001	-0.007	-0.019
v_R_EZ	-0.013	-0.016	-0.015	-0.019	-0.010	-0.015	-0.000	-0.016
v_R_BY	-0.020	-0.021	-0.022	-0.026	-0.016	-0.022	-0.007	-0.022
v_R_BF	-0.021	-0.026	-0.024	-0.027	-0.018	-0.023	-0.008	-0.019
v_R_NL	-0.016	-0.020	-0.018	-0.022	-0.013	-0.017	-0.003	-0.014
v_R_NM	-0.030	-0.035	-0.033	-0.035	-0.026	-0.032	-0.018	-0.026
v_F_ML	-0.028	-0.032	-0.030	-0.032	-0.024	-0.032	-0.014	-0.024
v_F_KS	-0.023	-0.028	-0.024	-0.025	-0.019	-0.023	-0.014	-0.022
v_F_CS	-0.004	-0.009	-0.007	-0.011	-0.004	-0.007	0.009	-0.007

Table 5b: Correlation coefficients of estimated parameters across programs: The Starting Point / Bias z. Note: Asterisks (*) indicate cases, in which no correlation can be obtained because the EZ method sets z to a constant value of 0.5.

	z_R_EZ	z_R_BY	z_R_BF	z_R_NL	z_R_NM	z_F_ML	z_F_KS	z_F_CS
a_R_EZ	(*)	-0.010	-0.006	-0.009	-0.005	-0.007	-0.007	-0.007
a_R_BY	(*)	-0.006	-0.006	-0.009	-0.005	-0.007	-0.007	-0.005
a_R_BF	(*)	-0.008	-0.006	-0.009	-0.004	-0.007	-0.006	-0.003
a_R_NL	(*)	-0.015	-0.014	-0.017	-0.013	-0.016	-0.013	-0.009
a_R_NM	(*)	-0.002	0.001	-0.002	0.003	0.000	0.000	0.005
a_F_ML	(*)	-0.010	-0.007	-0.011	-0.006	-0.010	-0.007	-0.003
a_F_KS	(*)	0.001	0.005	0.002	0.006	0.004	0.006	0.003
a_F_CS	(*)	-0.022	-0.018	-0.019	-0.016	-0.018	-0.022	-0.023
z_R_EZ	(*)	(*)	(*)	(*)	(*)	(*)	(*)	(*)
z_R_BY	(*)	1.000	0.999	0.997	0.999	0.999	0.979	0.977
z_R_BF	(*)	0.999	1.000	0.997	0.999	1.000	0.979	0.977
z_R_NL	(*)	0.997	0.997	1.000	0.997	0.997	0.976	0.974
z_R_NM	(*)	0.999	0.999	0.997	1.000	0.999	0.978	0.976
z_F_ML	(*)	0.999	1.000	0.997	0.999	1.000	0.978	0.976
z_F_KS	(*)	0.979	0.979	0.976	0.978	0.978	1.000	0.976
z_F_CS	(*)	0.977	0.977	0.974	0.976	0.976	0.976	1.000
t_R_EZ	(*)	0.013	0.008	0.013	0.008	0.009	0.006	0.008
t_R_BY	(*)	0.001	0.000	0.004	0.000	0.001	-0.002	-0.002
t_R_BF	(*)	0.000	-0.000	0.003	-0.001	0.000	-0.003	-0.003
t_R_NL	(*)	0.001	-0.000	0.004	-0.000	0.001	-0.002	-0.002
t_R_NM	(*)	0.001	-0.000	0.004	-0.000	0.001	-0.003	-0.003
t_F_ML	(*)	0.001	-0.000	0.004	-0.000	-0.000	-0.003	-0.002
t_F_KS	(*)	-0.002	-0.004	0.000	-0.004	-0.003	-0.007	-0.006
t_F_CS	(*)	0.001	0.000	0.004	0.000	0.001	-0.002	-0.002
v_R_EZ	(*)	0.772	0.764	0.766	0.766	0.764	0.747	0.750
v_R_BY	(*)	0.010	0.002	0.007	0.008	0.004	0.011	0.013
v_R_BF	(*)	-0.027	-0.037	-0.033	-0.031	-0.036	-0.028	-0.024
v_R_NL	(*)	-0.024	-0.034	-0.034	-0.028	-0.033	-0.025	-0.022
v_R_NM	(*)	-0.050	-0.059	-0.054	-0.061	-0.057	-0.048	-0.046
v_F_ML	(*)	-0.027	-0.037	-0.033	-0.032	-0.034	-0.028	-0.025
v_F_KS	(*)	0.050	0.040	0.044	0.046	0.041	0.013	0.038
v_F_CS	(*)	0.037	0.028	0.032	0.034	0.029	0.007	-0.006

Table 5c: Correlation coefficients of estimated parameters across programs: The encoding and reaction time T_{ER}.

	t_R_EZ	t_R_BY	t_R_BF	t_R_NL	t_R_NM	t_F_ML	t_F_KS	t_F_CS
a_R_EZ	-0.359	-0.010	0.013	0.009	0.013	0.017	0.004	-0.001
a_R_BY	-0.283	-0.013	0.003	-0.002	0.002	0.006	-0.005	-0.017
a_R_BF	-0.297	-0.008	0.014	0.010	0.013	0.017	0.007	-0.004
a_R_NL	-0.277	-0.012	0.008	0.004	0.008	0.011	0.002	-0.008
a_R_NM	-0.286	-0.006	0.015	0.011	0.015	0.018	0.008	-0.002
a_F_ML	-0.291	-0.006	0.015	0.011	0.015	0.020	0.009	-0.001
a_F_KS	-0.317	-0.008	0.012	0.007	0.011	0.015	-0.007	-0.007
a_F_CS	-0.285	-0.005	0.009	0.005	0.009	0.012	0.004	-0.019
z_R_EZ	(*)	(*)	(*)	(*)	(*)	(*)	(*)	(*)
z_R_BY	0.013	0.001	0.000	0.001	0.001	0.001	-0.002	0.001
z_R_BF	0.008	0.000	-0.000	-0.000	-0.000	-0.000	-0.004	0.000
z_R_NL	0.013	0.004	0.003	0.004	0.004	0.004	0.000	0.004
z_R_NM	0.008	0.000	-0.001	-0.000	-0.000	-0.000	-0.004	0.000
z_F_ML	0.009	0.001	0.000	0.001	0.001	-0.000	-0.003	0.001
z_F_KS	0.006	-0.002	-0.003	-0.002	-0.003	-0.003	-0.007	-0.002
z_F_CS	0.008	-0.002	-0.003	-0.002	-0.003	-0.002	-0.006	-0.002
t_R_EZ	1.000	0.828	0.812	0.813	0.813	0.811	0.809	0.787
t_R_BY	0.828	1.000	1.000	0.998	0.999	0.998	0.989	0.979
t_R_BF	0.812	1.000	1.000	0.999	1.000	0.999	0.989	0.978
t_R_NL	0.813	0.998	0.999	1.000	0.998	0.997	0.987	0.977
t_R_NM	0.813	0.999	1.000	0.998	1.000	0.998	0.989	0.978
t_F_ML	0.811	0.998	0.999	0.997	0.998	1.000	0.988	0.977
t_F_KS	0.809	0.989	0.989	0.987	0.989	0.988	1.000	0.979
t_F_CS	0.787	0.979	0.978	0.977	0.978	0.977	0.979	1.000
v_R_EZ	-0.001	-0.006	-0.006	-0.005	-0.006	-0.006	-0.008	-0.005
v_R_BY	-0.002	-0.005	-0.005	-0.004	-0.005	-0.004	-0.007	-0.003
v_R_BF	-0.005	-0.008	-0.007	-0.006	-0.007	-0.005	-0.008	-0.005
v_R_NL	-0.011	-0.014	-0.012	-0.014	-0.013	-0.011	-0.014	-0.011
v_R_NM	0.001	-0.003	-0.001	-0.001	-0.002	-0.001	-0.004	-0.000
v_F_ML	-0.001	-0.006	-0.005	-0.004	-0.005	-0.009	-0.007	-0.003
v_F_KS	-0.005	-0.008	-0.007	-0.007	-0.007	-0.007	-0.009	-0.006
v_F_CS	-0.008	-0.007	-0.006	-0.006	-0.006	-0.006	-0.009	-0.006

Table 5d: Correlation coefficients of estimated parameters across programs: The drift parameter ν.

	v_R_EZ	v_R_BY	v_R_BF	v_R_NL	v_R_NM	v_F_ML	v_F_KS	v_F_CS
a_R_EZ	-0.013	-0.020	-0.021	-0.016	-0.030	-0.028	-0.023	-0.004
a_R_BY	-0.016	-0.021	-0.026	-0.020	-0.035	-0.032	-0.028	-0.009
a_R_BF	-0.015	-0.022	-0.024	-0.018	-0.033	-0.030	-0.024	-0.007
a_R_NL	-0.019	-0.026	-0.027	-0.022	-0.035	-0.032	-0.025	-0.011
a_R_NM	-0.010	-0.016	-0.018	-0.013	-0.026	-0.024	-0.019	-0.004
a_F_ML	-0.015	-0.022	-0.023	-0.017	-0.032	-0.032	-0.023	-0.007
a_F_KS	-0.000	-0.007	-0.008	-0.003	-0.018	-0.014	-0.014	0.009
a_F_CS	-0.016	-0.022	-0.019	-0.014	-0.026	-0.024	-0.022	-0.007
z_R_EZ	(*)	(*)	(*)	(*)	(*)	(*)	(*)	(*)
z_R_BY	0.772	0.010	-0.027	-0.024	-0.050	-0.027	0.050	0.037
z_R_BF	0.764	0.002	-0.037	-0.034	-0.059	-0.037	0.040	0.028
z_R_NL	0.766	0.007	-0.033	-0.034	-0.054	-0.033	0.044	0.032
z_R_NM	0.766	0.008	-0.031	-0.028	-0.061	-0.032	0.046	0.034
z_F_ML	0.764	0.004	-0.036	-0.033	-0.057	-0.034	0.041	0.029
z_F_KS	0.747	0.011	-0.028	-0.025	-0.048	-0.028	0.013	0.007
z_F_CS	0.750	0.013	-0.024	-0.022	-0.046	-0.025	0.038	-0.006
t_R_EZ	-0.001	-0.002	-0.005	-0.011	0.001	-0.001	-0.005	-0.008
t_R_BY	-0.006	-0.005	-0.008	-0.014	-0.003	-0.006	-0.008	-0.007
t_R_BF	-0.006	-0.005	-0.007	-0.012	-0.001	-0.005	-0.007	-0.006
t_R_NL	-0.005	-0.004	-0.006	-0.014	-0.001	-0.004	-0.007	-0.006
t_R_NM	-0.006	-0.005	-0.007	-0.013	-0.002	-0.005	-0.007	-0.006
t_F_ML	-0.006	-0.004	-0.005	-0.011	-0.001	-0.009	-0.007	-0.006
t_F_KS	-0.008	-0.007	-0.008	-0.014	-0.004	-0.007	-0.009	-0.009
t_F_CS	-0.005	-0.003	-0.005	-0.011	-0.000	-0.003	-0.006	-0.006
v_R_EZ	1.000	0.483	0.453	0.451	0.403	0.449	0.499	0.466
v_R_BY	0.483	1.000	0.986	0.982	0.945	0.981	0.919	0.872
v_R_BF	0.453	0.986	1.000	0.997	0.950	0.995	0.940	0.886
v_R_NL	0.451	0.982	0.997	1.000	0.947	0.992	0.937	0.885
v_R_NM	0.403	0.945	0.950	0.947	1.000	0.947	0.885	0.833
v_F_ML	0.449	0.981	0.995	0.992	0.947	1.000	0.936	0.883
v_F_KS	0.499	0.919	0.940	0.937	0.885	0.936	1.000	0.880
v_F_CS	0.466	0.872	0.886	0.885	0.833	0.883	0.880	1.000

5.3 Correlation Coefficients of Estimates—50 Trials

Table 6a: Correlation coefficients of estimated parameters across programs: The Boundary Separation a.

	a_R_EZ	a_R_BY	a_R_BF	a_R_NL	a_R_NM	a_F_ML	a_F_KS	a_F_CS
a_R_EZ	1.000	0.973	0.933	0.765	0.856	0.878	0.929	0.741
a_R_BY	0.973	1.000	0.961	0.789	0.885	0.905	0.923	0.716
a_R_BF	0.933	0.961	1.000	0.857	0.940	0.985	0.888	0.715
a_R_NL	0.765	0.789	0.857	1.000	0.817	0.872	0.737	0.592
a_R_NM	0.856	0.885	0.940	0.817	1.000	0.940	0.816	0.672
a_F_ML	0.878	0.905	0.985	0.872	0.940	1.000	0.838	0.690
a_F_KS	0.929	0.923	0.888	0.737	0.816	0.838	1.000	0.743
a_F_CS	0.741	0.716	0.715	0.592	0.672	0.690	0.743	1.000
z_R_EZ	(*)	(*)	(*)	(*)	(*)	(*)	(*)	(*)
z_R_BY	-0.015	-0.009	-0.014	-0.026	0.003	-0.015	0.003	-0.012
z_R_BF	-0.006	-0.007	-0.007	-0.022	0.011	-0.009	0.013	-0.002
z_R_NL	-0.011	-0.013	-0.012	-0.029	0.007	-0.014	0.009	-0.004
z_R_NM	-0.004	-0.005	-0.004	-0.020	0.016	-0.006	0.016	0.001
z_F_ML	-0.011	-0.012	-0.011	-0.026	0.008	-0.015	0.009	-0.004
z_F_KS	-0.013	-0.016	-0.012	-0.024	0.005	-0.012	0.010	-0.009
z_F_CS	-0.005	-0.004	0.001	-0.010	0.021	0.002	0.010	0.002
t_R_EZ	-0.364	-0.263	-0.286	-0.258	-0.267	-0.281	-0.309	-0.305
t_R_BY	0.000	-0.001	0.012	-0.002	0.012	0.015	0.001	-0.005
t_R_BF	0.025	0.027	0.035	0.016	0.034	0.036	0.022	0.004
t_R_NL	0.018	0.019	0.029	0.011	0.028	0.030	0.016	0.000
t_R_NM	0.024	0.026	0.035	0.016	0.033	0.036	0.022	0.005
t_F_ML	0.034	0.035	0.043	0.023	0.041	0.047	0.030	0.011
t_F_KS	0.011	0.014	0.025	0.009	0.024	0.026	-0.007	-0.003
t_F_CS	-0.006	-0.017	-0.001	-0.012	0.001	0.003	-0.014	-0.047
v_R_EZ	-0.016	-0.018	-0.017	-0.025	-0.005	-0.016	0.013	0.007
v_R_BY	-0.009	-0.010	-0.012	-0.022	0.004	-0.010	0.017	0.018
v_R_BF	-0.018	-0.021	-0.020	-0.026	-0.006	-0.017	0.009	0.016
v_R_NL	-0.012	-0.014	-0.014	-0.021	-0.001	-0.012	0.016	0.019
v_R_NM	-0.030	-0.034	-0.032	-0.036	-0.016	-0.029	-0.005	0.004
v_F_ML	-0.037	-0.039	-0.036	-0.039	-0.021	-0.040	-0.008	0.002
v_F_KS	-0.029	-0.030	-0.026	-0.027	-0.014	-0.022	-0.011	0.006
v_F_CS	0.012	0.009	0.010	0.000	0.016	0.009	0.041	0.030

Table 6b: Correlation coefficients of estimated parameters across programs: The Starting Point / Bias z

	z_R_EZ	z_R_BY	z_R_BF	z_R_NL	z_R_NM	z_F_ML	z_F_KS	z_F_CS
a_R_EZ	(*)	-0.015	-0.006	-0.011	-0.004	-0.011	-0.013	-0.005
a_R_BY	(*)	-0.009	-0.007	-0.013	-0.005	-0.012	-0.016	-0.004
a_R_BF	(*)	-0.014	-0.007	-0.012	-0.004	-0.011	-0.012	0.001
a_R_NL	(*)	-0.026	-0.022	-0.029	-0.020	-0.026	-0.024	-0.010
a_R_NM	(*)	0.003	0.011	0.007	0.016	0.008	0.005	0.021
a_F_ML	(*)	-0.015	-0.009	-0.014	-0.006	-0.015	-0.012	0.002
a_F_KS	(*)	0.003	0.013	0.009	0.016	0.009	0.010	0.010
a_F_CS	(*)	-0.012	-0.002	-0.004	0.001	-0.004	-0.009	0.002
z_R_EZ	(*)	(*)	(*)	(*)	(*)	(*)	(*)	(*)
z_R_BY	(*)	1.000	0.999	0.997	0.999	0.998	0.961	0.947
z_R_BF	(*)	0.999	1.000	0.998	0.999	0.999	0.960	0.945
z_R_NL	(*)	0.997	0.998	1.000	0.997	0.997	0.958	0.944
z_R_NM	(*)	0.999	0.999	0.997	1.000	0.999	0.959	0.945
z_F_ML	(*)	0.998	0.999	0.997	0.999	1.000	0.959	0.944
z_F_KS	(*)	0.961	0.960	0.958	0.959	0.959	1.000	0.956
z_F_CS	(*)	0.947	0.945	0.944	0.945	0.944	0.956	1.000
t_R_EZ	(*)	0.020	0.012	0.017	0.011	0.014	0.003	0.006
t_R_BY	(*)	0.001	0.001	0.006	0.000	0.002	-0.007	-0.006
t_R_BF	(*)	0.001	0.001	0.006	0.000	0.002	-0.007	-0.007
t_R_NL	(*)	-0.001	-0.001	0.006	-0.001	-0.000	-0.008	-0.008
t_R_NM	(*)	0.001	0.001	0.006	0.001	0.002	-0.007	-0.006
t_F_ML	(*)	0.000	0.000	0.005	0.000	-0.002	-0.007	-0.006
t_F_KS	(*)	-0.006	-0.007	-0.002	-0.007	-0.006	-0.014	-0.012
t_F_CS	(*)	0.001	0.001	0.006	0.001	0.002	-0.005	-0.002
v_R_EZ	(*)	0.759	0.747	0.750	0.750	0.747	0.714	0.705
v_R_BY	(*)	0.035	0.023	0.029	0.029	0.025	0.022	0.025
v_R_BF	(*)	-0.027	-0.042	-0.037	-0.035	-0.040	-0.037	-0.033
v_R_NL	(*)	-0.025	-0.039	-0.039	-0.033	-0.038	-0.036	-0.031
v_R_NM	(*)	-0.049	-0.061	-0.057	-0.063	-0.060	-0.057	-0.052
v_F_ML	(*)	-0.027	-0.041	-0.037	-0.036	-0.035	-0.039	-0.034
v_F_KS	(*)	0.091	0.076	0.080	0.082	0.077	0.024	0.055
v_F_CS	(*)	0.136	0.123	0.126	0.129	0.123	0.068	0.032

Table 6c: Correlation coefficients of estimated parameters across programs: The encoding and reaction time T_{ER}.

	t_R_EZ	t_R_BY	t_R_BF	t_R_NL	t_R_NM	t_F_ML	t_F_KS	t_F_CS
a_R_EZ	-0.364	0.000	0.025	0.018	0.024	0.034	0.011	-0.006
a_R_BY	-0.263	-0.001	0.027	0.019	0.026	0.035	0.014	-0.017
a_R_BF	-0.286	0.012	0.035	0.029	0.035	0.043	0.025	-0.001
a_R_NL	-0.258	-0.002	0.016	0.011	0.016	0.023	0.009	-0.012
a_R_NM	-0.267	0.012	0.034	0.028	0.033	0.041	0.024	0.001
a_F_ML	-0.281	0.015	0.036	0.030	0.036	0.047	0.026	0.003
a_F_KS	-0.309	0.001	0.022	0.016	0.022	0.030	-0.007	-0.014
a_F_CS	-0.305	-0.005	0.004	0.000	0.005	0.011	-0.003	-0.047
z_R_EZ	(*)	(*)	(*)	(*)	(*)	(*)	(*)	(*)
z_R_BY	0.020	0.001	0.001	-0.001	0.001	0.000	-0.006	0.001
z_R_BF	0.012	0.001	0.001	-0.001	0.001	0.000	-0.007	0.001
z_R_NL	0.017	0.006	0.006	0.006	0.006	0.005	-0.002	0.006
z_R_NM	0.011	0.000	0.000	-0.001	0.001	0.000	-0.007	0.001
z_F_ML	0.014	0.002	0.002	-0.000	0.002	-0.002	-0.006	0.002
z_F_KS	0.003	-0.007	-0.007	-0.008	-0.007	-0.007	-0.014	-0.005
z_F_CS	0.006	-0.006	-0.007	-0.008	-0.006	-0.006	-0.012	-0.002
t_R_EZ	1.000	0.806	0.791	0.791	0.791	0.786	0.778	0.721
t_R_BY	0.806	1.000	0.999	0.998	0.999	0.996	0.980	0.946
t_R_BF	0.791	0.999	1.000	0.998	1.000	0.997	0.979	0.943
t_R_NL	0.791	0.998	0.998	1.000	0.998	0.995	0.976	0.943
t_R_NM	0.791	0.999	1.000	0.998	1.000	0.997	0.979	0.943
t_F_ML	0.786	0.996	0.997	0.995	0.997	1.000	0.975	0.940
t_F_KS	0.778	0.980	0.979	0.976	0.979	0.975	1.000	0.949
t_F_CS	0.721	0.946	0.943	0.943	0.943	0.940	0.949	1.000
v_R_EZ	-0.012	-0.017	-0.017	-0.017	-0.018	-0.016	-0.022	-0.014
v_R_BY	-0.024	-0.023	-0.021	-0.019	-0.023	-0.020	-0.025	-0.016
v_R_BF	-0.026	-0.027	-0.028	-0.025	-0.028	-0.024	-0.030	-0.022
v_R_NL	-0.035	-0.036	-0.035	-0.037	-0.037	-0.032	-0.038	-0.030
v_R_NM	-0.021	-0.023	-0.022	-0.021	-0.024	-0.022	-0.026	-0.018
v_F_ML	-0.018	-0.022	-0.022	-0.020	-0.024	-0.032	-0.025	-0.017
v_F_KS	-0.016	-0.022	-0.022	-0.021	-0.024	-0.021	-0.026	-0.018
v_F_CS	-0.020	-0.014	-0.013	-0.012	-0.015	-0.013	-0.018	-0.013

Table 6d: Correlation coefficients of estimated parameters across programs: The drift parameter ν.

	v_R_EZ	v_R_BY	v_R_BF	v_R_NL	v_R_NM	v_F_ML	v_F_KS	v_F_CS
a_R_EZ	-0.016	-0.009	-0.018	-0.012	-0.030	-0.037	-0.029	0.012
a_R_BY	-0.018	-0.010	-0.021	-0.014	-0.034	-0.039	-0.030	0.009
a_R_BF	-0.017	-0.012	-0.020	-0.014	-0.032	-0.036	-0.026	0.010
a_R_NL	-0.025	-0.022	-0.026	-0.021	-0.036	-0.039	-0.027	0.000
a_R_NM	-0.005	0.004	-0.006	-0.001	-0.016	-0.021	-0.014	0.016
a_F_ML	-0.016	-0.010	-0.017	-0.012	-0.029	-0.040	-0.022	0.009
a_F_KS	0.013	0.017	0.009	0.016	-0.005	-0.008	-0.011	0.041
a_F_CS	0.007	0.018	0.016	0.019	0.004	0.002	0.006	0.030
z_R_EZ	(*)	(*)	(*)	(*)	(*)	(*)	(*)	(*)
z_R_BY	0.759	0.035	-0.027	-0.025	-0.049	-0.027	0.091	0.136
z_R_BF	0.747	0.023	-0.042	-0.039	-0.061	-0.041	0.076	0.123
z_R_NL	0.750	0.029	-0.037	-0.039	-0.057	-0.037	0.080	0.126
z_R_NM	0.750	0.029	-0.035	-0.033	-0.063	-0.036	0.082	0.129
z_F_ML	0.747	0.025	-0.040	-0.038	-0.060	-0.035	0.077	0.123
z_F_KS	0.714	0.022	-0.037	-0.036	-0.057	-0.039	0.024	0.068
z_F_CS	0.705	0.025	-0.033	-0.031	-0.052	-0.034	0.055	0.032
t_R_EZ	-0.012	-0.024	-0.026	-0.035	-0.021	-0.018	-0.016	-0.020
t_R_BY	-0.017	-0.023	-0.027	-0.036	-0.023	-0.022	-0.022	-0.014
t_R_BF	-0.017	-0.021	-0.028	-0.035	-0.022	-0.022	-0.022	-0.013
t_R_NL	-0.017	-0.019	-0.025	-0.037	-0.021	-0.020	-0.021	-0.012
t_R_NM	-0.018	-0.023	-0.028	-0.037	-0.024	-0.024	-0.024	-0.015
t_F_ML	-0.016	-0.020	-0.024	-0.032	-0.022	-0.032	-0.021	-0.013
t_F_KS	-0.022	-0.025	-0.030	-0.038	-0.026	-0.025	-0.026	-0.018
t_F_CS	-0.014	-0.016	-0.022	-0.030	-0.018	-0.017	-0.018	-0.013
v_R_EZ	1.000	0.520	0.471	0.468	0.423	0.463	0.552	0.566
v_R_BY	0.520	1.000	0.976	0.972	0.948	0.965	0.883	0.806
v_R_BF	0.471	0.976	1.000	0.997	0.954	0.987	0.914	0.820
v_R_NL	0.468	0.972	0.997	1.000	0.951	0.984	0.912	0.819
v_R_NM	0.423	0.948	0.954	0.951	1.000	0.947	0.858	0.763
v_F_ML	0.463	0.965	0.987	0.984	0.947	1.000	0.906	0.813
v_F_KS	0.552	0.883	0.914	0.912	0.858	0.906	1.000	0.811
v_F_CS	0.566	0.806	0.820	0.819	0.763	0.813	0.811	1.000

5.4 Correlation Coefficients of Estimates—100 Trials

Table 7a: Correlation coefficients of estimated parameters across programs: The Boundary Separation *a*.

	a_R_EZ	a_R_BY	a_R_BF	a_R_NL	a_R_NM	a_F_ML	a_F_KS	a_F_CS
a_R_EZ	1.000	0.981	0.977	0.969	0.977	0.972	0.958	0.820
a_R_BY	0.981	1.000	0.998	0.992	0.997	0.993	0.952	0.808
a_R_BF	0.977	0.998	1.000	0.997	0.999	0.999	0.948	0.811
a_R_NL	0.969	0.992	0.997	1.000	0.996	0.996	0.941	0.811
a_R_NM	0.977	0.997	0.999	0.996	1.000	0.997	0.948	0.811
a_F_ML	0.972	0.993	0.999	0.996	0.997	1.000	0.944	0.811
a_F_KS	0.958	0.952	0.948	0.941	0.948	0.944	1.000	0.806
a_F_CS	0.820	0.808	0.811	0.811	0.811	0.811	0.806	1.000
z_R_EZ	(*)	(*)	(*)	(*)	(*)	(*)	(*)	(*)
z_R_BY	-0.014	-0.009	-0.011	-0.014	-0.012	-0.013	0.010	-0.048
z_R_BF	-0.009	-0.009	-0.009	-0.012	-0.010	-0.012	0.012	-0.047
z_R_NL	-0.010	-0.011	-0.011	-0.014	-0.012	-0.013	0.010	-0.047
z_R_NM	-0.010	-0.009	-0.009	-0.012	-0.011	-0.012	0.012	-0.047
z_F_ML	-0.010	-0.010	-0.010	-0.013	-0.011	-0.013	0.011	-0.048
z_F_KS	-0.001	0.001	0.001	-0.003	-0.000	-0.002	0.022	-0.046
z_F_CS	-0.014	-0.011	-0.011	-0.015	-0.012	-0.014	0.008	-0.068
t_R_EZ	-0.364	-0.299	-0.308	-0.306	-0.304	-0.305	-0.334	-0.290
t_R_BY	-0.015	-0.020	-0.022	-0.023	-0.020	-0.021	-0.014	-0.009
t_R_BF	0.016	-0.007	0.008	0.006	0.008	0.007	0.009	0.009
t_R_NL	0.013	-0.010	0.004	0.004	0.006	0.004	0.005	0.005
t_R_NM	0.015	-0.009	0.005	0.004	0.006	0.006	0.007	0.008
t_F_ML	0.018	-0.006	0.008	0.007	0.009	0.009	0.010	0.010
t_F_KS	0.003	-0.016	-0.004	-0.004	-0.002	-0.002	-0.011	0.005
t_F_CS	0.006	-0.018	-0.004	-0.004	-0.002	-0.003	-0.002	-0.002
v_R_EZ	-0.019	-0.023	-0.023	-0.025	-0.024	-0.024	-0.005	-0.054
v_R_BY	-0.036	-0.038	-0.042	-0.045	-0.044	-0.044	-0.019	-0.076
v_R_BF	-0.032	-0.041	-0.039	-0.042	-0.041	-0.041	-0.019	-0.071
v_R_NL	-0.028	-0.037	-0.035	-0.038	-0.037	-0.037	-0.015	-0.066
v_R_NM	-0.033	-0.044	-0.042	-0.045	-0.043	-0.044	-0.022	-0.070
v_F_ML	-0.031	-0.040	-0.039	-0.042	-0.040	-0.040	-0.018	-0.070
v_F_KS	-0.028	-0.040	-0.037	-0.039	-0.039	-0.038	-0.019	-0.078
v_F_CS	-0.022	-0.033	-0.031	-0.034	-0.033	-0.033	-0.015	-0.068

Table 7b: Correlation coefficients of estimated parameters across programs: The Starting Point / Bias z

	z_R_EZ	z_R_BY	z_R_BF	z_R_NL	z_R_NM	z_F_ML	z_F_KS	z_F_CS
a_R_EZ	(*)	-0.014	-0.009	-0.010	-0.010	-0.010	-0.001	-0.014
a_R_BY	(*)	-0.009	-0.009	-0.011	-0.009	-0.010	0.001	-0.011
a_R_BF	(*)	-0.011	-0.009	-0.011	-0.009	-0.010	0.001	-0.011
a_R_NL	(*)	-0.014	-0.012	-0.014	-0.012	-0.013	-0.003	-0.015
a_R_NM	(*)	-0.012	-0.010	-0.012	-0.011	-0.011	-0.000	-0.012
a_F_ML	(*)	-0.013	-0.012	-0.013	-0.012	-0.013	-0.002	-0.014
a_F_KS	(*)	0.010	0.012	0.010	0.012	0.011	0.022	0.008
a_F_CS	(*)	-0.048	-0.047	-0.047	-0.047	-0.048	-0.046	-0.068
z_R_EZ	(*)	(*)	(*)	(*)	(*)	(*)	(*)	(*)
z_R_BY	(*)	1.000	1.000	0.997	0.998	1.000	0.982	0.989
z_R_BF	(*)	1.000	1.000	0.997	0.998	1.000	0.982	0.989
z_R_NL	(*)	0.997	0.997	1.000	0.996	0.997	0.979	0.986
z_R_NM	(*)	0.998	0.998	0.996	1.000	0.998	0.980	0.988
z_F_ML	(*)	1.000	1.000	0.997	0.998	1.000	0.982	0.989
z_F_KS	(*)	0.982	0.982	0.979	0.980	0.982	1.000	0.978
z_F_CS	(*)	0.989	0.989	0.986	0.988	0.989	0.978	1.000
t_R_EZ	(*)	0.016	0.011	0.012	0.012	0.013	0.011	0.019
t_R_BY	(*)	-0.002	-0.004	-0.002	-0.003	-0.002	-0.002	-0.001
t_R_BF	(*)	-0.004	-0.001	-0.000	-0.002	-0.001	-0.001	0.001
t_R_NL	(*)	-0.002	-0.001	0.001	-0.000	0.001	0.001	0.003
t_R_NM	(*)	-0.003	-0.002	0.000	-0.001	0.000	-0.001	0.001
t_F_ML	(*)	-0.002	-0.001	0.001	-0.000	0.001	0.001	0.002
t_F_KS	(*)	-0.007	-0.005	-0.003	-0.005	-0.003	-0.006	-0.004
t_F_CS	(*)	-0.003	-0.001	0.001	-0.001	0.001	-0.001	-0.002
v_R_EZ	(*)	0.770	0.764	0.765	0.766	0.764	0.751	0.768
v_R_BY	(*)	-0.001	-0.010	-0.008	-0.005	-0.009	0.007	0.017
v_R_BF	(*)	-0.037	-0.045	-0.043	-0.040	-0.044	-0.029	-0.018
v_R_NL	(*)	-0.036	-0.044	-0.044	-0.039	-0.043	-0.028	-0.018
v_R_NM	(*)	-0.059	-0.066	-0.064	-0.071	-0.065	-0.048	-0.040
v_F_ML	(*)	-0.038	-0.046	-0.044	-0.040	-0.045	-0.029	-0.019
v_F_KS	(*)	0.038	0.030	0.032	0.036	0.031	0.013	0.048
v_F_CS	(*)	-0.043	-0.051	-0.048	-0.045	-0.049	-0.046	-0.034

Table 7c: Correlation coefficients of estimated parameters across programs: The encoding and reaction time T_{ER}.

	t_R_EZ	t_R_BY	t_R_BF	t_R_NL	t_R_NM	t_F_ML	t_F_KS	t_F_CS
a_R_EZ	-0.364	-0.015	0.016	0.013	0.015	0.018	0.003	0.006
a_R_BY	-0.299	-0.020	-0.007	-0.010	-0.009	-0.006	-0.016	-0.018
a_R_BF	-0.308	-0.022	0.008	0.004	0.005	0.008	-0.004	-0.004
a_R_NL	-0.306	-0.023	0.006	0.004	0.004	0.007	-0.004	-0.004
a_R_NM	-0.304	-0.020	0.008	0.006	0.006	0.009	-0.002	-0.002
a_F_ML	-0.305	-0.021	0.007	0.004	0.006	0.009	-0.002	-0.003
a_F_KS	-0.334	-0.014	0.009	0.005	0.007	0.010	-0.011	-0.002
a_F_CS	-0.290	-0.009	0.009	0.005	0.008	0.010	0.005	-0.002
z_R_EZ	(*)	(*)	(*)	(*)	(*)	(*)	(*)	(*)
z_R_BY	0.016	-0.002	-0.004	-0.002	-0.003	-0.002	-0.007	-0.003
z_R_BF	0.011	-0.004	-0.001	-0.001	-0.002	-0.001	-0.005	-0.001
z_R_NL	0.012	-0.002	-0.000	0.001	0.000	0.001	-0.003	0.001
z_R_NM	0.012	-0.003	-0.002	-0.000	-0.001	-0.000	-0.005	-0.001
z_F_ML	0.013	-0.002	-0.001	0.001	0.000	0.001	-0.003	0.001
z_F_KS	0.011	-0.002	-0.001	0.001	-0.001	0.001	-0.006	-0.001
z_F_CS	0.019	-0.001	0.001	0.003	0.001	0.002	-0.004	-0.002
t_R_EZ	1.000	0.825	0.809	0.811	0.810	0.810	0.812	0.808
t_R_BY	0.825	1.000	1.000	0.999	0.999	1.000	0.991	0.995
t_R_BF	0.809	1.000	1.000	0.999	0.999	1.000	0.991	0.995
t_R_NL	0.811	0.999	0.999	1.000	0.998	0.998	0.989	0.993
t_R_NM	0.810	0.999	0.999	0.998	1.000	0.999	0.990	0.994
t_F_ML	0.810	1.000	1.000	0.998	0.999	1.000	0.991	0.995
t_F_KS	0.812	0.991	0.991	0.989	0.990	0.991	1.000	0.991
t_F_CS	0.808	0.995	0.995	0.993	0.994	0.995	0.991	1.000
v_R_EZ	0.008	-0.000	-0.000	0.002	0.002	0.001	-0.002	0.001
v_R_BY	0.013	0.005	0.004	0.005	0.006	0.005	-0.000	0.003
v_R_BF	0.009	0.005	0.006	0.006	0.007	0.006	0.001	0.004
v_R_NL	0.006	0.002	0.002	0.001	0.004	0.003	-0.001	0.002
v_R_NM	0.013	0.010	0.011	0.012	0.012	0.012	0.008	0.010
v_F_ML	0.010	0.005	0.005	0.006	0.007	0.006	0.002	0.005
v_F_KS	0.006	0.006	0.007	0.008	0.009	0.008	0.005	0.007
v_F_CS	-0.005	-0.008	-0.007	-0.006	-0.005	-0.006	-0.009	-0.006

Table 7d: Correlation coefficients of estimated parameters across programs: The drift parameter ν.

	v_R_EZ	v_R_BY	v_R_BF	v_R_NL	v_R_NM	v_F_ML	v_F_KS	v_F_CS
a_R_EZ	-0.019	-0.036	-0.032	-0.028	-0.033	-0.031	-0.028	-0.022
a_R_BY	-0.023	-0.038	-0.041	-0.037	-0.044	-0.040	-0.040	-0.033
a_R_BF	-0.023	-0.042	-0.039	-0.035	-0.042	-0.039	-0.037	-0.031
a_R_NL	-0.025	-0.045	-0.042	-0.038	-0.045	-0.042	-0.039	-0.034
a_R_NM	-0.024	-0.044	-0.041	-0.037	-0.043	-0.040	-0.039	-0.033
a_F_ML	-0.024	-0.044	-0.041	-0.037	-0.044	-0.040	-0.038	-0.033
a_F_KS	-0.005	-0.019	-0.019	-0.015	-0.022	-0.018	-0.019	-0.015
a_F_CS	-0.054	-0.076	-0.071	-0.066	-0.070	-0.070	-0.078	-0.068
z_R_EZ	(*)	(*)	(*)	(*)	(*)	(*)	(*)	(*)
z_R_BY	0.770	-0.001	-0.037	-0.036	-0.059	-0.038	0.038	-0.043
z_R_BF	0.764	-0.010	-0.045	-0.044	-0.066	-0.046	0.030	-0.051
z_R_NL	0.765	-0.008	-0.043	-0.044	-0.064	-0.044	0.032	-0.048
z_R_NM	0.766	-0.005	-0.040	-0.039	-0.071	-0.040	0.036	-0.045
z_F_ML	0.764	-0.009	-0.044	-0.043	-0.065	-0.045	0.031	-0.049
z_F_KS	0.751	0.007	-0.029	-0.028	-0.048	-0.029	0.013	-0.046
z_F_CS	0.768	0.017	-0.018	-0.018	-0.040	-0.019	0.048	-0.034
t_R_EZ	0.008	0.013	0.009	0.006	0.013	0.010	0.006	-0.005
t_R_BY	-0.000	0.005	0.005	0.002	0.010	0.005	0.006	-0.008
t_R_BF	-0.000	0.004	0.006	0.002	0.011	0.005	0.007	-0.007
t_R_NL	0.002	0.005	0.006	0.001	0.012	0.006	0.008	-0.006
t_R_NM	0.002	0.006	0.007	0.004	0.012	0.007	0.009	-0.005
t_F_ML	0.001	0.005	0.006	0.003	0.012	0.006	0.008	-0.006
t_F_KS	-0.002	-0.000	0.001	-0.001	0.008	0.002	0.005	-0.009
t_F_CS	0.001	0.003	0.004	0.002	0.010	0.005	0.007	-0.006
v_R_EZ	1.000	0.478	0.446	0.445	0.395	0.445	0.484	0.388
v_R_BY	0.478	1.000	0.993	0.990	0.941	0.992	0.941	0.944
v_R_BF	0.446	0.993	1.000	0.998	0.941	1.000	0.947	0.949
v_R_NL	0.445	0.990	0.998	1.000	0.939	0.998	0.945	0.947
v_R_NM	0.395	0.941	0.941	0.939	1.000	0.941	0.887	0.894
v_F_ML	0.445	0.992	1.000	0.998	0.941	1.000	0.947	0.949
v_F_KS	0.484	0.941	0.947	0.945	0.887	0.947	1.000	0.938
v_F_CS	0.388	0.944	0.949	0.947	0.894	0.949	0.938	1.000

5.5 Correlation Coefficients of Estimates—400 Trials

Table 8a: Correlation coefficients of estimated parameters across programs: The Boundary Separation *a*.

	a_R_EZ	a_R_BY	a_R_BF	a_R_NL	a_R_NM	a_F_ML	a_F_KS	a_F_CS
a_R_EZ	1.000	0.985	0.985	0.980	0.985	0.985	0.981	0.953
a_R_BY	0.985	1.000	1.000	0.999	0.999	1.000	0.982	0.960
a_R_BF	0.985	1.000	1.000	0.999	1.000	1.000	0.982	0.962
a_R_NL	0.980	0.999	0.999	1.000	0.999	0.995	0.978	0.957
a_R_NM	0.985	0.999	1.000	0.999	1.000	1.000	0.982	0.962
a_F_ML	0.985	1.000	1.000	0.995	1.000	1.000	0.982	0.962
a_F_KS	0.981	0.982	0.982	0.978	0.982	0.982	1.000	0.958
a_F_CS	0.953	0.960	0.962	0.957	0.962	0.962	0.958	1.000
z_R_EZ	(*)	(*)	(*)	(*)	(*)	(*)	(*)	(*)
z_R_BY	0.000	0.000	0.000	-0.003	0.001	0.000	-0.011	-0.008
z_R_BF	-0.001	0.000	-0.001	-0.004	-0.000	-0.001	-0.013	-0.009
z_R_NL	-0.005	-0.003	-0.004	-0.004	-0.003	-0.005	-0.016	-0.012
z_R_NM	-0.000	0.001	-0.000	-0.003	0.001	0.000	-0.012	-0.008
z_F_ML	-0.000	0.000	-0.001	-0.004	0.000	-0.000	-0.012	-0.008
z_F_KS	-0.006	-0.004	-0.006	-0.009	-0.005	-0.005	-0.017	-0.014
z_F_CS	-0.000	-0.000	-0.001	-0.004	-0.000	-0.001	-0.013	-0.011
t_R_EZ	-0.347	-0.290	-0.300	-0.293	-0.297	-0.298	-0.310	-0.284
t_R_BY	-0.017	-0.017	-0.019	-0.018	-0.017	-0.017	-0.013	0.000
t_R_BF	-0.002	-0.015	-0.003	-0.004	-0.003	-0.003	0.001	0.012
t_R_NL	-0.006	-0.017	-0.007	-0.007	-0.005	-0.007	-0.002	0.009
t_R_NM	-0.000	-0.014	-0.003	-0.002	-0.001	-0.001	0.002	0.014
t_F_ML	-0.001	-0.014	-0.003	-0.001	-0.001	-0.002	0.002	0.013
t_F_KS	-0.003	-0.016	-0.004	-0.002	-0.002	-0.003	-0.003	0.012
t_F_CS	-0.004	-0.017	-0.006	-0.004	-0.004	-0.004	-0.002	0.010
v_R_EZ	-0.004	-0.005	-0.004	-0.005	-0.004	-0.003	-0.013	-0.010
v_R_BY	-0.015	-0.014	-0.014	-0.013	-0.014	-0.014	-0.021	-0.020
v_R_BF	-0.014	-0.015	-0.012	-0.011	-0.012	-0.012	-0.020	-0.017
v_R_NL	-0.007	-0.009	-0.006	-0.007	-0.005	-0.005	-0.013	-0.011
v_R_NM	-0.026	-0.027	-0.024	-0.023	-0.025	-0.024	-0.031	-0.029
v_F_ML	-0.013	-0.015	-0.012	-0.011	-0.012	-0.011	-0.019	-0.017
v_F_KS	-0.008	-0.011	-0.007	-0.007	-0.007	-0.007	-0.015	-0.014
v_F_CS	-0.013	-0.013	-0.011	-0.010	-0.011	-0.010	-0.019	-0.018

Table 8b: Correlation coefficients of estimated parameters across programs: The Starting Point / Bias z

	z_R_EZ	z_R_BY	z_R_BF	z_R_NL	z_R_NM	z_F_ML	z_F_KS	z_F_CS
a_R_EZ	(*)	0.000	-0.001	-0.005	-0.000	-0.000	-0.006	-0.000
a_R_BY	(*)	0.000	0.000	-0.003	0.001	0.000	-0.004	-0.000
a_R_BF	(*)	0.000	-0.001	-0.004	-0.000	-0.001	-0.006	-0.001
a_R_NL	(*)	-0.003	-0.004	-0.004	-0.003	-0.004	-0.009	-0.004
a_R_NM	(*)	0.001	-0.000	-0.003	0.001	0.000	-0.005	-0.000
a_F_ML	(*)	0.000	-0.001	-0.005	0.000	-0.000	-0.005	-0.001
a_F_KS	(*)	-0.011	-0.013	-0.016	-0.012	-0.012	-0.017	-0.013
a_F_CS	(*)	-0.008	-0.009	-0.012	-0.008	-0.008	-0.014	-0.011
z_R_EZ	(*)	(*)	(*)	(*)	(*)	(*)	(*)	(*)
z_R_BY	(*)	1.000	1.000	0.997	1.000	1.000	0.995	0.999
z_R_BF	(*)	1.000	1.000	0.997	1.000	1.000	0.995	0.999
z_R_NL	(*)	0.997	0.997	1.000	0.997	0.996	0.991	0.995
z_R_NM	(*)	1.000	1.000	0.997	1.000	1.000	0.995	0.998
z_F_ML	(*)	1.000	1.000	0.996	1.000	1.000	0.995	0.999
z_F_KS	(*)	0.995	0.995	0.991	0.995	0.995	1.000	0.995
z_F_CS	(*)	0.999	0.999	0.995	0.998	0.999	0.995	1.000
t_R_EZ	(*)	0.004	0.001	0.008	0.001	0.000	0.003	-0.003
t_R_BY	(*)	0.004	0.004	0.009	0.004	0.004	0.004	0.002
t_R_BF	(*)	0.004	-0.001	0.004	-0.001	-0.001	-0.001	-0.003
t_R_NL	(*)	0.006	0.000	0.005	0.001	0.001	0.001	-0.001
t_R_NM	(*)	0.004	-0.001	0.005	-0.000	-0.000	-0.001	-0.003
t_F_ML	(*)	0.004	-0.001	0.005	-0.000	-0.001	-0.001	-0.003
t_F_KS	(*)	0.005	0.000	0.006	0.001	0.000	-0.000	-0.002
t_F_CS	(*)	0.005	0.001	0.006	0.001	0.000	0.000	-0.002
v_R_EZ	(*)	0.790	0.782	0.785	0.785	0.783	0.781	0.783
v_R_BY	(*)	-0.004	-0.007	-0.001	-0.001	-0.006	0.002	-0.004
v_R_BF	(*)	-0.015	-0.022	-0.017	-0.017	-0.022	-0.015	-0.021
v_R_NL	(*)	-0.009	-0.017	-0.017	-0.011	-0.016	-0.008	-0.014
v_R_NM	(*)	-0.040	-0.047	-0.041	-0.048	-0.046	-0.039	-0.045
v_F_ML	(*)	-0.015	-0.023	-0.017	-0.017	-0.022	-0.014	-0.020
v_F_KS	(*)	0.006	-0.002	0.004	0.004	-0.001	-0.001	-0.002
v_F_CS	(*)	-0.036	-0.044	-0.038	-0.038	-0.043	-0.036	-0.044

Table 8c: Correlation coefficients of estimated parameters across programs: The encoding and reaction time T_{ER}.

	t_R_EZ	t_R_BY	t_R_BF	t_R_NL	t_R_NM	t_F_ML	t_F_KS	t_F_CS
a_R_EZ	-0.347	-0.017	-0.002	-0.006	-0.000	-0.001	-0.003	-0.004
a_R_BY	-0.290	-0.017	-0.015	-0.017	-0.014	-0.014	-0.016	-0.017
a_R_BF	-0.300	-0.019	-0.003	-0.007	-0.003	-0.003	-0.004	-0.006
a_R_NL	-0.293	-0.018	-0.004	-0.007	-0.002	-0.001	-0.002	-0.004
a_R_NM	-0.297	-0.017	-0.003	-0.005	-0.001	-0.001	-0.002	-0.004
a_F_ML	-0.298	-0.017	-0.003	-0.007	-0.001	-0.002	-0.003	-0.004
a_F_KS	-0.310	-0.013	0.001	-0.002	0.002	0.002	-0.003	-0.002
a_F_CS	-0.284	0.000	0.012	0.009	0.014	0.013	0.012	0.010
z_R_EZ	(*)	(*)	(*)	(*)	(*)	(*)	(*)	(*)
z_R_BY	0.004	0.004	0.004	0.006	0.004	0.004	0.005	0.005
z_R_BF	0.001	0.004	-0.001	0.000	-0.001	-0.001	0.000	0.001
z_R_NL	0.008	0.009	0.004	0.005	0.005	0.005	0.006	0.006
z_R_NM	0.001	0.004	-0.001	0.001	-0.000	-0.000	0.001	0.001
z_F_ML	0.000	0.004	-0.001	0.001	-0.000	-0.001	0.000	0.000
z_F_KS	0.003	0.004	-0.001	0.001	-0.001	-0.001	-0.000	0.000
z_F_CS	-0.003	0.002	-0.003	-0.001	-0.003	-0.003	-0.002	-0.002
t_R_EZ	1.000	0.856	0.838	0.839	0.839	0.839	0.838	0.837
t_R_BY	0.856	1.000	1.000	0.999	1.000	1.000	0.998	0.999
t_R_BF	0.838	1.000	1.000	0.999	1.000	1.000	0.997	0.999
t_R_NL	0.839	0.999	0.999	1.000	0.999	0.998	0.995	0.997
t_R_NM	0.839	1.000	1.000	0.999	1.000	1.000	0.997	0.999
t_F_ML	0.839	1.000	1.000	0.998	1.000	1.000	0.997	0.999
t_F_KS	0.838	0.998	0.997	0.995	0.997	0.997	1.000	0.998
t_F_CS	0.837	0.999	0.999	0.997	0.999	0.999	0.998	1.000
v_R_EZ	0.000	-0.001	-0.001	-0.001	-0.001	-0.001	0.001	-0.000
v_R_BY	0.005	0.002	0.003	0.002	0.002	0.002	0.004	0.004
v_R_BF	0.008	0.003	0.005	0.005	0.005	0.005	0.008	0.006
v_R_NL	-0.000	-0.004	-0.001	-0.004	-0.002	-0.002	0.000	-0.001
v_R_NM	0.014	0.007	0.010	0.009	0.009	0.009	0.011	0.010
v_F_ML	0.007	0.002	0.005	0.004	0.004	0.004	0.007	0.005
v_F_KS	-0.002	-0.005	-0.002	-0.004	-0.003	-0.003	-0.001	-0.002
v_F_CS	0.010	0.004	0.007	0.006	0.006	0.006	0.008	0.007

Table 8d: Correlation coefficients of estimated parameters across programs: The drift parameter ν.

	v_R_EZ	v_R_BY	v_R_BF	v_R_NL	v_R_NM	v_F_ML	v_F_KS	v_F_CS
a_R_EZ	-0.004	-0.015	-0.014	-0.007	-0.026	-0.013	-0.008	-0.013
a_R_BY	-0.005	-0.014	-0.015	-0.009	-0.027	-0.015	-0.011	-0.013
a_R_BF	-0.004	-0.014	-0.012	-0.006	-0.024	-0.012	-0.007	-0.011
a_R_NL	-0.005	-0.013	-0.011	-0.007	-0.023	-0.011	-0.007	-0.010
a_R_NM	-0.004	-0.014	-0.012	-0.005	-0.025	-0.012	-0.007	-0.011
a_F_ML	-0.003	-0.014	-0.012	-0.005	-0.024	-0.011	-0.007	-0.010
a_F_KS	-0.013	-0.021	-0.020	-0.013	-0.031	-0.019	-0.015	-0.019
a_F_CS	-0.010	-0.020	-0.017	-0.011	-0.029	-0.017	-0.014	-0.018
z_R_EZ	(*)	(*)	(*)	(*)	(*)	(*)	(*)	(*)
z_R_BY	0.790	-0.004	-0.015	-0.009	-0.040	-0.015	0.006	-0.036
z_R_BF	0.782	-0.007	-0.022	-0.017	-0.047	-0.023	-0.002	-0.044
z_R_NL	0.785	-0.001	-0.017	-0.017	-0.041	-0.017	0.004	-0.038
z_R_NM	0.785	-0.001	-0.017	-0.011	-0.048	-0.017	0.004	-0.038
z_F_ML	0.783	-0.006	-0.022	-0.016	-0.046	-0.022	-0.001	-0.043
z_F_KS	0.781	0.002	-0.015	-0.008	-0.039	-0.014	-0.001	-0.036
z_F_CS	0.783	-0.004	-0.021	-0.014	-0.045	-0.020	-0.002	-0.044
t_R_EZ	0.000	0.005	0.008	-0.000	0.014	0.007	-0.002	0.010
t_R_BY	-0.001	0.002	0.003	-0.004	0.007	0.002	-0.005	0.004
t_R_BF	-0.001	0.003	0.005	-0.001	0.010	0.005	-0.002	0.007
t_R_NL	-0.001	0.002	0.005	-0.004	0.009	0.004	-0.004	0.006
t_R_NM	-0.001	0.002	0.005	-0.002	0.009	0.004	-0.003	0.006
t_F_ML	-0.001	0.002	0.005	-0.002	0.009	0.004	-0.003	0.006
t_F_KS	0.001	0.004	0.008	0.000	0.011	0.007	-0.001	0.008
t_F_CS	-0.000	0.004	0.006	-0.001	0.010	0.005	-0.002	0.007
v_R_EZ	1.000	0.448	0.438	0.438	0.387	0.437	0.445	0.412
v_R_BY	0.448	1.000	0.999	0.997	0.955	0.999	0.988	0.994
v_R_BF	0.438	0.999	1.000	0.997	0.954	1.000	0.988	0.995
v_R_NL	0.438	0.997	0.997	1.000	0.952	0.997	0.985	0.992
v_R_NM	0.387	0.955	0.954	0.952	1.000	0.955	0.945	0.951
v_F_ML	0.437	0.999	1.000	0.997	0.955	1.000	0.988	0.995
v_F_KS	0.445	0.988	0.988	0.985	0.945	0.988	1.000	0.989
v_F_CS	0.412	0.994	0.995	0.992	0.951	0.995	0.989	1.000

5.6 Plots of Correlation Coefficients of Estimates

Figures 3a, 3b, 3c, and 3d show all pairwise scatter plots of the 8 estimation methods. The colors indicate the number of trials (black: 50; red: 100; green: 400)

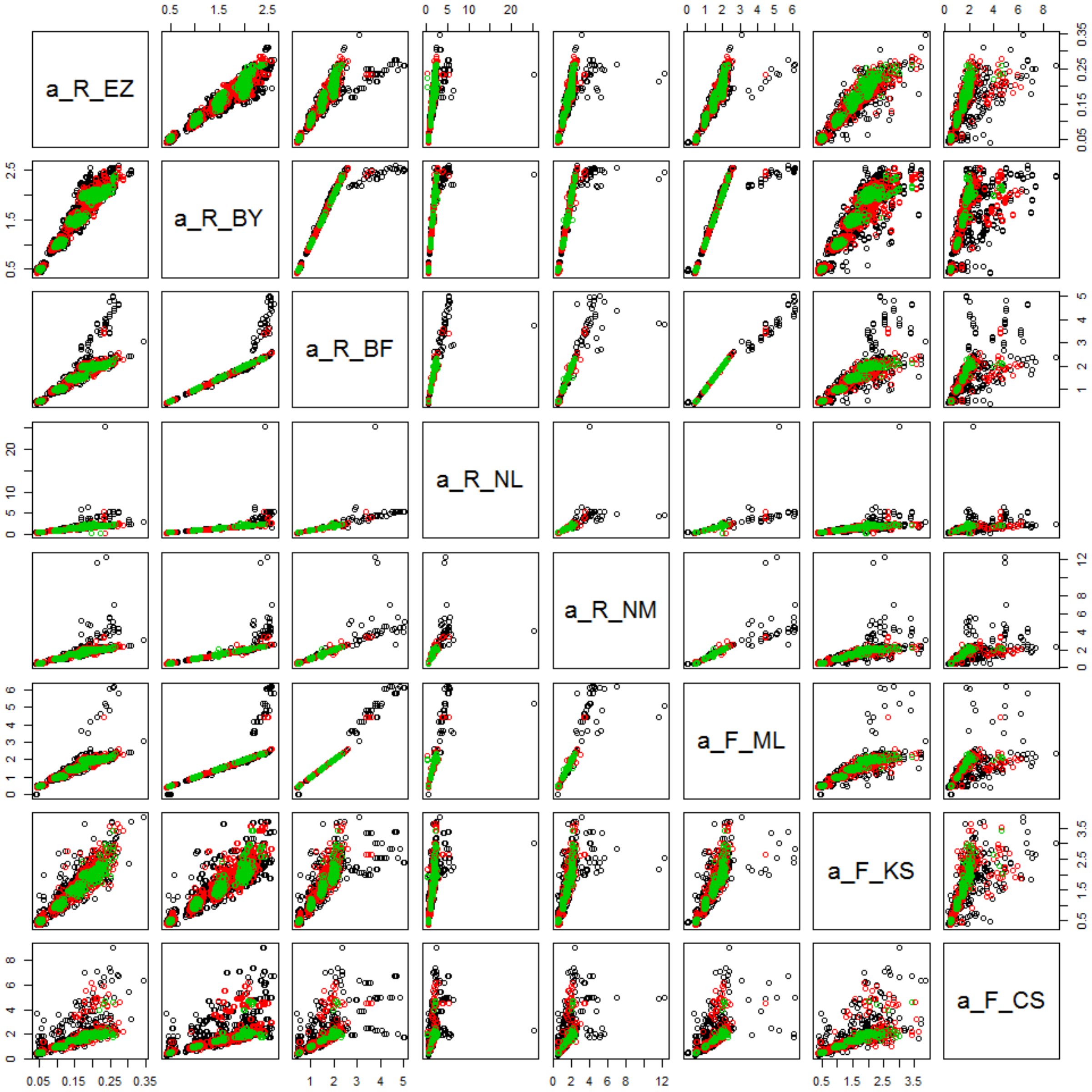

Figure 3a: Scatter plots of the estimates of boundary separation a.

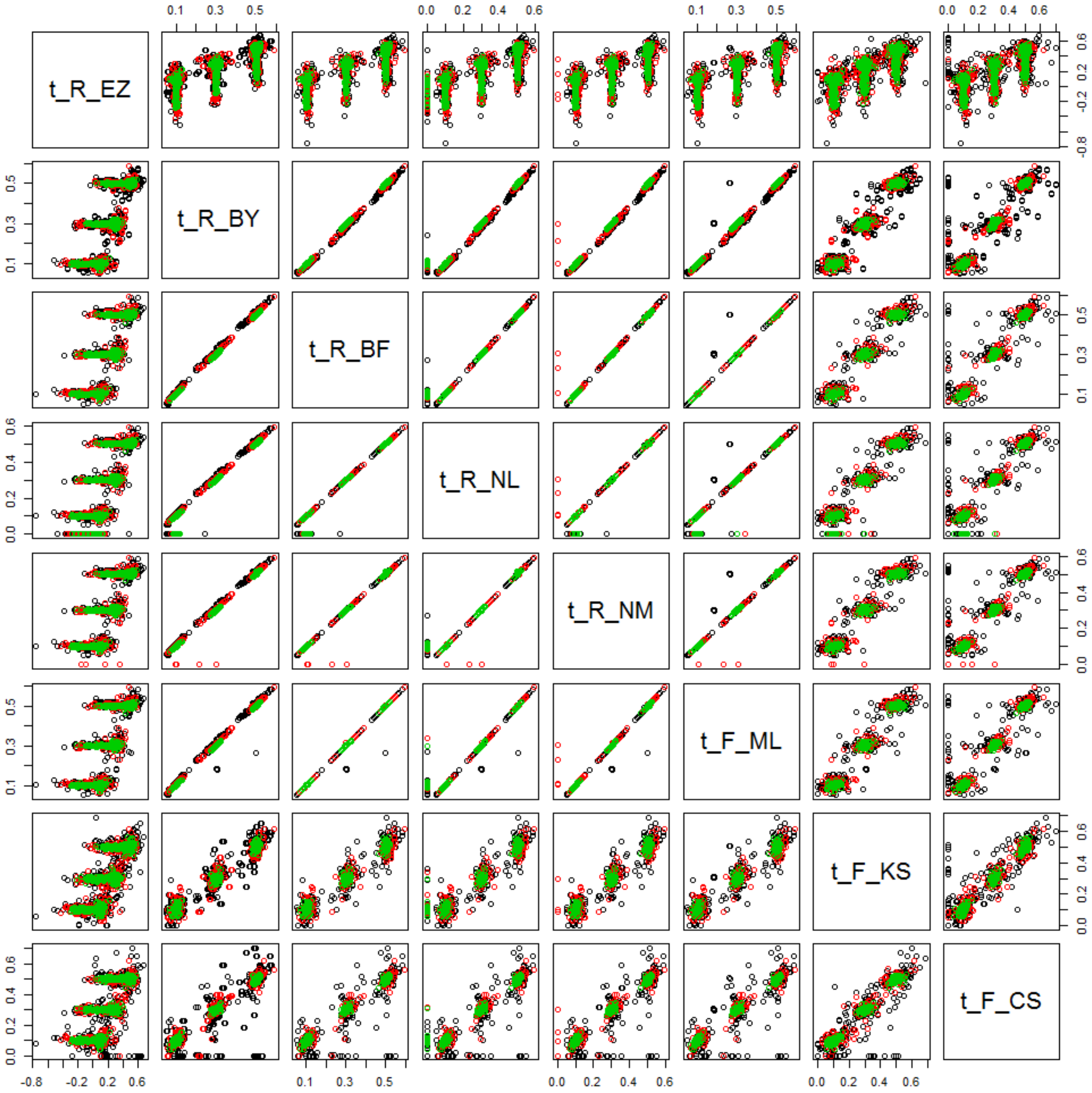

Figure 3b: Scatter plots of the estimates of the bias parameter z.

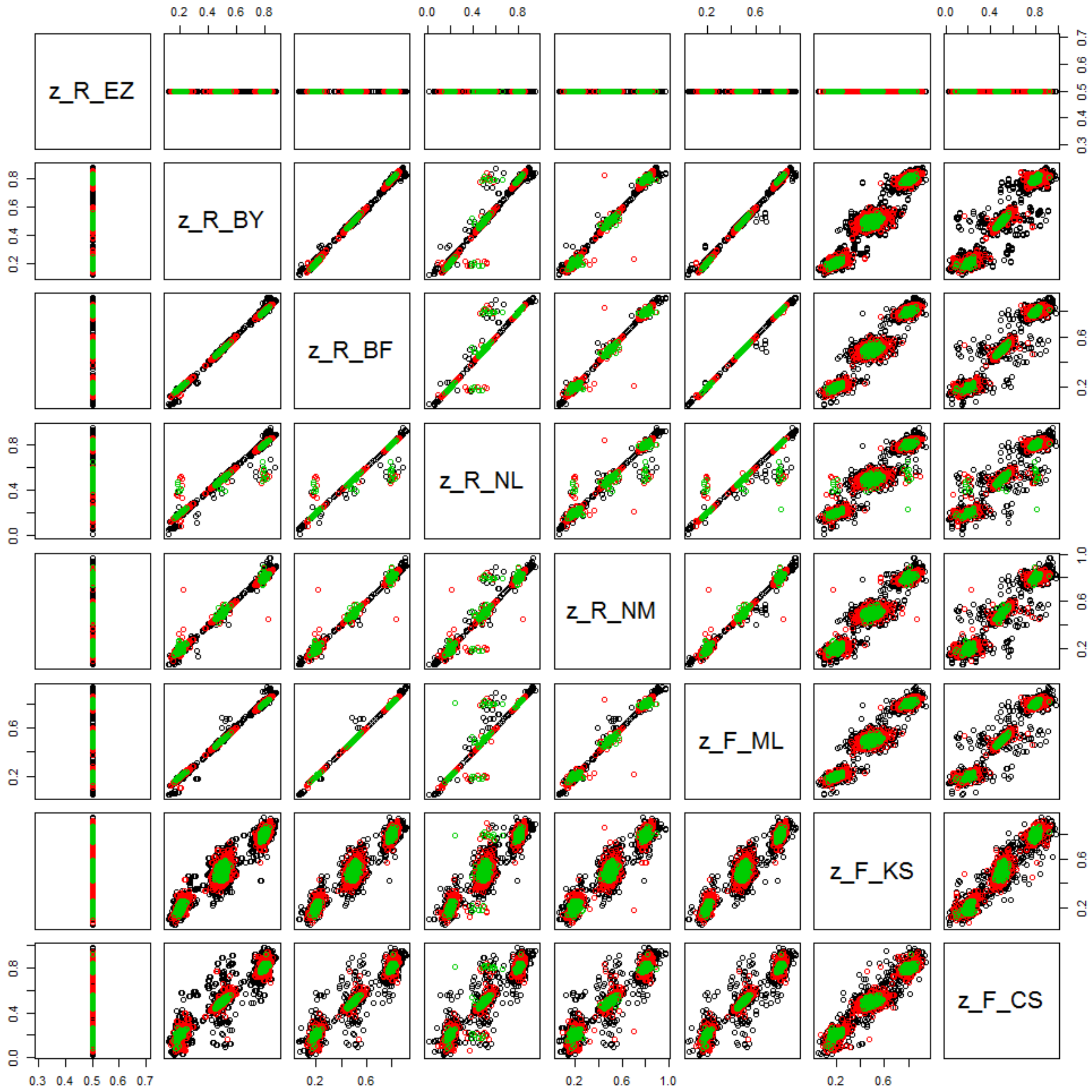

Figure 3c: Scatter plots of the estimates of the encoding and response time parameter T_{ER}. Note: The degenerate structure in the first row and the first column is a result of the EZ method not estimating z, but rather setting it to 0.5.

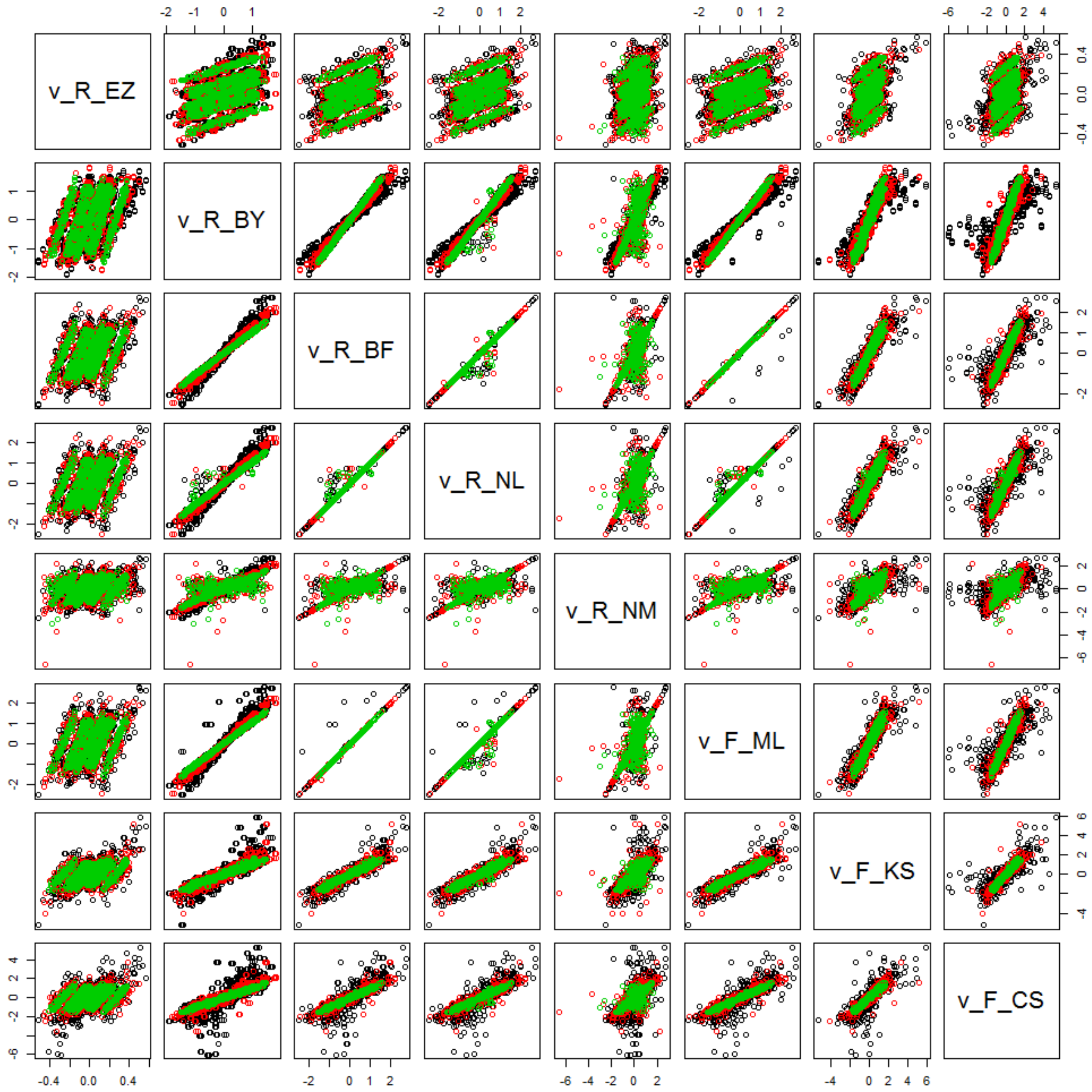

Figure 3d: Scatter plots of the estimates of the drift parameter ν.

6 Descriptive Statistics of Parameter Estimates and Parameter Recovery Performance Measures

6.1 Boundary Separation a

Table 9: Descriptive statistics and parameter recovery performance measures for the boundary separation parameter a. Notes: method = estimation method; trials = number of trials; n = effective number of available values (only here); mean = mean of parameter estimates; sd = standard deviation of parameter estimates; bias = mean parameter minus true parameter; relbias = bias divided by true parameter; mse = mean squared error; rmse = root mean squared error.

method	trials	true	n	mean	sd	bias	relbias	mse	rmse
a_R_EZ	50.00	0.50	675.00	0.51	0.06	0.01	1.87	0.00	0.06
a_R_EZ	100.00	0.50	675.00	0.51	0.04	0.01	2.59	0.00	0.05
a_R_EZ	400.00	0.50	675.00	0.52	0.02	0.02	3.57	0.00	0.03
a_R_EZ	50.00	1.00	675.00	1.02	0.13	0.02	2.08	0.02	0.13
a_R_EZ	100.00	1.00	675.00	1.02	0.09	0.02	1.74	0.01	0.10
a_R_EZ	400.00	1.00	675.00	1.03	0.06	0.03	3.02	0.00	0.07
a_R_EZ	50.00	1.50	675.00	1.54	0.20	0.04	2.68	0.04	0.20
a_R_EZ	100.00	1.50	675.00	1.54	0.16	0.04	2.76	0.03	0.16
a_R_EZ	400.00	1.50	675.00	1.54	0.11	0.04	2.50	0.01	0.12
a_R_EZ	50.00	2.00	675.00	2.04	0.29	0.04	1.82	0.09	0.29
a_R_EZ	100.00	2.00	675.00	2.05	0.25	0.05	2.39	0.06	0.25
a_R_EZ	400.00	2.00	675.00	2.05	0.18	0.05	2.55	0.04	0.19
a_R_BY	50.00	0.50	675.00	0.51	0.04	0.01	1.19	0.00	0.04
a_R_BY	100.00	0.50	675.00	0.50	0.03	0.00	0.56	0.00	0.03
a_R_BY	400.00	0.50	675.00	0.50	0.01	0.00	0.25	0.00	0.01
a_R_BY	50.00	1.00	675.00	1.02	0.09	0.02	1.80	0.01	0.09
a_R_BY	100.00	1.00	675.00	1.01	0.06	0.01	0.74	0.00	0.06
a_R_BY	400.00	1.00	675.00	1.00	0.03	0.00	0.16	0.00	0.03
a_R_BY	50.00	1.50	675.00	1.54	0.16	0.04	2.59	0.03	0.16
a_R_BY	100.00	1.50	675.00	1.52	0.10	0.02	1.59	0.01	0.10
a_R_BY	400.00	1.50	675.00	1.50	0.04	0.00	0.01	0.00	0.04
a_R_BY	50.00	2.00	660.00	2.06	0.20	0.06	2.76	0.04	0.20
a_R_BY	100.00	2.00	639.00	2.02	0.14	0.02	1.15	0.02	0.14
a_R_BY	400.00	2.00	564.00	2.01	0.07	0.01	0.73	0.01	0.07
a_R_BF	50.00	0.50	675.00	0.50	0.04	-0.00	-0.07	0.00	0.04
a_R_BF	100.00	0.50	675.00	0.50	0.03	0.00	0.07	0.00	0.03
a_R_BF	400.00	0.50	675.00	0.50	0.01	0.00	0.18	0.00	0.01
a_R_BF	50.00	1.00	675.00	1.00	0.09	0.00	0.29	0.01	0.09
a_R_BF	100.00	1.00	675.00	1.00	0.06	0.00	0.05	0.00	0.06
a_R_BF	400.00	1.00	675.00	1.00	0.03	-0.00	-0.01	0.00	0.03
a_R_BF	50.00	1.50	675.00	1.53	0.25	0.03	1.75	0.07	0.26
a_R_BF	100.00	1.50	675.00	1.51	0.10	0.01	0.82	0.01	0.10
a_R_BF	400.00	1.50	675.00	1.50	0.05	-0.00	-0.14	0.00	0.05
a_R_BF	50.00	2.00	675.00	2.13	0.74	0.13	6.40	0.56	0.75
a_R_BF	100.00	2.00	674.00	2.01	0.17	0.01	0.56	0.03	0.17
a_R_BF	400.00	2.00	673.00	2.01	0.07	0.01	0.52	0.00	0.07
a_R_NL	50.00	0.50	673.00	0.50	0.04	-0.00	-0.14	0.00	0.04
a_R_NL	100.00	0.50	675.00	0.50	0.03	-0.00	-0.08	0.00	0.03
a_R_NL	400.00	0.50	673.00	0.50	0.01	0.00	0.08	0.00	0.01
a_R_NL	50.00	1.00	674.00	1.00	0.09	0.00	0.20	0.01	0.09
a_R_NL	100.00	1.00	673.00	1.00	0.06	0.00	0.02	0.00	0.06
a_R_NL	400.00	1.00	671.00	1.00	0.03	-0.00	-0.00	0.00	0.03
a_R_NL	50.00	1.50	671.00	1.52	0.20	0.02	1.35	0.04	0.20
a_R_NL	100.00	1.50	671.00	1.51	0.10	0.01	0.72	0.01	0.10
a_R_NL	400.00	1.50	673.00	1.50	0.04	-0.00	-0.19	0.00	0.04
a_R_NL	50.00	2.00	671.00	2.10	0.47	0.10	4.85	0.23	0.48
a_R_NL	100.00	2.00	667.00	2.01	0.17	0.01	0.58	0.03	0.17
a_R_NL	400.00	2.00	670.00	2.01	0.07	0.01	0.53	0.00	0.07
a_R_NM	50.00	0.50	675.00	0.50	0.04	-0.00	-0.11	0.00	0.04
a_R_NM	100.00	0.50	675.00	0.50	0.03	-0.00	-0.07	0.00	0.03
a_R_NM	400.00	0.50	675.00	0.50	0.01	0.00	0.08	0.00	0.01

a_R_NM	50.00	1.00	675.00	1.00	0.09	0.00	0.19	0.01	0.09
a_R_NM	100.00	1.00	675.00	1.00	0.06	0.00	0.02	0.00	0.06
a_R_NM	400.00	1.00	675.00	1.00	0.03	-0.00	-0.01	0.00	0.03
a_R_NM	50.00	1.50	675.00	1.53	0.28	0.03	1.94	0.08	0.29
a_R_NM	100.00	1.50	675.00	1.51	0.10	0.01	0.71	0.01	0.10
a_R_NM	400.00	1.50	674.00	1.50	0.04	-0.00	-0.14	0.00	0.04
a_R_NM	50.00	2.00	675.00	2.16	1.07	0.16	7.97	1.17	1.08
a_R_NM	100.00	2.00	675.00	2.02	0.23	0.01	0.75	0.05	0.23
a_R_NM	400.00	2.00	672.00	2.01	0.13	0.01	0.48	0.02	0.13
a_F_ML	50.00	0.50	675.00	0.52	0.16	0.02	4.85	0.02	0.16
a_F_ML	100.00	0.50	675.00	0.50	0.06	0.00	0.59	0.00	0.06
a_F_ML	400.00	0.50	675.00	0.50	0.02	0.00	0.09	0.00	0.02
a_F_ML	50.00	1.00	675.00	1.07	0.29	0.07	7.47	0.09	0.30
a_F_ML	100.00	1.00	675.00	1.02	0.19	0.02	2.31	0.04	0.19
a_F_ML	400.00	1.00	675.00	1.01	0.06	0.01	0.87	0.00	0.06
a_F_ML	50.00	1.50	675.00	1.62	0.33	0.12	8.08	0.12	0.35
a_F_ML	100.00	1.50	675.00	1.59	0.25	0.09	6.29	0.07	0.27
a_F_ML	400.00	1.50	675.00	1.52	0.11	0.02	1.21	0.01	0.11
a_F_ML	50.00	2.00	675.00	2.16	0.41	0.16	8.01	0.19	0.44
a_F_ML	100.00	2.00	675.00	2.14	0.36	0.14	6.99	0.15	0.39
a_F_ML	400.00	2.00	675.00	2.07	0.21	0.07	3.35	0.05	0.22
a_F_KS	50.00	0.50	675.00	0.49	0.09	-0.01	-2.46	0.01	0.09
a_F_KS	100.00	0.50	675.00	0.50	0.03	-0.00	-0.08	0.00	0.03
a_F_KS	400.00	0.50	675.00	0.50	0.01	0.00	0.08	0.00	0.01
a_F_KS	50.00	1.00	675.00	1.00	0.09	0.00	0.19	0.01	0.09
a_F_KS	100.00	1.00	675.00	1.00	0.06	0.00	0.02	0.00	0.06
a_F_KS	400.00	1.00	675.00	1.00	0.03	-0.00	-0.01	0.00	0.03
a_F_KS	50.00	1.50	675.00	1.53	0.26	0.03	1.84	0.07	0.26
a_F_KS	100.00	1.50	675.00	1.51	0.10	0.01	0.71	0.01	0.10
a_F_KS	400.00	1.50	675.00	1.50	0.04	-0.00	-0.19	0.00	0.04
a_F_KS	50.00	2.00	675.00	2.15	0.70	0.15	7.52	0.52	0.72
a_F_KS	100.00	2.00	675.00	2.02	0.21	0.02	0.79	0.05	0.21
a_F_KS	400.00	2.00	675.00	2.01	0.07	0.01	0.53	0.00	0.07
a_F_CS	50.00	0.50	675.00	0.60	0.38	0.10	19.13	0.15	0.39
a_F_CS	100.00	0.50	675.00	0.52	0.10	0.02	3.33	0.01	0.10
a_F_CS	400.00	0.50	675.00	0.50	0.02	0.00	0.19	0.00	0.02
a_F_CS	50.00	1.00	675.00	1.15	0.50	0.15	14.75	0.28	0.52
a_F_CS	100.00	1.00	675.00	1.01	0.12	0.01	1.23	0.01	0.12
a_F_CS	400.00	1.00	675.00	1.00	0.03	0.00	0.25	0.00	0.03
a_F_CS	50.00	1.50	675.00	1.76	0.74	0.26	17.49	0.61	0.78
a_F_CS	100.00	1.50	675.00	1.66	0.59	0.16	10.36	0.37	0.61
a_F_CS	400.00	1.50	675.00	1.50	0.06	-0.00	-0.07	0.00	0.06
a_F_CS	50.00	2.00	675.00	2.50	1.22	0.50	25.22	1.74	1.32
a_F_CS	100.00	2.00	675.00	2.23	0.78	0.23	11.65	0.66	0.81
a_F_CS	400.00	2.00	675.00	2.05	0.34	0.05	2.42	0.11	0.34

6.2 Starting Point / Bias z

Table 10: Descriptive statistics and parameter recovery performance measures for the starting point z. Notes as in Table 9.

method	trials	true	n	mean	sd	bias	relbias	mse	rmse
z_R_EZ	50.00	0.20	900.00	0.50	0.00	0.30	150.00	0.09	0.30
z_R_EZ	100.00	0.20	900.00	0.50	0.00	0.30	150.00	0.09	0.30
z_R_EZ	400.00	0.20	900.00	0.50	0.00	0.30	150.00	0.09	0.30
z_R_EZ	50.00	0.50	900.00	0.50	0.00	0.00	0.00	0.00	0.00
z_R_EZ	100.00	0.50	900.00	0.50	0.00	0.00	0.00	0.00	0.00
z_R_EZ	400.00	0.50	900.00	0.50	0.00	0.00	0.00	0.00	0.00
z_R_EZ	50.00	0.80	900.00	0.50	0.00	-0.30	-37.50	0.09	0.30
z_R_EZ	100.00	0.80	900.00	0.50	0.00	-0.30	-37.50	0.09	0.30
z_R_EZ	400.00	0.80	900.00	0.50	0.00	-0.30	-37.50	0.09	0.30
z_R_BY	50.00	0.20	897.00	0.21	0.03	0.01	4.28	0.00	0.04
z_R_BY	100.00	0.20	891.00	0.20	0.02	0.00	1.67	0.00	0.03
z_R_BY	400.00	0.20	864.00	0.20	0.01	0.00	0.08	0.00	0.01
z_R_BY	50.00	0.50	894.00	0.50	0.05	0.00	0.87	0.00	0.05
z_R_BY	100.00	0.50	885.00	0.50	0.04	0.00	0.08	0.00	0.04
z_R_BY	400.00	0.50	858.00	0.50	0.02	-0.00	-0.04	0.00	0.02
z_R_BY	50.00	0.80	894.00	0.79	0.04	-0.01	-1.30	0.00	0.04
z_R_BY	100.00	0.80	888.00	0.80	0.02	-0.00	-0.27	0.00	0.02
z_R_BY	400.00	0.80	867.00	0.80	0.01	-0.00	-0.28	0.00	0.01
z_R_BF	50.00	0.20	900.00	0.19	0.04	-0.01	-3.79	0.00	0.04
z_R_BF	100.00	0.20	900.00	0.20	0.03	-0.00	-1.95	0.00	0.03
z_R_BF	400.00	0.20	899.00	0.20	0.01	-0.00	-1.10	0.00	0.01
z_R_BF	50.00	0.50	900.00	0.50	0.06	0.00	0.78	0.00	0.06
z_R_BF	100.00	0.50	899.00	0.50	0.04	-0.00	-0.05	0.00	0.04
z_R_BF	400.00	0.50	900.00	0.50	0.02	0.00	0.02	0.00	0.02
z_R_BF	50.00	0.80	900.00	0.81	0.04	0.01	0.64	0.00	0.04
z_R_BF	100.00	0.80	900.00	0.80	0.03	0.00	0.58	0.00	0.03
z_R_BF	400.00	0.80	899.00	0.80	0.01	0.00	0.00	0.00	0.01
z_R_NL	50.00	0.20	895.00	0.19	0.04	-0.01	-3.26	0.00	0.04
z_R_NL	100.00	0.20	895.00	0.20	0.03	-0.00	-1.87	0.00	0.03
z_R_NL	400.00	0.20	895.00	0.20	0.01	-0.00	-0.84	0.00	0.01
z_R_NL	50.00	0.50	898.00	0.50	0.06	0.00	0.87	0.00	0.06
z_R_NL	100.00	0.50	892.00	0.50	0.04	-0.00	-0.02	0.00	0.04
z_R_NL	400.00	0.50	896.00	0.50	0.02	0.00	0.02	0.00	0.02
z_R_NL	50.00	0.80	896.00	0.80	0.04	0.00	0.59	0.00	0.04
z_R_NL	100.00	0.80	899.00	0.80	0.02	0.00	0.60	0.00	0.02
z_R_NL	400.00	0.80	896.00	0.80	0.01	-0.00	-0.03	0.00	0.01
z_R_NM	50.00	0.20	900.00	0.19	0.04	-0.01	-3.26	0.00	0.04
z_R_NM	100.00	0.20	900.00	0.20	0.03	-0.00	-1.00	0.00	0.03
z_R_NM	400.00	0.20	900.00	0.20	0.02	-0.00	-0.01	0.00	0.02
z_R_NM	50.00	0.50	900.00	0.50	0.06	0.00	0.77	0.00	0.06
z_R_NM	100.00	0.50	900.00	0.50	0.04	-0.00	-0.05	0.00	0.04
z_R_NM	400.00	0.50	900.00	0.50	0.02	-0.00	-0.06	0.00	0.02
z_R_NM	50.00	0.80	900.00	0.80	0.05	0.00	0.31	0.00	0.05
z_R_NM	100.00	0.80	900.00	0.80	0.03	0.00	0.37	0.00	0.03
z_R_NM	400.00	0.80	896.00	0.80	0.03	-0.00	-0.42	0.00	0.03
z_F_ML	50.00	0.20	900.00	0.20	0.06	0.00	1.04	0.00	0.06
z_F_ML	100.00	0.20	900.00	0.20	0.05	-0.00	-0.50	0.00	0.05
z_F_ML	400.00	0.20	900.00	0.20	0.02	-0.00	-1.60	0.00	0.02
z_F_ML	50.00	0.50	900.00	0.50	0.11	-0.00	-0.72	0.01	0.11
z_F_ML	100.00	0.50	900.00	0.50	0.07	-0.00	-0.82	0.01	0.07
z_F_ML	400.00	0.50	900.00	0.50	0.04	0.00	0.03	0.00	0.04
z_F_ML	50.00	0.80	900.00	0.80	0.07	0.00	0.01	0.00	0.07
z_F_ML	100.00	0.80	900.00	0.80	0.04	0.00	0.49	0.00	0.04
z_F_ML	400.00	0.80	900.00	0.80	0.02	-0.00	-0.05	0.00	0.02
z_F_KS	50.00	0.20	900.00	0.19	0.04	-0.01	-3.57	0.00	0.04
z_F_KS	100.00	0.20	900.00	0.20	0.03	-0.00	-1.94	0.00	0.03
z_F_KS	400.00	0.20	900.00	0.20	0.01	-0.00	-0.84	0.00	0.01
z_F_KS	50.00	0.50	900.00	0.51	0.06	0.01	1.36	0.00	0.06
z_F_KS	100.00	0.50	900.00	0.50	0.04	-0.00	-0.05	0.00	0.04

z_F_KS	400.00	0.50	900.00	0.50	0.02	0.00	0.01	0.00	0.02
z_F_KS	50.00	0.80	900.00	0.81	0.04	0.01	0.67	0.00	0.04
z_F_KS	100.00	0.80	900.00	0.81	0.02	0.01	0.63	0.00	0.03
z_F_KS	400.00	0.80	900.00	0.80	0.01	-0.00	-0.03	0.00	0.01
z_F_CS	50.00	0.20	900.00	0.20	0.09	0.00	1.03	0.01	0.09
z_F_CS	100.00	0.20	900.00	0.19	0.05	-0.01	-2.77	0.00	0.05
z_F_CS	400.00	0.20	900.00	0.20	0.02	-0.00	-0.29	0.00	0.02
z_F_CS	50.00	0.50	900.00	0.50	0.12	0.00	0.85	0.01	0.12
z_F_CS	100.00	0.50	900.00	0.50	0.05	0.00	0.06	0.00	0.05
z_F_CS	400.00	0.50	900.00	0.50	0.02	-0.00	-0.03	0.00	0.02
z_F_CS	50.00	0.80	900.00	0.80	0.08	0.00	0.46	0.01	0.08
z_F_CS	100.00	0.80	900.00	0.80	0.04	0.00	0.52	0.00	0.04
z_F_CS	400.00	0.80	900.00	0.80	0.02	-0.00	-0.09	0.00	0.02

6.3 Encoding and Reaction Time T_{ER}

Table 11: Descriptive statistics and parameter recovery performance measures for the encoding and reaction time T_{ER}. Notes as in Table 9.

method	trials	true	n	mean	sd	bias	relbias	mse	rmse
t_R_EZ	50.00	0.10	900.00	0.03	0.13	-0.07	-72.34	0.02	0.14
t_R_EZ	100.00	0.10	900.00	0.02	0.12	-0.08	-75.43	0.02	0.14
t_R_EZ	400.00	0.10	900.00	0.02	0.10	-0.08	-81.51	0.02	0.13
t_R_EZ	50.00	0.30	900.00	0.22	0.13	-0.08	-25.16	0.02	0.15
t_R_EZ	100.00	0.30	900.00	0.22	0.12	-0.08	-27.41	0.02	0.15
t_R_EZ	400.00	0.30	900.00	0.22	0.11	-0.08	-27.53	0.02	0.14
t_R_EZ	50.00	0.50	900.00	0.42	0.12	-0.08	-15.90	0.02	0.15
t_R_EZ	100.00	0.50	900.00	0.42	0.11	-0.08	-16.14	0.02	0.14
t_R_EZ	400.00	0.50	900.00	0.42	0.11	-0.08	-16.36	0.02	0.13
t_R_BY	50.00	0.10	894.00	0.10	0.01	-0.00	-0.31	0.00	0.01
t_R_BY	100.00	0.10	900.00	0.10	0.01	0.00	0.50	0.00	0.01
t_R_BY	400.00	0.10	876.00	0.10	0.01	-0.00	-0.58	0.00	0.01
t_R_BY	50.00	0.30	897.00	0.30	0.02	-0.00	-0.68	0.00	0.02
t_R_BY	100.00	0.30	888.00	0.30	0.01	-0.00	-0.51	0.00	0.01
t_R_BY	400.00	0.30	855.00	0.30	0.01	-0.00	-0.07	0.00	0.01
t_R_BY	50.00	0.50	894.00	0.50	0.02	-0.00	-0.26	0.00	0.02
t_R_BY	100.00	0.50	876.00	0.50	0.01	0.00	0.04	0.00	0.01
t_R_BY	400.00	0.50	858.00	0.50	0.00	0.00	0.04	0.00	0.00
t_R_BF	50.00	0.10	900.00	0.11	0.02	0.01	5.15	0.00	0.02
t_R_BF	100.00	0.10	900.00	0.10	0.01	0.00	3.19	0.00	0.01
t_R_BF	400.00	0.10	899.00	0.10	0.01	0.00	0.10	0.00	0.01
t_R_BF	50.00	0.30	900.00	0.30	0.02	0.00	1.49	0.00	0.02
t_R_BF	100.00	0.30	899.00	0.30	0.02	0.00	0.34	0.00	0.02
t_R_BF	400.00	0.30	899.00	0.30	0.01	0.00	0.13	0.00	0.01
t_R_BF	50.00	0.50	900.00	0.50	0.02	0.00	1.00	0.00	0.02
t_R_BF	100.00	0.50	900.00	0.50	0.01	0.00	0.69	0.00	0.01
t_R_BF	400.00	0.50	900.00	0.50	0.01	0.00	0.18	0.00	0.01
t_R_NL	50.00	0.10	892.00	0.11	0.02	0.01	5.16	0.00	0.02
t_R_NL	100.00	0.10	893.00	0.10	0.01	0.00	3.36	0.00	0.01
t_R_NL	400.00	0.10	890.00	0.10	0.01	0.00	0.10	0.00	0.01
t_R_NL	50.00	0.30	897.00	0.30	0.02	0.00	1.49	0.00	0.02
t_R_NL	100.00	0.30	898.00	0.30	0.01	0.00	0.53	0.00	0.01
t_R_NL	400.00	0.30	899.00	0.30	0.01	0.00	0.13	0.00	0.01
t_R_NL	50.00	0.50	900.00	0.50	0.02	0.00	1.00	0.00	0.02
t_R_NL	100.00	0.50	895.00	0.50	0.01	0.00	0.67	0.00	0.01
t_R_NL	400.00	0.50	898.00	0.50	0.00	0.00	0.18	0.00	0.00
t_R_NM	50.00	0.10	900.00	0.10	0.02	0.00	3.13	0.00	0.02
t_R_NM	100.00	0.10	900.00	0.10	0.02	0.00	1.82	0.00	0.02
t_R_NM	400.00	0.10	896.00	0.10	0.02	-0.00	-2.37	0.00	0.02
t_R_NM	50.00	0.30	900.00	0.30	0.02	0.00	1.39	0.00	0.02
t_R_NM	100.00	0.30	900.00	0.30	0.02	0.00	0.42	0.00	0.02
t_R_NM	400.00	0.30	900.00	0.30	0.01	0.00	0.02	0.00	0.01
t_R_NM	50.00	0.50	900.00	0.50	0.02	0.00	1.00	0.00	0.02
t_R_NM	100.00	0.50	900.00	0.50	0.01	0.00	0.69	0.00	0.01
t_R_NM	400.00	0.50	900.00	0.50	0.01	0.00	0.17	0.00	0.01
t_F_ML	50.00	0.10	900.00	0.10	0.03	0.00	4.29	0.00	0.03
t_F_ML	100.00	0.10	900.00	0.10	0.02	0.00	4.32	0.00	0.02
t_F_ML	400.00	0.10	900.00	0.10	0.01	0.00	0.06	0.00	0.01
t_F_ML	50.00	0.30	900.00	0.30	0.04	0.00	0.64	0.00	0.04
t_F_ML	100.00	0.30	900.00	0.30	0.03	0.00	0.19	0.00	0.03
t_F_ML	400.00	0.30	900.00	0.30	0.01	-0.00	-0.19	0.00	0.01
t_F_ML	50.00	0.50	900.00	0.50	0.04	0.00	0.63	0.00	0.04
t_F_ML	100.00	0.50	900.00	0.50	0.02	-0.00	-0.14	0.00	0.02
t_F_ML	400.00	0.50	900.00	0.50	0.01	0.00	0.09	0.00	0.01
t_F_KS	50.00	0.10	900.00	0.11	0.02	0.01	5.15	0.00	0.02
t_F_KS	100.00	0.10	900.00	0.10	0.01	0.00	3.43	0.00	0.01
t_F_KS	400.00	0.10	900.00	0.10	0.01	0.00	0.10	0.00	0.01
t_F_KS	50.00	0.30	900.00	0.30	0.02	0.00	0.96	0.00	0.02
t_F_KS	100.00	0.30	900.00	0.30	0.01	0.00	0.56	0.00	0.01

t_F_KS	400.00	0.30	900.00	0.30	0.01	0.00	0.13	0.00	0.01
t_F_KS	50.00	0.50	900.00	0.50	0.03	0.00	0.69	0.00	0.03
t_F_KS	100.00	0.50	900.00	0.50	0.01	0.00	0.70	0.00	0.01
t_F_KS	400.00	0.50	900.00	0.50	0.01	0.00	0.17	0.00	0.01
t_F_CS	50.00	0.10	900.00	0.10	0.03	-0.00	-1.49	0.00	0.03
t_F_CS	100.00	0.10	900.00	0.10	0.02	0.00	2.18	0.00	0.02
t_F_CS	400.00	0.10	900.00	0.10	0.01	-0.00	-0.67	0.00	0.01
t_F_CS	50.00	0.30	900.00	0.30	0.06	-0.00	-1.27	0.00	0.06
t_F_CS	100.00	0.30	900.00	0.30	0.03	0.00	0.09	0.00	0.03
t_F_CS	400.00	0.30	900.00	0.30	0.01	0.00	0.04	0.00	0.01
t_F_CS	50.00	0.50	900.00	0.49	0.08	-0.01	-1.57	0.01	0.08
t_F_CS	100.00	0.50	900.00	0.50	0.02	0.00	0.04	0.00	0.02
t_F_CS	400.00	0.50	900.00	0.50	0.01	-0.00	-0.01	0.00	0.01

6.4 Drift Parameter ν

Table 12: Descriptive statistics and parameter recovery performance measures for the drift parameter ν. Notes as in Table 9 and: the relative bias cannot be computed for $\nu = 0$, the respective entries are indicated by (+/−) Inf (for infinity would result after division by zero).

method	trials	true	n	mean	sd	bias	relbias	mse	rmse
v_R_EZ	50.00	-1.00	540.00	-0.94	1.42	0.06	-5.66	2.01	1.42
v_R_EZ	100.00	-1.00	540.00	-0.97	1.42	0.03	-3.46	2.02	1.42
v_R_EZ	400.00	-1.00	540.00	-0.94	1.34	0.06	-6.00	1.80	1.34
v_R_EZ	50.00	-0.50	540.00	-0.50	1.28	-0.00	0.32	1.63	1.27
v_R_EZ	100.00	-0.50	540.00	-0.47	1.30	0.03	-6.43	1.69	1.30
v_R_EZ	400.00	-0.50	540.00	-0.46	1.28	0.04	-7.34	1.64	1.28
v_R_EZ	50.00	0.00	540.00	0.00	1.44	0.00	Inf	2.07	1.44
v_R_EZ	100.00	0.00	540.00	0.02	1.34	0.02	Inf	1.80	1.34
v_R_EZ	400.00	0.00	540.00	0.01	1.29	0.01	Inf	1.65	1.28
v_R_EZ	50.00	0.50	540.00	0.50	1.51	0.00	0.18	2.26	1.50
v_R_EZ	100.00	0.50	540.00	0.51	1.32	0.01	2.34	1.73	1.31
v_R_EZ	400.00	0.50	540.00	0.46	1.30	-0.04	-7.87	1.69	1.30
v_R_EZ	50.00	1.00	540.00	0.99	1.54	-0.01	-1.17	2.36	1.54
v_R_EZ	100.00	1.00	540.00	0.92	1.41	-0.08	-8.29	1.99	1.41
v_R_EZ	400.00	1.00	540.00	0.91	1.30	-0.09	-8.75	1.71	1.31
v_R_BY	50.00	-1.00	540.00	-0.86	0.39	0.14	-13.75	0.17	0.41
v_R_BY	100.00	-1.00	540.00	-0.94	0.30	0.06	-5.73	0.09	0.30
v_R_BY	400.00	-1.00	540.00	-0.98	0.17	0.02	-1.76	0.03	0.17
v_R_BY	50.00	-0.50	537.00	-0.46	0.37	0.04	-7.56	0.14	0.37
v_R_BY	100.00	-0.50	537.00	-0.47	0.33	0.03	-5.25	0.11	0.33
v_R_BY	400.00	-0.50	501.00	-0.50	0.15	0.00	-0.04	0.02	0.15
v_R_BY	50.00	0.00	537.00	0.00	0.36	0.00	Inf	0.13	0.36
v_R_BY	100.00	0.00	519.00	-0.00	0.29	-0.00	-Inf	0.08	0.29
v_R_BY	400.00	0.00	507.00	0.01	0.16	0.01	Inf	0.03	0.16
v_R_BY	50.00	0.50	531.00	0.45	0.37	-0.05	-9.64	0.14	0.38
v_R_BY	100.00	0.50	528.00	0.48	0.27	-0.02	-3.16	0.07	0.27
v_R_BY	400.00	0.50	507.00	0.50	0.15	0.00	0.65	0.02	0.15
v_R_BY	50.00	1.00	540.00	0.86	0.38	-0.14	-13.90	0.16	0.41
v_R_BY	100.00	1.00	540.00	0.90	0.31	-0.10	-9.70	0.10	0.32
v_R_BY	400.00	1.00	534.00	0.97	0.16	-0.03	-2.68	0.03	0.16
v_R_BF	50.00	-1.00	540.00	-0.96	0.55	0.04	-3.79	0.30	0.55
v_R_BF	100.00	-1.00	540.00	-0.99	0.52	0.01	-0.87	0.27	0.52
v_R_BF	400.00	-1.00	540.00	-0.95	0.34	0.05	-5.00	0.12	0.35
v_R_BF	50.00	-0.50	540.00	-0.49	0.54	0.01	-1.70	0.29	0.54
v_R_BF	100.00	-0.50	540.00	-0.48	0.41	0.02	-4.20	0.16	0.41
v_R_BF	400.00	-0.50	540.00	-0.47	0.28	0.03	-5.04	0.08	0.28
v_R_BF	50.00	0.00	540.00	0.03	0.48	0.03	Inf	0.23	0.48
v_R_BF	100.00	0.00	539.00	0.02	0.34	0.02	Inf	0.11	0.34
v_R_BF	400.00	0.00	540.00	0.03	0.20	0.03	Inf	0.04	0.20
v_R_BF	50.00	0.50	540.00	0.51	0.48	0.01	1.21	0.23	0.48
v_R_BF	100.00	0.50	540.00	0.50	0.33	0.00	0.94	0.11	0.33
v_R_BF	400.00	0.50	538.00	0.49	0.19	-0.00	-0.99	0.04	0.19
v_R_BF	50.00	1.00	540.00	1.00	0.55	-0.00	-0.14	0.30	0.55
v_R_BF	100.00	1.00	540.00	0.95	0.43	-0.05	-4.52	0.19	0.43
v_R_BF	400.00	1.00	540.00	0.94	0.27	-0.06	-5.63	0.08	0.27
v_R_NL	50.00	-1.00	539.00	-1.02	0.50	-0.02	1.66	0.25	0.50
v_R_NL	100.00	-1.00	539.00	-1.04	0.34	-0.04	3.62	0.12	0.34
v_R_NL	400.00	-1.00	539.00	-1.01	0.18	-0.01	0.86	0.03	0.18
v_R_NL	50.00	-0.50	540.00	-0.54	0.52	-0.04	8.29	0.28	0.53
v_R_NL	100.00	-0.50	536.00	-0.51	0.38	-0.01	2.40	0.14	0.38
v_R_NL	400.00	-0.50	539.00	-0.51	0.16	-0.01	2.00	0.03	0.16
v_R_NL	50.00	0.00	536.00	0.01	0.50	0.01	Inf	0.25	0.50
v_R_NL	100.00	0.00	534.00	-0.00	0.35	-0.00	-Inf	0.12	0.35
v_R_NL	400.00	0.00	536.00	0.01	0.16	0.01	Inf	0.03	0.16
v_R_NL	50.00	0.50	537.00	0.54	0.52	0.04	7.91	0.27	0.52
v_R_NL	100.00	0.50	539.00	0.54	0.34	0.04	7.41	0.11	0.34
v_R_NL	400.00	0.50	535.00	0.52	0.15	0.02	3.34	0.02	0.15
v_R_NL	50.00	1.00	537.00	1.04	0.52	0.04	3.54	0.28	0.52

v_R_NL	100.00	1.00	538.00	1.00	0.37	-0.00	-0.04	0.14	0.37
v_R_NL	400.00	1.00	538.00	1.00	0.17	-0.00	-0.20	0.03	0.16
v_R_NM	50.00	-1.00	540.00	-1.00	0.51	-0.00	0.34	0.26	0.51
v_R_NM	100.00	-1.00	540.00	-1.03	0.35	-0.03	3.14	0.12	0.35
v_R_NM	400.00	-1.00	539.00	-1.00	0.20	-0.00	0.08	0.04	0.20
v_R_NM	50.00	-0.50	540.00	-0.53	0.53	-0.03	6.10	0.28	0.53
v_R_NM	100.00	-0.50	540.00	-0.51	0.38	-0.01	2.24	0.15	0.38
v_R_NM	400.00	-0.50	538.00	-0.50	0.18	-0.00	0.34	0.03	0.18
v_R_NM	50.00	0.00	540.00	0.01	0.50	0.01	Inf	0.25	0.50
v_R_NM	100.00	0.00	540.00	0.01	0.35	0.01	Inf	0.13	0.35
v_R_NM	400.00	0.00	539.00	0.02	0.16	0.02	Inf	0.03	0.16
v_R_NM	50.00	0.50	540.00	0.54	0.52	0.04	7.86	0.27	0.52
v_R_NM	100.00	0.50	540.00	0.54	0.34	0.04	7.06	0.12	0.34
v_R_NM	400.00	0.50	540.00	0.52	0.15	0.02	3.41	0.02	0.16
v_R_NM	50.00	1.00	540.00	1.04	0.52	0.04	3.51	0.27	0.52
v_R_NM	100.00	1.00	540.00	1.00	0.37	-0.00	-0.12	0.14	0.37
v_R_NM	400.00	1.00	540.00	1.00	0.17	-0.00	-0.49	0.03	0.17
v_F_ML	50.00	-1.00	540.00	-1.11	0.72	-0.11	11.17	0.52	0.72
v_F_ML	100.00	-1.00	540.00	-1.12	0.51	-0.12	11.51	0.28	0.52
v_F_ML	400.00	-1.00	540.00	-1.02	0.24	-0.02	2.18	0.06	0.24
v_F_ML	50.00	-0.50	540.00	-0.58	0.69	-0.08	16.16	0.49	0.70
v_F_ML	100.00	-0.50	540.00	-0.52	0.46	-0.02	3.66	0.21	0.46
v_F_ML	400.00	-0.50	540.00	-0.52	0.20	-0.02	4.07	0.04	0.20
v_F_ML	50.00	0.00	540.00	0.04	0.66	0.04	Inf	0.44	0.66
v_F_ML	100.00	0.00	540.00	0.00	0.39	0.00	Inf	0.15	0.39
v_F_ML	400.00	0.00	540.00	0.01	0.19	0.01	Inf	0.04	0.19
v_F_ML	50.00	0.50	540.00	0.66	0.83	0.16	31.54	0.71	0.84
v_F_ML	100.00	0.50	540.00	0.57	0.39	0.07	13.60	0.16	0.40
v_F_ML	400.00	0.50	540.00	0.53	0.19	0.03	5.88	0.04	0.19
v_F_ML	50.00	1.00	540.00	1.15	0.91	0.15	15.10	0.85	0.92
v_F_ML	100.00	1.00	540.00	1.09	0.56	0.09	9.38	0.32	0.57
v_F_ML	400.00	1.00	540.00	1.01	0.22	0.01	1.19	0.05	0.22
v_F_KS	50.00	-1.00	540.00	-1.01	0.49	-0.01	0.52	0.24	0.49
v_F_KS	100.00	-1.00	540.00	-1.04	0.34	-0.04	3.68	0.12	0.34
v_F_KS	400.00	-1.00	540.00	-1.01	0.18	-0.01	0.86	0.03	0.18
v_F_KS	50.00	-0.50	540.00	-0.53	0.54	-0.03	6.12	0.29	0.54
v_F_KS	100.00	-0.50	540.00	-0.51	0.38	-0.01	2.80	0.14	0.38
v_F_KS	400.00	-0.50	540.00	-0.51	0.16	-0.01	1.90	0.03	0.16
v_F_KS	50.00	0.00	540.00	0.03	0.54	0.03	Inf	0.29	0.54
v_F_KS	100.00	0.00	540.00	-0.00	0.35	-0.00	-Inf	0.12	0.35
v_F_KS	400.00	0.00	540.00	0.01	0.16	0.01	Inf	0.03	0.16
v_F_KS	50.00	0.50	540.00	0.55	0.52	0.05	10.81	0.27	0.52
v_F_KS	100.00	0.50	540.00	0.54	0.34	0.04	7.37	0.11	0.34
v_F_KS	400.00	0.50	540.00	0.52	0.15	0.02	3.20	0.02	0.15
v_F_KS	50.00	1.00	540.00	1.04	0.52	0.04	3.62	0.27	0.52
v_F_KS	100.00	1.00	540.00	1.00	0.37	-0.00	-0.03	0.14	0.37
v_F_KS	400.00	1.00	540.00	1.00	0.17	-0.00	-0.20	0.03	0.17
v_F_CS	50.00	-1.00	540.00	-1.17	0.86	-0.17	16.90	0.76	0.87
v_F_CS	100.00	-1.00	540.00	-1.05	0.47	-0.05	5.00	0.22	0.47
v_F_CS	400.00	-1.00	540.00	-0.99	0.20	0.01	-1.03	0.04	0.20
v_F_CS	50.00	-0.50	540.00	-0.67	0.78	-0.17	33.66	0.64	0.80
v_F_CS	100.00	-0.50	540.00	-0.52	0.44	-0.02	3.38	0.19	0.44
v_F_CS	400.00	-0.50	540.00	-0.50	0.18	0.00	-0.32	0.03	0.18
v_F_CS	50.00	0.00	540.00	-0.06	1.05	-0.06	-Inf	1.09	1.05
v_F_CS	100.00	0.00	540.00	-0.00	0.36	-0.00	-Inf	0.13	0.36
v_F_CS	400.00	0.00	540.00	0.01	0.18	0.01	Inf	0.03	0.18
v_F_CS	50.00	0.50	540.00	0.60	0.84	0.10	19.43	0.71	0.84
v_F_CS	100.00	0.50	540.00	0.50	0.40	-0.00	-0.18	0.16	0.40
v_F_CS	400.00	0.50	540.00	0.51	0.16	0.01	1.26	0.03	0.16
v_F_CS	50.00	1.00	540.00	1.18	1.02	0.18	17.63	1.07	1.04
v_F_CS	100.00	1.00	540.00	1.01	0.48	0.01	1.28	0.23	0.48
v_F_CS	400.00	1.00	540.00	0.97	0.18	-0.03	-2.84	0.03	0.19
